# An Updated and Comprehensive Review of *Phellodendri amurensis* Cortex: Ethnobotany, Geographical Distribution, Phytochemistry, Quality Control, and Pharmacology

**DOI:** 10.3390/molecules31081318

**Published:** 2026-04-17

**Authors:** Kang Li, Chunqi Song, Xin Tan, Yang Zhang, Hao Zang, Xingzun Zhu

**Affiliations:** 1 School of Landscape Architecture, Changchun University, Changchun 130022, China; 241203454@mails.ccu.edu.cn (K.L.); 241203448@mails.ccu.edu.cn (C.S.); 251203471@mails.ccu.edu.cn (X.T.); 251201449@mails.ccu.edu.cn (Y.Z.); 2 School of Pharmacy and Medicine, Tonghua Normal University, Tonghua 134002, China

**Keywords:** *Phellodendri amurensis* Cortex, ethnobotanical uses, chemical constituents, geographic distribution, quality control, pharmacological activities

## Abstract

*Phellodendri amurensis* Cortex is the dried bark of the cork tree (*Phellodendron amurense* Rupr.) from the Rutaceae family, and possesses traditional efficacy in clearing heat, drying dampness, purging fire, relieving steaming sensations, detoxifying, and healing sores. Clinically, it is commonly used for treating symptoms such as damp-heat diarrhea and dysentery, jaundice with reddish urine, leukorrhea with vaginal itching, painful and difficult urination due to heat strangury, flaccidity and weakness of the lower limbs, bone-steaming and consumptive fever, night sweats and seminal emission, sores, ulcers, swellings, and toxins, eczema, damp sores, and urinary tract infections. Modern pharmacological studies have further revealed its diverse bioactivities, including antioxidant, antibacterial, anti-inflammatory, immunosuppressive, and anticancer effects. To provide an updated and comprehensive review of the research into *Phellodendri amurensis* Cortex, this study conducted a thorough literature search and analysis based on databases such as SciFinder, Web of Science, and China National Knowledge Infrastructure. The review integrates information on the plant’s botanical characteristics, geographical distribution, traditional applications, chemical components, quality control methods, and pharmacological effects to present a current and holistic overview of its research status. To date, approximately 170 compounds have been isolated and identified from *Phellodendri amurensis* Cortex, primarily including alkaloids, phenolics, terpenoids, sterols, lignans, flavonoids, and others. Among these, alkaloids exhibit significant antioxidant and anti-inflammatory activities and demonstrate potential pharmacological value in antibacterial, anticancer, hypoglycemic, and multi-organ protective effects. Although substantial foundational research exists, the mechanisms of action and quality control of *Phellodendri amurensis* Cortex require further in-depth exploration. Future efforts should focus on clarifying its pharmacodynamic material basis, uncovering new targets and pathways, and improving analytical methods for component analysis and quality control to advance the scientific development and rational utilization of this medicinal material.

## 1. Introduction

**Figure 1:**
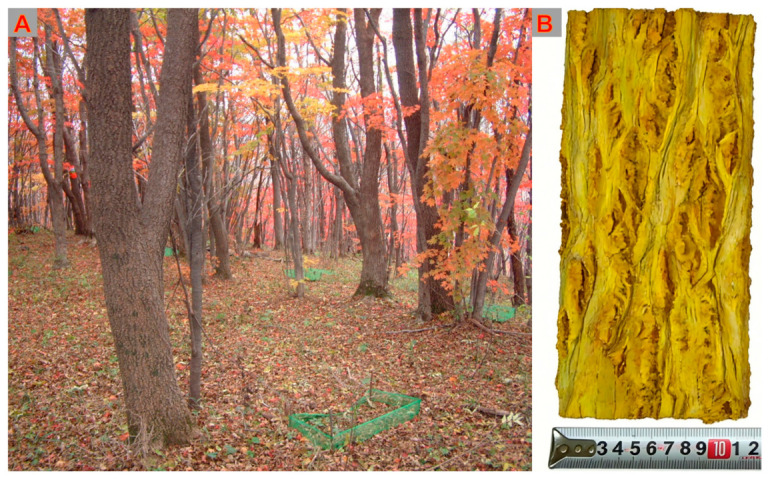
Morphology of *Phellodendron amurense* Rupr. and its cortex. (**A**) *Phellodendron amurense* Rupr.; (**B**) *Phellodendri amurensis* Cortex.

*Phellodendri amurensis* Cortex (PAC) (Figure 1A) is the dried bark of *Phellodendron amurense* Rupr. (*P. amurense*) (Figure 1B), a plant belonging to the Rutaceae family. It is an important bark-derived traditional Chinese medicine (TCM) originating from Northeast China, and is renowned for its medicinal value in clearing heat, drying dampness, purging fire, relieving steaming sensations, detoxifying, and healing sores. To date, various active components have been isolated from PAC, including alkaloids, phenolics, terpenoids, phenylpropanoids, sterols, lignans, and flavonoids. Modern pharmacological studies have demonstrated that its extracts and monomeric compounds possess antibacterial, anti-inflammatory, immunosuppressive, anticancer, and neuroprotective effects, with anti-inflammatory and anticancer activities being particularly prominent.

Historically, PAC and *Phellodendri chinensis* Cortex (PCC) were not distinguished for a long time and were collectively referred to as “Huangbai”. Consequently, independent records of PAC are absent in ancient texts, and most historical processing records refer to PCC [1]. It was not until the 2005 edition of the Chinese Pharmacopoeia [2] that, recognizing their close but distinct botanical origins, the two began to be listed separately. The 2025 edition [3] continues this practice, listing PAC and PCC as two independent medicinal materials. Specific records dedicated to PAC emerged relatively late, first appearing in the 1941 Korean Pharmacopoeia and the 1957 Liaoning Authentic Medicinal Materials [4,5], after which PAC gradually gained recognition as an important medicinal variety.

Research and development of proprietary Chinese medicines primarily containing PAC have made notable progress. A variety of formulations incorporating this ingredient have been successively developed and applied in clinical settings, including PAC Capsules [6], Qinbai Ointment [7], and Shenbai Xiaoban Lotion [8]. Among these, PAC Capsules have been widely adopted in the Chinese market due to their notable efficacy against acne, eczema, and urticaria [6].

## 2. Methods

To ensure the reliability and integrity of the information gathered for this review, we conducted a systematic literature search across multiple databases, including Web of Science, SciFinder, PubMed, ProQuest, and the China National Knowledge Infrastructure. The search period covered from 1958 to March 2026. We used a combination of specific keywords—such as phytochemistry, alkaloid, ethnobotanical use, geographical distribution, quality control, pharmacology, anti-cancer, antioxidant, anti-inflammatory, and toxicology—in conjunction with PAC. The retrieved literature included peer-reviewed journal articles, Ph.D. and master’s theses, conference papers, and classic texts on Chinese herbal medicines.

The inclusion criteria were as follows: (1) primary research articles focusing on the geographical distribution, phytochemistry, quality control, or pharmacology of PAC; (2) studies published in English, Chinese, or Japanese; and (3) literature providing extractable data or conclusive findings relevant to the scope of this review.

The exclusion criteria were as follows: (1) studies not primarily focusing on the geographical distribution, phytochemistry, quality control, or pharmacology of PAC; (2) non-primary research articles (e.g., reviews, opinion pieces, editorials, patents); (3) articles with inaccessible full texts or data that could not be extracted or analyzed (e.g., insufficiently detailed conference abstracts); (4) publications in languages other than English, Chinese, or Japanese; and (5) duplicate reports (in which case, only the most complete or recent publication was retained).

To minimize bias, the study selection process was conducted independently by two authors. Any disagreements were resolved through discussion.

## 3. Geographical Distribution

*P. amurense* is native to Northeast and North China, with its range extending to Henan, northern Anhui, Ningxia, Inner Mongolia, as well as the Korean Peninsula, Japan, and the Russian Far East [9]. Globally, its distribution spans Central Asia, Eastern Europe, and parts of North America, and it has been introduced to regions such as Bulgaria, Romania, the northeastern United States, and southeastern Canada (https://powo.science.kew.org/, accessed on 18 March 2026). The species thrives in mixed mountain forests and river valleys, exhibiting high adaptability, cold tolerance, and a preference for sunny habitats. While its wood is valued for fine furniture, its inner bark is the source of the TCM “PAC,” which is widely used in clinical practice [10,11]. However, since the 1960s, wild populations have experienced severe decline due to over-harvesting of bark, deforestation, land development, and natural disasters, bringing the resource near exhaustion [12]. As a result, *P. amurense* has been listed as a National Second-Class Key Protected Plant in China since 1999 and assessed as Vulnerable in the China Species Red List [13,14].

## 4. Application Status

### 4.1. Traditional Applications

Ancient texts document that the main efficacy of Huangbai is primarily to treat stagnant qi heat in the five viscera and the gastrointestinal tract, while also clearing heat, drying dampness, purging fire, and detoxifying. Its therapeutic scope has continuously expanded and become more specific over time. The earliest record can be found in the Shennong Bencao Jing (Divine Farmer’s Materia Medica), which states that it “treats stagnant qi heat in the five viscera and the gastrointestinal tract, jaundice, hemorrhoids, stops dysentery and diarrhea, treats metrorrhagia and leukorrhea in women, and erosive ulcers.” Later medical texts gradually added symptoms such as “red and hot skin eruptions, heat-induced redness and pain in the eyes, mouth ulcers, bone-steaming tidal fever, night sweats, seminal emission, sores, itching, swelling and toxicity, eczema, and itching,” highlighting its functions of clearing heat, drying dampness, purging fire, and detoxifying, while further refining its clinical applications. The Chinese Pharmacopoeia records that PAC has the effects of clearing heat and drying dampness, purging fire and relieving steaming, and detoxifying to heal sores. It is applicable to damp-heat dysentery, jaundice with reddish urine, leukorrhea with vaginal itching, heat strangury with painful urination, beriberi and flaccidity, bone-steaming tidal fever, night sweats, seminal emission, sores, itching, swelling, and toxicity, eczema and damp sores, and urinary tract infections. Salt-processed PAC is particularly effective for nourishing yin and reducing fire, making it suitable for yin deficiency with fire excess, night sweats, and bone-steaming fever [3].

The processing methods for PAC primarily include honey processing, Huangjiu processing, and salt processing. Before processing, impurities must be removed, and the herb should be washed, flattened, cut into slices or strips, and sun-dried. Honey-processed PAC is often used for mouth ulcers. Physicians from the Ming and Qing dynasties believed that honey-frying could harmonize the middle and protect the stomach. As recorded in Lei Gong Pao Zhi Lun (Lei Gong’s Treatise on Medicinal Processing): “When preparing PAC, use a knife to remove the coarse bark, soak it in raw honey water for half a day, drain, and sun-dry. Then, apply honey and roast over gentle and then strong fire until the honey is completely consumed. For every five liang of herb processed, use three liang of honey.” Salt-processed PAC typically involves mixing 50 kg of PAC slices with 1.25 kg of salt, which has been dissolved in boiled water and clarified, to moisten the herb. It is then lightly stir-fried over gentle heat and left to air-dry. Huangjiu-processed PAC is prepared by stir-frying 50 kg of PAC slices with 5 kg of Huangjiu [15,16].

### 4.2. Modern Applications

#### 4.2.1. Current Status of Clinical Application

In clinical practice, PAC is rarely used alone and typically serves as the monarch or minister drug in compound formulas. For example, the classic formula “Zhibai Dihuang Wan” is developed from Liuwei Dihuang Wan by adding PAC and *Anemarrhenae rhizoma*, achieving the combined effect of nourishing yin and reducing fire. The traditional Liuwei Dihuang Wan primarily focuses on nourishing kidney yin and is mainly indicated for symptoms such as debility, fatigue, and lower back pain. With the addition of *Anemarrhenae rhizoma* and PAC, it not only nourishes kidney yin but also reduces fire, making it more suitable for yin deficiency accompanied by overt “excessive fire” symptoms. The Chinese Pharmacopoeia records that PAC has the effect of clearing fire and eliminating steaming [3], which clearly demonstrates the significant role PAC plays in this formula.

In recent years, its application value of PAC in specific diseases has gained increasing attention. For instance, research on the “*Anemarrhenae rhizoma*-PAC” herb pair continues to deepen in the treatment of precocious puberty in children, and its role in intervening in chronic prostatitis has also been explored. The alkaloids in PAC significantly improved capsaicin-induced eye movement and activity scores in a rat model of chronic nonbacterial prostatitis (CNP), reduced prostatic protein exudation, and alleviated histopathological damage, indicating their potential to interfere with the pathological process of CNP [17]. Additionally, the proprietary Chinese medicine “Guan Huang Mu Granules”, which is mainly composed of PAC, has been listed as a secondary protected TCM variety, reflecting national-level recognition of its clinical value.

Over the past two years, research on PAC has primarily focused on three interconnected areas. First, studies utilizing modern techniques such as network pharmacology and molecular docking have progressively elucidated its “multi-component, multi-target” mechanisms of action against PAC complex diseases such as gout. For instance, active constituents in PAC have been shown to exert anti-gout effects by targeting key proteins including protein kinase B (AKT) serine/threonine kinase 1, tumor necrosis factor-alpha (TNF-*α*), and interleukin (IL)-6, thereby modulating critical signaling pathways including the phosphatidylinositol 3-kinase-protein kinase B and mitogen-activated protein kinase (MAPK) pathways [18]. Second, the efficacy of these key components has been further validated through in vitro cell models (e.g., Tohoku Hospital Pediatrics-1 (THP-1) cells) and in vivo animal studies (e.g., rat models of gouty nephropathy). One study, for example, confirmed the therapeutic role and underlying mechanisms of PAC polysaccharides in ameliorating gouty nephropathy in rats [19]. Finally, as pharmacological insights deepen, quality control and standardization of the herb have gained significant research attention. Ensuring the consistency and clinical reliability of PAC materials relies on a dual approach: species identification by deoxyribonucleic acid (DNA) barcoding and quantification of marker compounds (e.g., berberine) via high-performance liquid chromatography (HPLC).

#### 4.2.2. Purchase Channels and Market Overview

In China, PAC is primarily distributed through business-to-business channels along the supply chain of “production areas → medicinal material wholesale markets (e.g., Hebei Anguo, Anhui Bozhou, Guangxi Yulin) → medical institutions/decoction piece manufacturers.” As a prescription-only ingredient in TCM, raw PAC decoction pieces are almost exclusively dispensed through TCM hospitals, TCM pharmacies, and clinics based on physicians’ prescriptions, rather than being directly available to consumers through over-the-counter retail channels.

Regarding market prices, data from the three major wholesale markets indicate that PAC prices have remained at relatively elevated levels in recent years, generally ranging from approximately 65 to 90 Renminbi per kg, with regional and seasonal variations. This price trend reflects the ongoing supply pressure resulting from the vulnerable status of wild *P. amurense* populations and the long cultivation cycle (10–15 years) required for bark harvesting.

It should be noted that while this practical background information provides context for the current availability of PAC as a medicinal material, the core scientific focus of this review remains on the phytochemistry, pharmacology, and quality control of PAC. Detailed market analysis is beyond the scope of this article.

#### 4.2.3. Current Application of PAC in Chinese Patent Medicines

Patented Chinese medicines containing PAC that are officially recorded in the Chinese Pharmacopoeia (2010 edition and subsequent editions) primarily include Jingfukang Granules, Qianlietong Tablets, Niaosaitong Tablets, Wushe Zhiyang Pills, Yujin Yinxie Tablets, and Gujing Pills. In recent years, newly developed drugs such as Yishen Juanbi Capsules, Shenbai Capsules, Jiawei Pipa Qingfei Capsules, Rongcao Qingdai Tablets, Shuangshitonglin Capsules, Shuangbai San, Compound PAC Dropping Pills, Qinyu Shaoshang Solution, Bushen Pills, Yufu Lotion, Juanling Tincture, Keyin Cream, Shuangbai Spray Film, Sanhuang Suppositories, Meiyan Hufu Powder, and Hufan Huagou Ointment have further expanded the application scope of PAC. The dosage forms of the aforementioned drugs are diverse, covering granules, tablets, capsules, tinctures, creams, films, suppositories, and powders, reflecting the excellent compatibility and broad applicability of PAC in pharmaceutical formulation development.

## 5. Other Applications

**Figure 2:**
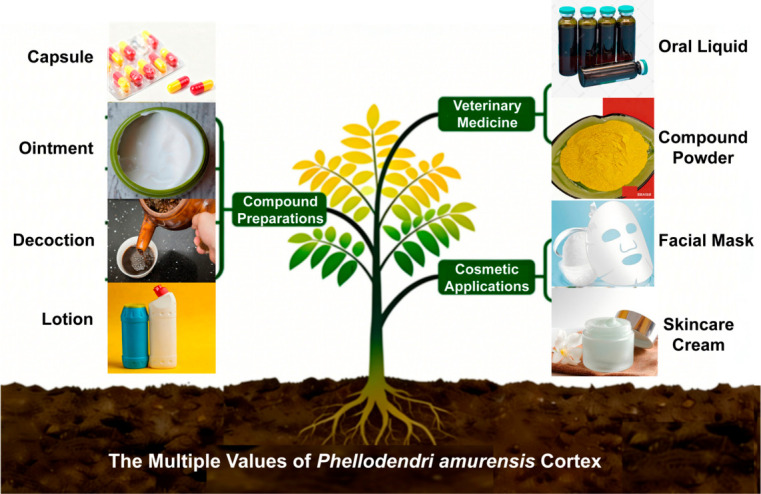
The multiple values of *Phellodendri amurensis* Cortex.

PAC has multiple uses (Figure 2), as detailed below.

### 5.1. Development of Compound Preparations of PAC

Since the 2005 edition of the Chinese Pharmacopoeia, PAC and PCC have been recorded as two distinct medicinal materials. PAC is characterized by its cold nature and bitter taste, and it primarily targets the kidney and bladder meridians. It possesses the functions of clearing heat, drying dampness, purging fire, relieving steaming sensations, detoxifying, and healing sores. Classified as a heat-clearing and dampness-drying herb, it is widely used in clinical practice. The common dosage is 3–12 g, with appropriate amounts for external use. It is indicated for conditions such as damp-heat dysentery, jaundice with reddish urine, painful and difficult urination due to heat, leukorrhea with vaginal itching, beriberi with weakness, bone-steaming fever, eczema, damp sores, and ulcerative swelling and toxins. Salt-processed PAC is more effective in nourishing yin and reducing fire, and is often used for conditions of yin deficiency with fire effulgence, such as night sweats and bone-steaming fever. Based on these functions, PAC has become an important raw material for various Chinese patent medicines, contributing substantially to the treatment of relevant diseases [20]. For example, PAC Capsule is composed solely of PAC and is recorded in the fifth volume of the Ministry of Health Drug Standards: Chinese Patent Medicines. Its active ingredients mainly include berberine, jatrorrhizine, and phellodendrine. Research has demonstrated that this preparation exhibits significant efficacy against acne, eczema, and urticaria [6].

Qinbai Ointment is a traditional topical preparation commonly used in the dermatology and surgery departments of Beijing Hospital of TCM affiliated with Capital Medical University. It is composed of PAC and *Scutellaria baicalensis*. Among these components, PAC exerts the effects of clearing heat, removing toxicity, promoting diuresis, and reducing swelling. The functions of Qinbai Ointment include clearing heat, detoxifying, promoting diuresis to eliminate dampness, and reducing swelling. It is indicated for heat-toxin sores, carbuncles, and cellulitis, and is frequently employed in the management of skin disorders, including psoriasis, folliculitis, dermatitis, and eczema [7].

PAC Decoction is prepared by decocting a single herb, PAC. It can be used to treat ulcerative stomatitis, which is often caused by accumulated heat in the heart and spleen that ascends to fumigate the oral cavity. Research has confirmed that this decoction is effective and safe, with its mechanism of action related to PAC’s abilities to clear damp-heat, purge fire and toxins, provide antibacterial and anti-inflammatory effects, and protect platelets [21].

Shenbai Xiaoban Lotion is a Chinese herbal compound formulation derived from clinical experience at the Fourth Hospital of Hebei Medical University. It is composed of more than ten medicinal herbs, including *Sophora flavescens*, PAC, *Cnidium monnieri*, and *Epimedium brevicornum*. This preparation is mainly used clinically for vulvar itching, vulvar leukoplakia, and various types of vaginitis. In this formulation, PAC primarily functions to clear heat, dry dampness, kill parasites, and relieve itching [8].

### 5.2. Application of PAC in Veterinary Medicine

PAC, due to its antibacterial and antiviral (e.g., against influenza virus) effects, is widely utilized in veterinary drug development [22]. In 2013, “Gaoqin Oral Liquid,” jointly developed by China Agricultural University, Chengdu Qiankun Animal Pharmaceutical Co., Ltd., and other institutions, received the Category III New Veterinary Drug Registration Certificate from the National Ministry of Agriculture. This oral liquid is composed of eight Chinese medicinal herbs, including gypsum, *Scutellaria baicalensis*, PAC, *Rehmannia glutinosa*, *Atractylodes lancea*, and *Gardenia jasminoides* [23]. It effectively reduces bacterial resistance and is primarily used in veterinary clinical practice for the prevention and treatment of swine febrile diseases caused by viral infections. In this formulation, PAC exerts its effects of inhibiting bacteria, clearing heat, detoxifying, purging fire, and relieving steaming sensations. Furthermore, “Compound PAC Powder” (commercial name “Antibacterial King”) is a Chinese–Western integrated preparation that demonstrates good efficacy against these diseases [24]. The core herb is PAC, which is combined with several other Chinese herbal medicines and Western medicines to broaden the antibacterial spectrum and enhance the preventive and therapeutic effects against major bacterial diseases in poultry farms [24]. Among its components, PAC primarily exerts the effects of clearing heat, drying dampness, purging fire, detoxifying, and providing broad-spectrum bacteriostasis. Its key active ingredient is berberine, which exhibits strong inhibitory effects against *Staphylococcus aureus*, *Salmonella pullorum*, *Pasteurella multocida*, *Salmonella gallinarum*, *Escherichia coli*, and *Streptococcus hemolyticus*.

### 5.3. Development of PAC for Cosmetic Applications

Modern pharmacological research has demonstrated that PAC possesses antibacterial, anti-inflammatory, antioxidant, and eczema-relieving effects, leading to its widespread application in cosmetics and daily-use products [25]. For example, the PAC facial mask developed by Shandong Hanfang NewBay Little Red Hat Biotechnology Co., Ltd (Jinan City, China). Ref. [26] significantly improves mild to moderate acne, as the component jatrorrhizine in PAC improves collagen homeostasis and inhibits damage to skin fibroblasts. Wang and colleagues combined PAC extract with other traditional Chinese medicinal ingredients to develop a skincare cream [27]. This product contains winter melon extract, the “Three Huang” extract (PAC, *Scutellaria baicalensis*, and *Rheum palmatum*), rose oil, vitamin E, and vitamin C, among other components. It promotes cell and tissue metabolism, improves local blood circulation, nourishes the skin, and helps maintain skin elasticity and moisture. In this formulation, PAC primarily plays the roles of anti-inflammation, antibacterial activity, soothing redness, and repairing the skin barrier.

## 6. Botanical Description

*P. amurense* is a tree species typically reaching heights of 10 to 20 m. Under optimal conditions, mature specimens can attain a maximum height of 30 m and a trunk diameter of up to 1 m at breast height. Its branches spread widely, and the bark of adult trees features a thick cork layer that is pale gray or grayish-brown, with deep grooves or irregular reticulate fissures. The inner bark is thin, bright yellow, bitter in taste, and sticky. Young twigs are dark purplish-red and glabrous. The leaf axis and petioles are slender, bearing 5–13 leaflets. The leaflets are characteristically thin and papery in texture. Their shape varies from ovate-lanceolate to ovate, ranging from 6–12 cm in length and 2.5–4.5 cm in width. They have a long-acuminate apex and a broadly wedge-shaped or occasionally rounded base, often oblique on one side. The leaf margins exhibit fine blunt teeth and ciliate edges. The upper leaf surface is glabrous or sparsely covered with short hairs along the midvein, while the lower surface is densely covered with long soft hairs only at the base on both sides of the midvein. Before shedding in autumn, the leaves turn from green to bright yellow, and most of the hairs detach. The inflorescence is terminal. The sepals are small and broadly ovate, about 1 mm long. The petals are purplish-green, 3–4 mm in length. In male flowers, the stamens are longer than the petals, and the pistil is short and reduced. The fruit is spherical, approximately 1 cm in diameter, turning blue-black when ripe, and typically bears 5–10 shallow longitudinal grooves, that become more pronounced after drying. Each fruit usually contains 5 seeds. Flowering occurs from May to June, and fruiting from September to October.

## 7. Phytochemistry

Investigations into the chemical makeup of PAC can be traced back to 1926, when Japanese researcher Yoshion Murakami and his colleagues succeeded in isolating berberine and a minor amount of palmatine from the Japanese variety of *P. amurense*. Subsequently, scholars from various countries have reported numerous other compounds. Especially in recent years, with growing awareness of the importance and utilization of this plant, research on its chemical constituents has expanded. To date, 170 compounds have been isolated or identified from the bark of this plant. Importantly, because PAC and PCC were historically not distinguished and were collectively referred to as “Huangbai” in older literature, we have taken special care to ensure that the phytochemical evidence cited in this section is specifically attributable to PAC. For each study included, we verified that the plant material was identified as *P. amurense*. Studies using *P. chinense* or unspecified *Phellodendron* species without clear botanical authentication were excluded from this review. Where a study compared both species, this distinction is explicitly noted. These compounds can be classified into seven categories: alkaloids, phenolics, terpenoids, phenylpropanoids, sterols, lignans, flavonoids, and others. The rich array of bioactive compounds contained in PAC highlights its potential value in drug development and clinical applications.

### 7.1. Alkaloids

Among the compounds derived from *P. amurense*, alkaloids have been the most extensively studied and are the earliest reported constituents. To date, 77 alkaloid components have been identified (Table 1). The extraction and separation methods for alkaloids from *P. amurense* can be summarized into 6 common protocols. Method A (compounds **1**–**11**) involved triple ethanol reflux extraction with 50% ethanol, followed by rotary evaporation concentration, freeze-drying, and pulverization; the dried extract was then dissolved in 50% methanol by ultrasonication and filtered [28]. Method B (compounds **12**–**16**) used triple reflux extraction of chopped raw materials with 80% methanol, followed by separation via silica gel column chromatography [29]. Method C (compounds **17**–**40**) also employed triple reflux extraction with 80% methanol and separation via silica gel column chromatography [30]. Method D (compounds **41**–**48**, **54**–**77**) utilized methanol ultrasonication and centrifugation, followed by separation using HPLC and ion mobility spectrometry [31]. Method E (compounds **49**–**51**) involved methanol reflux extraction of the raw material, after which the methanol extract was suspended in water and extracted with ethyl acetate; the ethyl acetate extract was then separated via silica gel column chromatography and HPLC [32]. Method F (compounds **52**–**53**) used triple reflux extraction with 75% methanol, after which the combined extracts were freeze-dried, and the reconstituted solution was filtered through a 0.22 μm membrane filter [33,34].

### 7.2. Phenolics

Researchers have isolated and identified 21 phenolic compounds from *P. amurense* (Table 2). The extraction and separation methods for phenolics from *P. amurense* can be summarized as follows. Compounds **78**–**80** were obtained by grinding, sieving, and drying the raw material, followed by ultrasonic extraction with 75% ethanol, dissolution in methanol, and filtration [30]. Compounds **81**–**85** and **87**–**88** were extracted with methanol, after which the combined extracts were concentrated under reduced pressure; the obtained syrup was suspended in water and extracted with ethyl acetate, and the ethyl acetate fraction was then separated by silica gel column chromatography [35]. Compounds **86**, **96**, and **97** were extracted with methanol under reflux, after which the methanol extract was suspended in water, extracted with ethyl acetate, and the ethyl acetate extract was separated by silica gel column chromatography and HPLC [32]. Compound **89** was extracted with methanol, followed by sequential partitioning with *n*-hexane, chloroform, and ethanol, and the resulting fractions were then purified by silica gel column chromatography and recrystallization [36]. Compounds **90** and **91** were extracted with methanol and further separated by chromatographic methods including TLC and silica gel column chromatography [37]. Compounds **92** and **98** were subjected to triple reflux extraction with 75% methanol, after which the combined extracts were freeze-dried, and a solution reconstituted from the dried extract was filtered through a 0.22 μm membrane filter [33]. Compounds **93** and **94** were extracted with 70% ethanol under reflux, followed by separation through TLC and silica gel column chromatography [38]. Compound **95** was extracted with 70% methanol at a 1:40 (*w*/*v*) ratio by repeating the process three times for 20 min each [39].

### 7.3. Terpenoids

To date, 11 terpenoid compounds have been isolated and identified from *P. amurense* (Table 3). The extraction and separation methods for terpenoids from *P. amurense* can be summarized as follows. Compounds **99** and **100** were obtained by triple reflux extraction of chopped raw materials with 80% methanol, and the collected extract was then subjected to separation via silica gel column chromatography [29]. Compound **101** was extracted with methanol, followed by sequential partitioning with *n*-hexane, chloroform, and ethanol; the resulting fractions were then purified by silica gel column chromatography and recrystallization [36]. Compounds **102**–**104** were extracted with methanol under reflux; the methanol extract was then suspended in water and extracted with ethyl acetate, followed by separation of the ethyl acetate extract via silica gel column chromatography and HPLC [32]. Compound **105** was extracted with 70% ethanol under reflux, followed by separation via TLC and silica gel column chromatography [38]. Compound **106** was first defatted with *n*-hexane, after which reflux extraction was carried out successively with acetone and methanol [40]. Compound **107** was obtained by grinding, sieving, and drying the raw material, followed by ultrasonic extraction with 75% ethanol, dissolution in methanol, and filtration [30]. Compounds **108** and **109** were extracted with methanol under reflux and then separated by silica gel column chromatography [41].

### 7.4. Steroids

Researchers have isolated and identified 5 steroids from *P. amurense* (Table 4). The extraction and separation methods for steroids from *P. amurense* can be summarized as follows. Compounds **110** and **111** were extracted with 70% ethanol under reflux, followed by separation via TLC and silica gel column chromatography [38]. Compounds **112** and **113** were obtained by cold maceration with ethanol under shaking; after treatment with benzene and vacuum concentration, the residue was dissolved in an ethanol solution and subjected to phosphorylation by boiling, and the target compound was finally separated via recrystallization [42]. Compound **114** was first adsorbed onto an alumina column (20:1 ratio). Fluorides were then removed with a petroleum solvent and precipitated, and the precipitate was collected using benzene. After several cycles of this process, final separation was achieved through a secondary adsorption treatment on a larger alumina column (30:1 ratio) [43].

### 7.5. Lignans

To date, researchers have isolated and identified 4 lignans from *P. amurense* (Table 5). The extraction and separation methods for lignans from *P. amurense* can be summarized as follows. Compounds **115** and **118** were extracted using methanol; after the combined extracts were concentrated under reduced pressure, the obtained syrup was suspended in water and then extracted with ethyl acetate, followed by separation of the ethyl acetate fraction via silica gel column chromatography [35]. Compounds **116** and **117** were obtained by methanol extraction, and the extract was further separated by chromatographic methods including TLC and silica gel column chromatography [37].

### 7.6. Flavonoids

To date, researchers have isolated and identified 7 flavonoids from *P. amurense* (Table 6). The extraction and separation methods for flavonoids from *P. amurense* can be summarized as follows. Compounds **119**–**121** were extracted by reflux with 75% ethanol [44]. Compounds **122**, **124**, and **125** were extracted with methanol under reflux and then separated by silica gel column chromatography [41]. Compound **123** was extracted with methanol and subsequently partitioned with diethyl ether, and the organic layer was then separated by silica gel column chromatography [45].

### 7.7. Volatile Compounds

31 volatile compounds have been isolated and identified from *P. amurense* (Table 7). The extraction and separation methods for volatile compounds from *P. amurense* can be summarized as follows. Compounds **126**–**155** were obtained by drying and crushing the sample, followed by extraction and subsequent GC-MS separation [46]. Compound **156** was obtained by triple ethanol reflux extraction with 50% ethanol, followed by rotary evaporation concentration, freeze-drying, and pulverization; the sample was then dissolved in 50% methanol by ultrasonication and filtered [28].

### 7.8. Other Compounds

Researchers have isolated and identified 14 additional compounds from *P. amurense* (Table 8). The extraction and separation methods for these compounds can be summarized as follows. Compounds **157**–**165** were extracted with 1.2 L of double-distilled water at 100 °C for 3 h and then filtered through No.1 filter paper; the filtrate was concentrated using a rotary evaporator and stored at 4 °C prior to analysis [47]. Compound **166** was obtained by triple ethanol reflux extraction with 50% ethanol, followed by rotary evaporation concentration, freeze-drying, and pulverization; the sample was then dissolved in 50% methanol by ultrasonication and filtered [28]. Compound **167** was subjected to triple reflux extraction with 75% methanol, after which the combined extracts were freeze-dried, and a solution reconstituted from the dried extract was filtered through a 0.22 μm membrane filter [33]. Compounds **168**–**170** were extracted using methanol; after the combined extracts were concentrated under reduced pressure, the obtained syrup was suspended in water and then extracted with ethyl acetate, followed by separation of the ethyl acetate fraction via silica gel column chromatography [35].

## 8. Pharmacology

**Figure 3:**
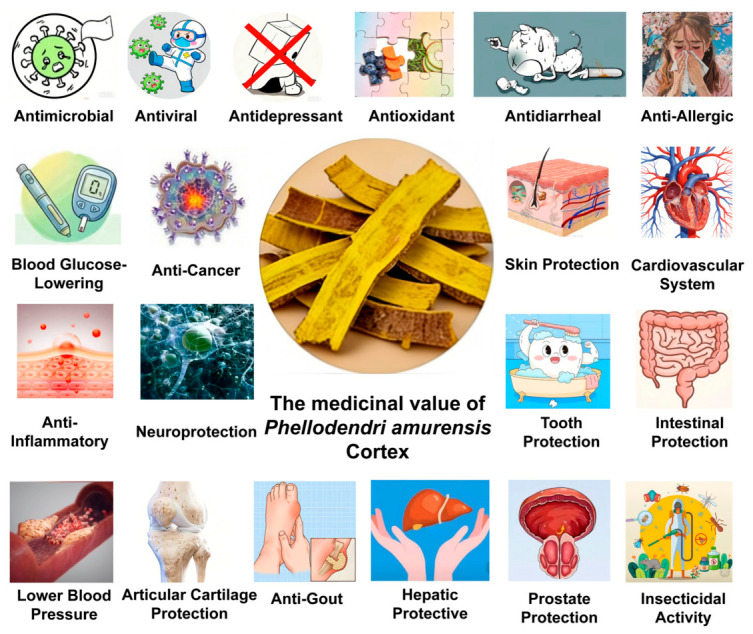
The medicinal value of *Phellodendri amurensis* Cortex.

The medicinal applications of PAC are supported by modern pharmacological evidence, which demonstrates its multiple beneficial actions, including antioxidant, hypoglycemic, antihypertensive, anti-inflammatory, antipyretic, anticancer, immunosuppressive, ulcer-protective, anti-gout, and antiviral activities (Figure 3). In TCM, it is widely regarded as a superior herb among heat-clearing and dampness-drying medicines, known for its significant efficacy in clearing heat, drying dampness, purging fire, relieving steaming sensations, detoxifying, and healing sores. Before presenting the pharmacological evidence, it is important to clarify that only studies specifically using PAC as the experimental material are included in this section. Given the historical conflation of PAC and PCC under the single name “Huangbai”, we have critically evaluated each cited reference to confirm the botanical origin of the plant material. Studies that did not provide clear species identification or that used *P. chinense* exclusively were excluded. This rigorous approach ensures that the pharmacological activities described below—including antioxidant, anti-inflammatory, anticancer, and other effects—are reliably attributed to PAC.

### 8.1. Antioxidant Effects

The natural antioxidant effects of Chinese herbal medicines (such as anti-aging, wrinkle reduction, and spot fading) primarily rely on components such as flavonoids and esters. These compounds can bind to oxygen free radicals or block their transport pathways, thereby exerting antioxidant effects. In vitro, Zhang and colleagues [48] observed that the total flavonoid extract from *P. amurense* exhibits significant scavenging ability against hydroxyl radicals, superoxide anion radicals, and 2,2-diphenyl-1-picrylhydrazyl (DPPH) radicals, with half-maximal inhibitory concentration (IC_50_) values calculated as 2.9525 mg/L, 1.6827 mg/L, and 1.3951 mg/L, respectively, suggesting that the total flavonoid extract from *P. amurense* has a strong DPPH radical scavenging effect. Yan and colleagues [49] demonstrated through in vitro experiments that PAC polysaccharides can reduce stable DPPH radicals to yellow diphenylpicrylhydrazine, while in vivo experiments showed that they also reduce ultraviolet (UV)-induced lipid peroxidation in rat skin, enhance antioxidant enzyme activity and skin immune activity, and alleviate oxidative damage. This study also observed enhanced immune function by increasing IL-10 and TNF-*α* levels, thereby effectively alleviating ultraviolet-induced skin oxidative stress. These findings highlight the potential value of PAC in photoprotective skin applications. Furthermore, gas chromatography–mass spectrometry analysis of the aqueous extract of PAC identified nine compounds with antioxidant capacity, with a total polyphenol content of 70 μg/mg. Its antioxidant activity showed a strong correlation with polyphenol content, highlighting its potential as a natural preservative. HPLC analysis confirmed that PAC polysaccharides contain sugar components such as arabinose and xylose. Their Fourier transform infrared spectroscopy spectra displayed characteristic absorption peaks typical of carbohydrate polymers. In vitro experiments verified that these polysaccharides possess significant dose-dependent free radical scavenging activity. In an in vivo rat model, it was observed that UV irradiation led to a decline in both antioxidant enzyme activity and immune parameters in the skin. This effect was mitigated by oral administration of PAC polysaccharides, which resulted in a dose-dependent elevation of catalase and glutathione peroxidase activity [47].

### 8.2. Therapeutic and Blood Glucose-Lowering Effects on Diabetes

This study employed a bioactivity-guided isolation approach to identify oleic acid as the primary active component from PAC. This compound specifically inhibits protein tyrosine phosphatase 1B (PTP1B), and the associated molecular mechanism underlying its anti-type 2 diabetes activity is suggested (PTP1B is characterized as a pivotal negative regulator of insulin and leptin signal transduction; inhibiting its activity can improve insulin resistance).

In vitro experimental results showed that the dichloromethane extract of PAC exhibited significant PTP1B inhibitory activity (residual enzyme activity was only 34% at 10 μg/mL), and this activity was not attributed to alkaloids such as berberine or palmatine present in the extract (the alkaloid fraction showed no PTP1B inhibition). Following purification via silica gel column chromatography and reversed-phase preparative chromatography, the active component oleic acid was obtained. Its in vitro inhibition of PTP1B was dose-dependent, with an IC_50_ value of 6.2 μM. Oleic acid exhibited only mild inhibition (IC_50_ = 5.3 μM) against T lymphocyte protein tyrosine phosphatase, which has high homology, and showed no significant effect on other phosphatases such as Src homology 2 domain-containing phosphatase 2 and leukocyte common antigen-related, indicating a degree of selectivity for PTP1B.

Cellular experiments further verified that, in PTP1B-reconstituted mouse embryonic fibroblasts, oleic acid dose-dependently enhanced insulin-mediated AKT phosphorylation. This effect was absent in PTP1B knockout cells, confirming that its insulin-sensitizing action depends on the modulation of PTP1B. Subsequent testing of the inhibitory activity of various fatty acids revealed that all tested fatty acids exhibited PTP1B inhibitory activity at the micromolar level, with longer-chain saturated fatty acids showing stronger activity (e.g., arachidic acid IC_50_ = 1.6 μM, eicosanoic acid IC_50_ = 1.4 μM). In summary, oleic acid from PAC improves insulin signaling by specifically inhibiting PTP1B activity, suggesting that it serves as an important material basis for the anti-diabetic effects of this medicinal herb. Furthermore, the widespread PTP1B inhibitory activity of fatty acid components offers a novel framework for understanding the feedback regulation mechanisms of metabolic disorders [50].

Kim and colleagues [51] investigated the effects of *P. amurense* extract on hyperglycemia and diabetic nephropathy in streptozotocin-induced diabetic rats. In vivo, male Sprague-Dawley rats were divided into a normal control group (NC), a diabetic model group (DC), and a diabetic group treated with PAC extract (DP). During a 4-week experimental period, rats in the DP group were administered PAC extract by gavage at a dose of 379 mg/kg body weight/d. The results showed that final fasting blood glucose, urinary total protein levels, and relative left kidney weight in the DP group were significantly lower than those in the DC group. Renal xanthine oxidase (XO) and superoxide dismutase (SOD) activities in the DP group were significantly lower than those in the DC group, while catalase activity was significantly higher. Compared with the DC group, the degree of renal tubular epithelial injury was markedly reduced in the DP group. Chinese patent medicines containing PAC (such as Tongguan Pills and Zhibai Dihuang Pills) have also been used in the therapy of diabetes and its complications [52].

In vitro, human hepatoma G2 (HepG2) cells were cultured and treated with different concentrations of berberine, along with a biotin group as a positive control, for 24 h. Subsequently, cell proliferation ability, glucokinase activity and its messenger RNA (mRNA) expression, as well as glycogen content, were measured. The results showed that berberine at concentrations below 5 μM had no significant effect on HepG2 cell proliferation, while at concentrations above 15 μM, the inhibitory effect on cell proliferation increased with increasing concentration. Glucokinase activity increased with higher berberine concentrations, and within a certain range, both glycogen content and glucokinase mRNA expression in HepG2 cells were positively correlated with berberine concentration. These findings suggest that berberine can lower blood glucose by increasing glucokinase activity and glucokinase mRNA expression in HepG2 cells [53].

This study investigated the ameliorative effects of berberine on hyperglycemia and hyperlipidemia in diabetic hamsters induced by a high-fat and high-sugar diet. In vivo, hamsters fed a high-fat and high-sugar diet were treated with metformin, simvastatin, or low- and high-dose berberine (50, 100 mg/kg) for 6 weeks. The results showed that body weight in the berberine-treated groups was significantly lower than that in the control group. Histological analysis indicated that berberine inhibited hepatic fat accumulation. Berberine significantly reduced plasma levels of total cholesterol, triglycerides, free fatty acids, low-density lipoprotein cholesterol, malondialdehyde, thiobarbituric acid-reactive substances, and 8-isoprostane, while significantly increasing plasma SOD activity. Both blood glucose and insulin levels were significantly reduced in the metformin and berberine groups. Glucose tolerance test results confirmed that animals treated with berberine exhibited stronger glucose tolerance. Berberine upregulated glucose transporter 4 mRNA expression in skeletal muscle and significantly downregulated low-density lipoprotein receptor mRNA expression in the liver. These findings suggest that berberine effectively lowers blood glucose and lipid levels, reduces body weight, and alleviates oxidative stress in diabetic hamsters, which may help reduce the risk of diabetes-related cardiovascular diseases [54].

Zhang and colleagues administered different doses (5 mg/mL, 10 mg/mL) of limonin and nomilin via gavage to high-fat diet-fed mice. In vivo, both compounds significantly reduced serum total cholesterol, triglycerides, and low-density lipoprotein cholesterol, while increasing high-density lipoprotein cholesterol levels; high-dose nomilin increased high-density lipoprotein cholesterol by 71.95%. Additionally, both compounds ameliorated colonic adipose tissue pathology, increased goblet cell numbers, and optimized gut microbiota composition by increasing beneficial bacteria and decreasing harmful bacteria. Metabolomics revealed that limonin and nomilin remodeled systemic metabolism, particularly lipid metabolism and steroid hormone biosynthesis, with nomilin showing superior lipid-regulating effects by specifically upregulating steroid hormone biosynthesis-related metabolites. This study highlights the potential value of these limonoids in improving lipid metabolic homeostasis for functional food and health supplement applications [55].

### 8.3. Antimicrobial Effect

Liang [56] prepared test bacterial strains and compared them with different concentrations of PAC extract to determine the minimum inhibitory concentration (MIC) of PAC, and found that it exhibits significant inhibitory effects against *Streptococcus pneumoniae*, *Bacillus subtilis*, and *Pseudomonas aeruginosa*, with the strongest antibacterial activity observed against *Staphylococcus aureus* and *Staphylococcus epidermidis*. Further research by Chen [57] confirmed that PAC has potent inhibitory effects on respiratory system bacteria and ribonucleic acid synthesis, with its mechanism linked to the inhibition of *Staphylococcus aureus*, *Proteus* spp., as well as Group A and B streptococci. Another study discovered that the bark extract of PAC has an MIC range of 9.8–312.5 μg/mL against the oral pathogen *Streptococcus mutans*. It effectively inhibits the growth, biofilm formation, and acid production of *Streptococcus mutans*, while showing no significant toxicity to oral epithelial cells, emphasizing its potential utility in oral care [58].

In terms of antifungal activity, berberine hydrochloride and palmatine hydrochloride isolated from PAC exhibit significant activity against *Microsporum canis*. Their combined effect is superior to that of either compound alone or the clinically common drug clotrimazole, suggesting a potential natural therapeutic option for fungal skin infections. In vitro experiments demonstrated that the MIC of both berberine hydrochloride and palmatine hydrochloride against *Microsporum canis* is 1 mg/mL, which is higher than that of clotrimazole (0.015 mg/mL). However, both compounds significantly inhibited fungal growth after 18 h. At 30 h, the combination treatment group showed a significantly stronger upregulation of genes related to fungal energy metabolism and virulence (such as plasmid with *GAL4* promoter (*PGAL4*), family of serine hydrolases 1 (*FSH1*), and PQ-loop repeat protein (*PQ-LRP*)) compared to the single-compound groups and the clotrimazole group (*p* < 0.05). Transmission electron microscopy revealed that both alkaloids disrupt the integrity of the fungal cell membrane and organelles, interfering with normal fungal growth. Mechanistically, palmatine induces early (6 h) upregulation of related gene expression, while berberine maintains a high level of gene expression, thereby shortening the fungal lifecycle. The two compounds work synergistically to enhance antifungal efficacy.

In vivo, a rabbit model of skin infection with *Microsporum canis* was established. The combination treatment group with berberine hydrochloride and palmatine hydrochloride showed the fastest recovery of skin lesions, with significantly lower clinical scores on day 17 compared to the single-compound groups and the clotrimazole group. Periodic acid-Schiff staining of skin tissue indicated the least residual fungal presence in the combination treatment group, and no notable toxicity was observed throughout the treatment period. In summary, berberine hydrochloride and palmatine hydrochloride exert antifungal effects against *Microsporum canis* by disrupting fungal cellular structures and modulating the expression of key genes. Their combined use exhibits synergistic effects, demonstrating potential for development into natural topical antifungal formulations [59].

### 8.4. Anti-Inflammatory Effect

The anti-inflammatory effects of PAC arise from the multi-target regulation of complex inflammatory pathways by its various active components.

This study aimed to investigate the anti-inflammatory activity of *P. amurense* both in vivo and in vitro. In vivo, mice were pretreated with *P. amurense* (200 mg/kg) by gavage for three consecutive days. 2 h after the final administration, an endotoxemia model was established by intraperitoneal injection of lipopolysaccharide (LPS, 35 mg/kg). The results showed that *P. amurense* significantly increased the survival rate of mice and downregulated LPS-induced serum levels of IL-6, IL-1*β*, and monocyte chemoattractant protein-1 (MCP)-1. Furthermore, *P. amurense* inhibited the expression of inducible nitric oxide synthase (iNOS), blocked nuclear factor-kappa B (NF-*κ*B) activation by inhibiting the degradation and phosphorylation of I*κ*B*α*, and attenuated the phosphorylation of (MAPKs, including extracellular signal-regulated kinase (ERK)1/2, p38, and JNK) in LPS-stimulated mice. In vitro, in LPS-induced Raw Abelson Wistar (RAW) 264.7 macrophages, *P. amurense* dose-dependently downregulated LPS-induced nitric oxide (NO), iNOS expression, and the expression of inflammatory cytokines and related proteins, which were consistent with the in vivo results [60].

In a study on promoting tissue regeneration and healing in healthy guinea pigs, Zhao [61] found that PAC exhibits significant anti-inflammatory effects on skin lesions infected with *Staphylococcus aureus*. Additionally, while exerting antipyretic and anti-inflammatory effects, PAC also promotes angiogenesis, improves wound microcirculation, accelerates granulation tissue growth, and enhances wound healing [62].

Through methanol extraction, liquid–liquid extraction, and silica gel column chromatography, a bioalkaloid-rich fraction A was obtained from the bark of PAC. Gas chromatography–electron ionization–mass spectrometry analysis revealed that its main components are phellodendrine and magnoflorine. In vitro activity tests showed that this fraction exhibits dose-dependent inhibition of type II phospholipase A_2_ (PLA_2_): at 2.5 μg/mL, the inhibition rate was 30% (*p* = 0.03); at 10 μg/mL, 46% (*p* = 0.01); and at 25 μg/mL, 63% (*p* = 0.01). In comparison, the positive control manoalide achieved a 76% inhibition rate at 1 μg/mL. Since PLA_2_ is a key enzyme in the release of arachidonic acid and the generation of inflammatory mediators (e.g., leukotrienes and prostaglandins) during inflammation, the inhibitory activity of the alkaloid fraction from PAC against PLA_2_ suggests its potential to exert anti-inflammatory effects by blocking inflammatory pathways. This is particularly suitable for topical applications in treating skin erythema or sensitive conditions [63].

By establishing a monitoring system for smooth muscle cells and keratinocytes, it was found that PAC extract and its active component 4-*O*-feruloylquinic acid can effectively suppress particulate matter 2.5-induced inflammatory responses (modeled using diesel particulate matter (DPM)) in human keratinocytes by regulating the protease-activated receptor-2 (PAR-2) signaling pathway. DPM activates PAR-2, mediating transient Ca^2+^ influx, which subsequently upregulates the expression of pro-inflammatory factors such as IL-6, IL-8, and TNF-*α*, while reducing the expression of tight junction proteins zonula occludens-1 and occludin, thereby compromising skin barrier integrity. PAC extract concentration-dependently inhibited DPM-induced Ca^2+^ influx (with an inhibition rate exceeding 50%), downregulated PAR-2 mRNA and protein expression, reversed its abnormal activation, and significantly suppressed the increase in pro-inflammatory factors while restoring barrier protein levels. Its active component 4-*O*-feruloylquinic acid fully replicated the anti-inflammatory effects of PAC extract by regulating the PAR-2 signaling pathway through the same mechanism, blocking the inflammatory cascade triggered by DPM, and is identified as the key substance responsible for the anti-inflammatory effects of PAC extract [64].

Jiang and colleagues [65] demonstrated that palmatine ameliorates monosodium urate (MSU)-induced acute gouty inflammation by inhibiting NOD-like receptor thermal protein domain associated protein 3 (NLRP3) inflammasome-mediated pyroptosis. In vitro experiments showed that palmatine treatment dose-dependently reduced the expression of pyroptosis-related proteins including NLRP3, Apoptosis-associated speck-like protein containing a CARD (ASC), cysteinyl aspartate specific proteinase (caspase)-1), IL-1*β*, high mobility group box 1, and cathepsin B, while also downregulating neutrophil extracellular trap-related proteins such as caspase-11, histone H3, proteinase 3, and peptidylarginine deiminase 4. In vivo experiments established two acute gout models: An acute gouty arthritis model induced by injecting MSU crystals into the mouse foot pad, and an air pouch acute gout model induced by subcutaneously injecting MSU crystals into the mouse back. These experiments revealed that palmatine not only alleviated the inflammatory response in gouty arthritis but also exerted chondroprotective effects by upregulating matrix metalloproteinase (MMP)-3. This study indicates that palmatine inhibits pyroptosis by simultaneously blocking both the canonical and non-canonical pathways of the NLRP3 inflammasome and interfering with neutrophil extracellular traps formation.

Hao and colleagues [66] analyzed the metabolites in normal rats following oral administration of *P. amurense* decoction using ultra-performance liquid chromatography–quadrupole time-of-flight–mass spectrometry, identifying 72 exogenous components (17 prototype compounds and 55 metabolites) and 14 biomarkers. In vivo, in an adjuvant-induced arthritis rat model, *P. amurense* significantly improved rheumatoid arthritis symptoms, with its components undergoing primarily phase I (oxidation, reduction, dehydrogenation, demethylation) and phase II metabolism (acetylation, glucuronidation, methylation). The mechanism of action involves pathways related to aminoacyl-tRNA biosynthesis, phenylalanine metabolism, tryptophan metabolism, valine/leucine/isoleucine degradation, and pantothenate and coenzyme A biosynthesis.

Liu and colleagues [67] demonstrated that obakulactone isolated from PAC treats rheumatoid arthritis by targeting acyl-coenzyme A thioesterase 1: In vivo, obakulactone significantly alleviated joint swelling in rats, regulated immune balance by promoting macrophage polarization from macrophage 1 to macrophage 2 and inhibiting T helper 17 cell differentiation, and restored serum levels of inflammatory factors and diagnostic markers. Mechanistically, obakulactone promotes ubiquitin-mediated degradation of acyl-coenzyme A thioesterase 1, downregulates stearoyl-CoA desaturase 1 expression, and subsequently inhibits the janus kinase (JAK)/signal transducer and activator of transcription and phosphatidylinositol 3-kinase/protein kinase B (PI3K/AKT) pathways, reducing inflammation and fibrosis in synovial fibroblasts. Feng and colleagues [68] showed that berberine improves glucose and lipid metabolism and alleviates hypothalamic inflammation in a high-fructose and high-fat diet-induced mouse model by inhibiting MAPK/NF-*κ*B pathway phosphorylation and promoting microglial polarization from macrophage 1 to macrophage 2, with in vitro experiments confirming its neuroprotective effects. Together, these studies reveal the molecular mechanisms by which active compounds from *Phellodendron* ameliorate inflammatory diseases through regulating macrophage/microglial polarization and multiple signaling pathways.

### 8.5. Effects on the Nervous System and Memory Impairment

Through cell experiments, it was found that PAC exerts neuroprotective effects against 1-methyl-4-phenylpyridinium-induced apoptosis in pheochromocytoma cell line 12 (PC12) cells. This extract inhibits the increase in the Bcl-2-associated X protein (Bax)/B-cell lymphoma 2 (Bcl-2) ratio, reduces cytochrome c release, suppresses cysteinyl aspartate-specific proteinase-3 (caspase-3) activation and poly(ADP-ribose) polymerase 1 (PARP1) cleavage, thereby significantly ameliorating 1-methyl-4-phenylpyridinium-induced cytotoxicity, suggesting its potential protective role in neurodegenerative diseases [69].

Lee and colleagues [70] explored whether PAC and its key alkaloid berberine could attenuate memory impairment induced by scopolamine in a rat model. In vivo, the study found that both PAC and berberine significantly mitigated the decline in cholinergic immunoreactivity related to memory and restored hippocampal mRNA levels of brain-derived neurotrophic factor and cyclic adenosine monophosphate response element-binding protein, indicating their modulatory effects on the cholinergic system and neurotrophic factor pathways.

In a study on an Alzheimer’s disease model, a neurotoxicity model was established in PC12 cells. The ethanol extract of PAC demonstrated protective effects against *β*-amyloid (A*β*_25–35_)-induced neurotoxicity in PC12 cells. This extract contains 4 common chemical markers, including berberine. Experiments revealed that A*β*_25–35_ treatment led to decreased cell viability, increased DNA fragmentation, elevated cytochrome c release, downregulation of the Bcl-2/Bax ratio (both protein and mRNA levels), and upregulation of caspase-3 expression (both protein and mRNA levels), thereby inducing apoptosis. In contrast, pretreatment with the PAC extract dose-dependently reversed these changes, significantly enhancing cell viability and suppressing abnormalities in apoptosis-related indicators [71].

Research further suggests that components in PAC, such as berberine and magnoflorine, hold potential for alleviating neuropsychiatric disorders like anxiety and depression. In an APP/PS1 transgenic mouse model of Alzheimer’s disease, berberine was observed to promote neuroregeneration via the IGFR-mediated JAK/AKT signaling pathway [72] and exhibits anti-Alzheimer’s disease activity: It inhibits acetylcholinesterase (IC_50_ = 1.85 mM), reduces *β*-amyloid deposition, suppresses tau protein hyperphosphorylation, and exerts neuroprotective effects through antioxidant (lowering malondialdehyde and reactive oxygen species levels) and anti-inflammatory (inhibiting NF-*κ*B and MAPK pathways) actions [73].

Hu and colleagues [74] aimed to investigate the therapeutic effect of phellodendrine on major depressive disorder through network pharmacology and experimental validation. Multiple databases were used to predict the targets of phellodendrine and depression, and the intersecting targets were identified as potential targets for the antidepressant effect of phellodendrine. A protein–protein interaction network was constructed, followed by Gene Ontology and Kyoto Encyclopedia of Genes and Genomes pathway analyses. Molecular docking was further performed to predict binding interactions. A total of 38 intersecting targets between phellodendrine and depression were identified. These in silico findings suggest that phellodendrine may exert its antidepressant effect by regulating targets such as Solute carrier family 6 member (*SLC6A*)*4*, *SLC6A3*, *SLC6A2*, and Monoamine Oxidase A through serotoninergic synapses, salivary secretion, dopaminergic synapses, and the cyclic adenosine monophosphate (cAMP) signaling pathway. In vitro experiments using PC12 cells showed that, compared with the corticosterone (CORT) group, phellodendrine increased mitochondrial DNA copy number. Different concentrations of phellodendrine affected IL-6 and IL-1*β* levels and regulated the mRNA expression of key targets in the Cholinergic Receptor, Muscarinic1, 5-Hydroxytryptamine Receptor 1A, and PI3K/AKT pathways [74].

Syringin ameliorates behavioral deficits in animal models of Alzheimer’s disease (AD), but whether it suppresses the NLRP3 inflammasome via the SWELL1 channel remains unclear. Xu and colleagues [75] found that syringin shortened the escape latency in the Morris water maze test in APP/PS1 mice, reduced inflammation-related mRNA levels, and downregulated the protein expression of NLRP3, ASC, pro-caspase-1, and Gasdermin D *N*-terminal domain. In vitro, in BV2 microglial cells, syringin inhibited the release of cleaved caspase-1 and IL-1*β*, reduced Iba-1 expression, decreased the transcription of microglial activation markers, suppressed lactate dehydrogenase release, and reduced Gasdermin D N-terminal domain expression. Conditioned medium from syringin-treated BV2 cells increased the survival rate of HT22 neurons and decreased cleaved caspase-3 levels. Mechanistically, syringin disrupted the interaction between ASC and pro-caspase-1 (without affecting the ASC-NLRP3 interaction), reduced ASC monomer/dimer formation, suppressed ROS and restored mitochondrial membrane potential, while inhibiting SWELL1 channel currents. Molecular dynamics simulations predicted that syringin binds to the critical residues Tyr99 and Asp100. Collectively, syringin may inhibit NLRP3 inflammasome activation by suppressing the microglial SWELL1 channel, thereby alleviating AD-related neuroinflammation.

### 8.6. Anti-Cancer and Apoptosis-Inducing Effects

Bioactive components in PAC such as magnoflorine, palmatine, berberine, and their extracts (e.g., Nexrutine) exert broad anticancer effects through multi-pathway regulation and cellular function intervention, capable of inducing apoptosis in cancer cells and are applicable to various cancer types. HPLC analysis revealed that berberine from dietary supplementation was detectable in the lungs of mice fed with a dose of berberine (518 ng/mL). Furthermore, in vivo, berberine inhibited the growth of xenografted A549 cell tumors (reaching 9.4 mm^3^ and 6.4 mm^3^, respectively), whereas the tumor volume in the control group was 58.9 mm^3^ (*p* < 0.001). Studies have shown that dietary supplementation with berberine or PAC extract can suppress the growth of lung cancer cells and slow tumor growth in vivo, significantly reducing lung tumor volume; this effect achieves suitable concentrations in lung tissue without apparent toxicity [76].

A single dose of Nexrutine (1.0 mg/mouse) given prior to 12-*O*-tetradecanoylphorbol-13-acetate (TPA) significantly inhibited TPA-induced skin edema, hyperplasia, thymidine incorporation, and ornithine decarboxylase activity; suppressed cyclooxygenase-2 (COX-2) and iNOS expression; inhibited phosphorylation of MAPKs (ERK1/2, p38, JNK); and blocked IκB kinase, Inhibitor of κB alpha, and NF-*κ*B activation in mouse skin. In vivo, in a two-stage skin tumorigenesis model, twice-weekly Nexrutine treatment before TPA promotion reduced tumor incidence and burden, and prolonged tumor latency [77]. In vitro, in gastric cancer cells (SGC-7901, MGC-803), Nexrutine inhibited cell viability and invasion, downregulated Proliferating Cell Nuclear Antigen, CyclinD1, and Bcl-2, promoted apoptosis and Bax expression, and reduced levels of STAT3, p-STAT3, NF-*κ*B p65, p-p65, FAK, and p-FAK. Focal Adhesion Kinase (FAK) overexpression reversed these effects. In vivo, Nexrutine (600 mg/kg in diet for 21 d) reduced tumor volume and weight, and decreased FAK, STAT3, and NF-*κ*B p65 expression and phosphorylation in tumor tissues [78].

Palmatine, on the other hand, inhibits cancer cell proliferation by downregulating phosphorylated ribosomal protein S6, suppressing NF-*κ*B transcriptional activity and its downstream target gene FLICE-inhibitory protein, and blocking glutamate-mediated glioma-associated oncogene signaling transduction. For example, in vitro in prostate cancer cells (such as DU145 and prostate cancer 3), palmatine can inhibit their proliferation, invasion, and anchorage-independent growth, with similar inhibitory effects observed in androgen-independent cells [79].

Zhang and colleagues [80] evaluated the effects of phellamurin on cell viability and apoptosis using the MTT assay and flow cytometry, respectively. Protein levels of phosphorylated phosphatidylinositol 3-kinase, phosphorylated protein kinase B (p-AKT), AKT, phosphorylated mammalian target of rapamycin (p-mTOR), and mTOR were detected by Western blot. In vitro, the results showed that phellamurin dose-dependently inhibited osteosarcoma cell viability and promoted apoptosis. Notably, phellamurin inhibited the PI3K/AKT/mTOR pathway in osteosarcoma cells. LY294002 effectively suppressed the PI3K/AKT/mTOR signaling pathway, reduced osteosarcoma cell viability, and induced apoptosis, whereas activation of this pathway with 740Y-P reversed the effects of phellamurin on osteosarcoma cell viability and apoptosis. In vivo experiments showed that phellamurin inhibited osteosarcoma growth and suppressed the PI3K/AKT/mTOR pathway in tumor tissues. In conclusion, phellamurin inhibits osteosarcoma cell viability and induces apoptosis, at least partially through the suppression of the PI3K/AKT/mTOR pathway. This study suggests that phellamurin has the potential to become a novel chemotherapeutic agent for the treatment of osteosarcoma.

To rapidly screen COX-2 inhibitors in PAC extracts, ultrafiltration-liquid chromatography/mass spectrometry was employed, preliminarily identifying 13 potential active peaks. 11 of these were confirmed as COX-2 inhibitors, including alkaloids such as tetrahydropalmatine, palmatine, and berberine, as well as compounds like limonin. For the large-scale preparation of these active components, an online method combining pressurized liquid extraction (PLE), countercurrent chromatography (CCC), and semi-preparative liquid chromatography (semi-PLC) was established. Separation conditions were optimized based on component polarity: alkaloids (e.g., tetrahydropalmatine, jatrorrhizine) were isolated via CCC, while other components were purified by semi-PLC, ultimately yielding eleven high-purity compounds (all > 93.4% purity). In vitro enzyme inhibition assays further verified that palmatine, berberine, and jatrorrhizine exhibited the most significant COX-2 inhibitory activities (inhibition rates of 61.22%, 60.53%, and 56.55%, respectively, at 15 μg/mL), consistent with the ultrafiltration screening results. Their activities were superior to that of the crude PAC extract (inhibition rate 53.22%). In summary, alkaloids in PAC are the primary COX-2 inhibitors with potential anticancer applications. The online PLE/CCC/semi-PLC technology is efficient and automated, providing a feasible technical solution for the efficient screening and bulk production of active constituents derived from natural sources [81].

Research by Qiu and colleagues [82] demonstrated that oral administration of a decoction of PAC significantly inhibits immunoglobulin M (IgM) production in mice with delayed-type hypersensitivity (DTH) induced by sheep red blood cells (SRBCs), reduces splenocyte proliferation, and decreases the generation of serum lysozyme. The primary immunomodulatory components of PAC-magnoflorine, phellodendrine, and berberine-were found to suppress both the local graft-versus-host (GVH) reaction in mice and the induction phase of picryl chloride-induced DTH, with no significant effect on the effector phase. Among these, in a mouse model, after injection of phellodendrine, it was found that phellodendrine exhibited more pronounced activity in inhibiting GVH reactions and cellular immunity [83]. These components collectively modulate delayed-type hypersensitivity immune responses by blocking local GVH reactions and the afferent phase of DTH [84]. Moreover, through cell experiments, it was found that total polysaccharides from PAC significantly inhibit the in vitro proliferation of mouse splenic lymphocytes induced by concanavalin A, demonstrating immunosuppressive activity [85].

Further studies by Li [86] confirmed that phellodendrine and magnoflorine can suppress the local GVH reaction in mice, prolong survival time, and improve survival rates. Research by Lv [87,88] revealed that berberine inhibits DTH induced by dinitrofluorobenzene in mice, reduces serum levels of interferon-gamma (IFN-*γ*), and attenuates the release of related inflammatory factors. Its mechanism of action may involve suppressing the production and secretion of cytokines such as IFN-*γ* and IL-1, thereby inhibiting immune responses and mitigating inflammatory damage. Mori and colleagues [89] conducted cell experiments and found that phellodendrine significantly inhibits both local and systemic GVH reactions, as well as the induction phase of DTH triggered by SRBCs and guinea pig tuberculin, without affecting the effector phase of DTH or antibody production. Additionally, it does not suppress humoral immunity, suggesting that phellodendrine represents a novel immunosuppressant capable of selectively inhibiting cellular immune responses.

Lin and colleagues [90] established cell experiments and found that oxypalmatine inhibits breast cancer cell proliferation and DNA replication and induces apoptosis by inactivating the PI3K/AKT signaling pathway. It also exhibits cytotoxicity against Luminal A, human epidermal growth factor receptor 2-overexpressing, and triple-negative breast cancer organoids, marking the first confirmation of its efficacy in breast cancer organoid models. Qiao and colleagues [91] evaluated the efficacy of oligopeptide transporter (OPT) using 4 lung adenocarcinoma cell lines and 4 patient-derived organoid tissues. demonstrated that OPT induces apoptosis in lung adenocarcinoma cells by targeting the PI3K/AKT pathway, while simultaneously triggering protective autophagy. Combining OPT with the autophagy inhibitor chloroquine enhances its pro-apoptotic effect, suggesting that this combination strategy may serve as a more effective therapeutic approach for lung cancer. Together, these studies reveal the anti-tumor mechanisms of oxypalmatine and its derivatives in breast and lung cancers through modulation of the PI3K/AKT pathway.

Tian and colleagues [92] demonstrated for the first time that berberine exerts protective effects against abdominal aortic aneurysm by preserving vascular structure and inhibiting inflammation: In vivo, in a porcine pancreatic elastase-induced mouse model, berberine suppressed aortic dilation, reduced inflammatory infiltration, maintained vascular smooth muscle cell survival, decreased elastic fiber degradation, and downregulated Matrix metalloproteinase-2/9 (MMP-2/9) expression and aberrant angiogenesis, with key target genes including runt-related transcription factor 2, vascular cell adhesion molecule 1, and chemokine (C-C motif) ligand 2. Li and colleagues [93] found that PAC inhibits prostate cancer through reprogramming lipid metabolism: In vivo, in CWR22 relapse variant 1 xenograft mice, *P. amurense* reversed 27 abnormal lipid biomarkers (primarily involved in glycerophospholipid, arachidonic acid, and sphingolipid metabolism), and its 9 active components (such as berberine and magnoflorine) targeted lipid-metabolizing enzymes including phosphatidylserine synthase 1, phospholipase A and acyltransferase 3, phosphatidylethanolamine *N*-methyltransferase, and phospholipase D family member 4, thereby inhibiting proliferation, inducing apoptosis, and remodeling the tumor microenvironment. Together, these studies reveal the therapeutic mechanisms of active compounds from *Phellodendron* in vascular and neoplastic diseases through the regulation of inflammation and lipid metabolism.

Hu and colleagues [94] investigated the mechanism of PAC and phellodendrine in the treatment of bladder cancer using network pharmacology, bioinformatics, and molecular docking. These in silico analyses predicted that phellodendrine exerts its anti-bladder cancer effects through 54 potential targets (such as JUN, IL6, and epidermal growth factor receptor) and 120 signaling pathways. For phellodendrine, 11 common targets were identified (including aurora kinase A, fatty acid synthase, and prostaglandin-endoperoxide synthase 1/2), with the highest binding affinity predicted for the Prostaglandin-endoperoxide synthase 2 protein (docking score: −7.183). Survival analysis revealed that high expression of JUN and epidermal growth factor receptor was associated with shortened overall survival in bladder cancer, while high expression of phosphatidylinositol-4,5-bisphosphate 3-kinase catalytic subunit alpha and ATP-binding cassette subfamily C member 1 correlated with poor disease-free survival. This study highlights the potential of phellodendrine in bladder cancer therapy through multi-target regulation.

Pan and colleagues [95] screened for PARP1 inhibitors from 507 chemical components of PAC, *Dictamnus dasycarpus*, and *Cnidium monnieri* through molecular simulation and bioactivity assays. Through molecular docking and enzyme activity experiments, 4 potential inhibitors were identified, among which demethyleneberberine exhibited the strongest PARP1 inhibitory activity (IC_50_ = 2.0 ± 0.8 μM), while the IC_50_ values of 8-hydroxydictamnine, meranzin hydrate, and osthole ranged from 44 to 76 μM. Molecular dynamics simulations revealed that non-polar interactions involving histidine 862, glycine 863, tyrosine 889, tyrosine 896, phenylalanine 897, and tyrosine 907 are the primary forces underlying inhibitor binding to PARP1. This study demonstrates that molecular simulation combined with activity assays is an effective approach for screening PARP1 inhibitors, and demethyleneberberine shows promising potential for further development.

Tumor-associated angiogenesis plays a key role in tumor growth and metastasis, leading to the approval of anti-angiogenic drugs such as sunitinib and axitinib. However, most target the vascular endothelial growth factor A (VEGFA)/vascular endothelial growth factor receptor 2 (VEGFR2) pathway and show variable efficacy due to resistance and increased invasiveness. Ng and colleagues [96] screened a natural product library using a zebrafish model and identified the indole alkaloid canthin-6-one, which significantly inhibited intersegmental vessel (ISV) and subintestinal vessel development. In vitro, canthin-6-one reduced ISV endothelial cell numbers and suppressed human umbilical vein endothelial cell (HUVEC) proliferation. Notably, it inhibited neither VEGFA-induced VEGFR2 phosphorylation in HUVECs nor downstream phosphorylation in zebrafish ISV endothelial cells, indicating VEGFA/VEGFR2-independent anti-angiogenic activity. In vivo, in a zebrafish B16F10 melanoma xenograft model, canthin-6-one inhibited tumor-associated angiogenesis and synergized with the VEGFR inhibitor sunitinib malate. Thus, canthin-6-one exhibits anti-angiogenic activity in both developmental and pathological settings independent of VEGFA/VEGFR2, warranting further development as a novel anti-angiogenic agent.

Non-small-cell lung cancer is a leading cause of cancer-related death worldwide, and patients often develop resistance to targeted therapies, necessitating new drugs. Lan and colleagues [97] investigated the natural alkaloid lotusine in non-epidermal growth factor receptor (EGFR)-mutant A549 cells and EGFR-mutant HCC827 cells (carrying an exon 19 deletion). In vitro, lotusine significantly inhibited HCC827 cell proliferation in a concentration- and time-dependent manner compared with A549 cells. Mechanistically, lotusine induced apoptosis in HCC827 cells by upregulating Bax and cleaved caspase-3 and downregulating Bcl-2, while arresting the cell cycle at G0/G1 phase. Western blot confirmed that lotusine reduced phosphorylated EGFR, phosphorylated AKT, and phosphorylated extracellular signal-regulated kinase (p-ERK), thus inhibiting the EGFR-AKT-ERK signaling pathway. In conclusion, these findings suggest that lotusine exerts anti-cancer effects through inhibiting proliferation, inducing apoptosis, and arresting the cell cycle, primarily via EGFR signaling inhibition, suggesting its potential as a candidate drug for EGFR-mutant non-small-cell lung cancer and laying a foundation for future preclinical and clinical studies.

Ning and colleagues [98] aimed to investigate the anticancer effects and mechanisms of oxyepiberberine on human colon cancer LS-1034 cells. In vitro, the results showed that oxyepiberberine exhibited potent anti-proliferative activity against LS-1034 cells, with an IC_50_ of 1.36 μM, and induced apoptosis and inhibited cell migration in a concentration-dependent manner. Importantly, this study demonstrated that oxyepiberberine is a potent inhibitor of tubulin polymerization, exerting concentration-dependent inhibitory effects with an IC_50_ value of 1.26 μM. In vivo, in a nude mouse xenograft model of colon cancer, oxyepiberberine suppressed tumor growth without obvious toxicity. In conclusion, as a novel tubulin polymerization inhibitor, oxyepiberberine holds promise as a candidate drug for the treatment of colon cancer.

### 8.7. Yin-Nourishing Effect

Qi [99] preliminarily explored the mechanism underlying the yin-nourishing effect of raw and salt-processed PAC from the perspectives of thyroid hormone levels and energy metabolism. In vivo, using rats as subjects, a hyperthyroidism-induced yin deficiency model was established by oral administration of high-dose thyroxine, with Liuwei Dihuang Pill serving as the positive control drug. Experimental results showed that regarding thyroid hormone levels, after oral administration of PAC extract, a significant decrease in serum free triiodothyronine and free thyroxine was detected in the model group, with the salt-processed group exhibiting a more substantial reduction than its raw counterpart. Additionally, heart rate was markedly lowered in both the positive control and raw groups. As for urinary 17-hydroxycorticosteroid levels, the positive control and salt-processed groups both showed marked decreases. In terms of energy metabolism, both raw and salt-processed PAC significantly reduced Na^+^-K^+^-ATPase activity on erythrocyte membranes, effectively improving abnormal energy metabolism in hyperthyroidism-induced yin-deficient rats, with the effect of the salt-processed preparation being slightly weaker than that of the raw form. Based on the collective changes in these indicators, the study concluded that both raw and salt-processed PAC possess the effects of nourishing kidney yin and clearing deficiency heat, though their characteristics differ: salt-processed PAC demonstrates a stronger action in nourishing kidney yin, while the raw form exhibits a more pronounced effect in clearing deficiency heat.

### 8.8. Effects on the Cardiovascular System

PAC exerts multifaceted protective effects on the cardiovascular system, with mechanisms potentially involving the inhibition of oxygen free radical generation, alleviation of lipid peroxidation, protection of ischemic myocardium, antiarrhythmic activity, and blood pressure reduction through integrated regulatory pathways [95]. Research by Qi [100], using rat models of myocardial injury induced by pituitrin and isoproterenol hydrochloride, demonstrated that both the aqueous and alcoholic extracts of PAC (both at 0.06 g/mL) exhibit protective effects against myocardial ischemia. The underlying mechanism may involve the inhibition of oxygen free radical generation and mitigation of lipid peroxidation. This conclusion is supported by observations of reduced serum activities of creatine kinase and lactate dehydrogenase, along with elevated SOD activity and decreased malondialdehyde content in myocardial tissue.

Dong [101] by establishing a guinea pig model, found that berberine exerts concentration-dependent effects on calcium channels in cardiomyocytes. In vitro, at 1 μM, berberine significantly inhibits extracellular calcium influx induced by 30 mM KCl in guinea pig cardiomyocytes (*p* < 0.01). However, when the concentration is increased to 100 μM, it markedly stimulates calcium release from intracellular calcium stores (*p* < 0.01). Additionally, Wang [99] reported that intravenous administration of PAC capsules containing berberine in dogs resulted in a marked reduction in blood pressure, with the hypotensive effect lasting for more than 2 h, and no rapid tolerance phenomenon was observed.

### 8.9. Anti-Ulcer Effect

As demonstrated by Tong [102], the water-soluble components of PAC exert an inhibitory effect on gastric acid secretion. In vivo, despite lacking significant effects on gastric mucosal SOD activity in normoxic mice or on gastric mucosal blood flow in rats, these components mitigated the stress-induced suppression of SOD activity following water immersion restraint. Additionally, they inhibited the reduction in prostaglandin E_2_ (PGE_2_) in the rat gastric mucosa caused by indomethacin and increased PGE_2_ levels in the gastric mucosa of normal mice. These results indicate that PAC possesses certain anti-gastric ulcer activity, which is closely related to the regulatory mechanism involving gastric mucosal PGE_2_ levels.

Wang and colleagues [103] found that the total alkaloids of PAC—primarily composed of jatrorrhizine, palmatine, and berberine (collectively accounting for approximately 90.74%)—exhibit significant gastroprotective effects against acetic acid-induced chronic gastric ulcers in rats. In vivo, a chronic gastric ulcer model was induced in mice by applying the acetic acid (0.14 M) filter paper method to the serosal layer of the gastric mucosa. After oral administration, the ulcer area in the medium-dose total alkaloids group (50 mg/kg) (7.67 ± 2.06 mm^2^) was significantly reduced compared to the model group (15.15 ± 2.34 mm^2^) (*p* < 0.01), with an ulcer inhibition rate of 49%, which outperformed the clinical drug omeprazole (46%). In contrast, the low-dose (10 mg/kg) and high-dose (90 mg/kg) groups showed no significant protective effects, indicating that its efficacy is not dose-dependent. Histopathological observations revealed that medium-dose total alkaloids significantly alleviated gastric mucosal epithelial detachment, necrosis, and inflammatory cell infiltration, promoting the restoration of gastric mucosal structure to near-normal levels.

Mechanistic studies showed that medium-dose total alkaloids of PAC significantly increased serum epidermal growth factor levels (promoting mucosal regeneration and ulcer healing) while upregulating cerebral serotonin (regulating mood and improving stress responses) and adrenal norepinephrine levels (improving local tissue blood supply and promoting damage repair) (all *p* < 0.01). This suggests that total alkaloids may accelerate ulcer healing and improve healing quality through neurohumoral regulatory mechanisms. In summary, the total alkaloids of PAC exert gastroprotective effects by modulating epidermal growth factor and neurotransmitter (serotonin, norepinephrine) levels, with the medium dose identified as the effective dosage. These findings highlight its potential for further development as a clinical anti-gastric ulcer drug.

Nonsteroidal anti-inflammatory drugs can cause intestinal injury and ulcers, with an increasing incidence. Limonin plays a regulatory role in inflammatory diseases, but no reports have described its use in treating intestinal injury and ulcers. Jia and colleagues [104] established a rat model of intestinal injury and ulceration using indomethacin. They analyzed the docking between limonin and nuclear factor erythroid 2-related factor 2 (Nrf2) via Western blot and molecular docking, and treated indomethacin-exposed IEC-6 cells with ML385 (an Nrf2/antioxidant response element (ARE) pathway inhibitor) to assess cell viability, apoptosis, and inflammation. In vivo, results showed that limonin effectively ameliorated indomethacin-induced ulcerative intestinal injury, reduced small intestinal inflammation and oxidative stress, and repaired barrier dysfunction. Further research has found that limonin docked with and activated the Nrf2/ARE pathway, while ML385 reversed its protective effects. This study demonstrates that limonin ameliorates indomethacin-induced intestinal injury and ulcers via the Nrf2/ARE signaling pathway [104].

### 8.10. Effects on Articular Chondrocytes

The extract of PAC significantly regulates the metabolism of articular cartilage. Using human osteoarthritic cartilage tissue blocks as experimental material, studies have shown that both 5% and 10% PAC injections can inhibit DNA and collagen synthesis in chondrocytes. However, regarding proteoglycan synthesis, the 5% injection promotes it, while the 10% concentration does not exhibit this effect. Furthermore, PAC ethanol extract (40–200 μg/mL) demonstrates protective effects on IL-1*α*-induced human osteoarthritis cartilage explants and chondrocytes. It concentration-dependently inhibits the degradation of glycosaminoglycans and type II collagen, downregulates the expression of protein hydrolases such as aggrecanase-1/-2 and MMP-1/-3/-13, upregulates tissue inhibitor of metalloproteinases 1 activity, and attenuates the phosphorylation of extracellular regulated protein kinase 1/2, c-Jun *N*-terminal kinase, and p38 MAPK. Compared to celecoxib, PAC extract exhibits a more comprehensive profile in protecting cartilage matrix and inhibiting inflammation, showing potential for multi-target intervention in delaying osteoarthritis progression [105].

Lee and colleagues [106], through studies using a peripubertal female rat model, reported that PAC extract (100 mg/kg) increased bone growth rate and upregulated insulin-like growth factor 1 and bone morphogenetic protein 2 in the growth plate, with no effect on vaginal opening time or reproductive organ weights. This suggests PAC promotes bone growth via chondrocyte proliferation and differentiation without affecting pubertal development. In summary, PAC can inhibit the destruction of articular cartilage and chondrocytes through the aforementioned mechanisms, positioning it as a potential therapeutic agent for osteoarthritis.

Further research on palmatine elucidates its mechanisms for joint protection. In vivo (mouse acute gout arthritis model, air pouch gout model), palmatine dose-dependently alleviated MSU-induced joint swelling and inflammatory cell infiltration, and downregulated the expression of the macrophage marker F4/80. Its anti-inflammatory effect at a dose of 100 mg/kg was superior to that of the commonly used clinical drug colchicine. Mechanistic studies revealed that palmatine blocks NLRP3 inflammasome activation, attenuates the levels of inflammasome-associated proteins such as NLRP3, ASC, caspase-1, IL-1*β*, and high mobility group box 1, inhibits caspase-11-mediated non-canonical NLRP3 pathway activation, and decreases the expression of proteins associated with neutrophil extracellular trap formation (caspase-11, histone H3, proteinase 3, peptidylarginine deiminase 4), thereby suppressing pyroptosis and interrupting the inflammatory cascade. Simultaneously, palmatine significantly downregulated MMP-3 expression, reduced proteoglycan loss in cartilage, improved cartilage surface damage, and maintained the structural integrity of articular cartilage [64]. In conclusion, palmatine, by targeting the regulation of the NLRP3 inflammasome pathway, concurrently exerts anti-inflammatory, anti-pyroptotic, and cartilage-protective effects. It demonstrates the advantage of multi-target synergy in the treatment of gouty arthritis, exhibits high safety, and holds potential for development as a novel natural anti-gout drug.

### 8.11. Anti-Gout Effect

According to TCM theory, gout is primarily attributed to the internal accumulation of dampness and turbid toxins. Clinical treatment typically follows the fundamental principle of clearing heat and promoting diuresis to eliminate dampness. PAC, with its properties of clearing heat and drying dampness, is widely used in the treatment of gout. Research by Lian [107] demonstrated that salt-processed, raw, and stir-fried PAC can all reduce serum uric acid and creatinine levels in hyperuricemic model rats and inhibit joint swelling in rats with acute gouty arthritis. No significant differences in anti-gout effects were observed among the different processed products. While processing may lead to the formation of components such as berberrubine, the specific relationship between processing-induced compositional changes and anti-gout effects, as well as the primary active components, requires further in-depth clarification.

Yang and colleagues [108] established a mouse model of hyperuricemia, using mouse serum uric acid levels and hepatic xanthine oxidase activity as evaluation indicators and confirmed that both raw and salt-processed PAC at low and high doses can reduce uric acid and inhibit xanthine oxidase activity. Furthermore, high doses showed no significant effect on uric acid levels in normal animals. Research by Pan [109] also indicated that administration of PAC extract to hyperuricemic mice significantly decreased their serum uric acid levels.

Yue and colleagues established an MSU-induced acute gouty arthritis mouse model and discovered that the core mechanisms are as follows: (1) Immunomodulation and anti-inflammatory effects: Inhibiting NLRP3 inflammasome activation and caspase-1-mediated pyroptosis; blocking the toll-like receptor 4/NF-*κ*B pathway to reduce the release of pro-inflammatory cytokines such as IL-1*β* and TNF-*α*; activating the Nrf2 antioxidant pathway to alleviate oxidative stress; and regulating immune responses by correcting the T helper 17 cell/Treg cell imbalance. (2) Uric acid reduction and renal protection: Its metabolites (e.g., berberrubine, dihydroberberine) can block xanthine oxidase activity, leading to reduced uric acid synthesis. Simultaneously, they inhibit the expression of the renal urate transporter 1, promote uric acid excretion, and alleviate kidney damage caused by urate crystals. (3) Maintaining gut microbiota balance: Modulating gut microbiota diversity by enriching beneficial bacteria (e.g., Bifidobacterium) while suppressing pathogenic populations, thereby facilitating intestinal purine and uric acid metabolism. (4) Regulating apoptosis and bone metabolism: Inducing neutrophil apoptosis to alleviate inflammation; inhibiting chondrocyte apoptosis to protect joint structure; promoting osteoblast proliferation and inhibiting osteoclast differentiation, thereby improving gout-related bone erosion and osteoporosis.

In summary, berberine may intervene in the entire pathogenesis of gout through synergistic multi-pathway actions. With advantages such as natural origin, low toxicity, and wide availability, it demonstrates potential for development as a candidate drug for gout treatment. However, its specific active components and detailed pharmacological mechanisms still require further systematic investigation [18].

### 8.12. Effects on Kidney Protection and Disease Treatment

A study by Liu demonstrated that PAC can reduce renal pathological damage in rats with uric acid nephropathy, lower blood urea nitrogen, creatinine, and uric acid levels, and decrease the expression of TNF-*α* and COX-2 in renal tissue. Its mode of action may be linked to decreased uric acid levels, reduced urate deposition in renal tubules, and inhibition of inflammatory responses, providing experimental evidence for the use of PAC in treating gout and hyperuricemia [110]. Zhang and colleagues [111] established a nephritis rat model by injecting 6.5 mg/kg doxorubicin via the tail vein. Further metabolomic and biochemical analyses revealed that PAC significantly improves renal function in adriamycin-induced nephritis rats, specifically by reducing urinary protein and serum creatinine, and increasing serum total protein and albumin. This effect involves the modulation of 28 differential metabolites (such as 5′-methylthioadenosine) and the regulation of nine related metabolic pathways (e.g., glutamate metabolism). PAC was also found to differentially regulate renal protein expression, increasing *Cda* and *Tk* levels while decreasing *Mtap* and *Ass*. These changes point to a renoprotective mechanism potentially mediated through anti-inflammatory, antioxidant, and immunomodulatory pathways.

In a mouse model of diabetic nephropathy, berberine inhibited the sphingosine kinase 1-sphingosine-1-phosphate-sphingosine-1-phosphate receptor 2 receptor pathway, reduced the expression of sphingosine-1-phosphate receptor 2 receptors in the kidneys of diabetic rats and in high glucose-treated glomerular mesangial cells, thereby suppressing the overproduction of fibronectin. Additionally, berberine inhibited high glucose-induced NF-*κ*B activation, improved blood glucose and renal function indicators (such as blood urea nitrogen, creatinine, and 24 h urinary protein) in rats, and alleviated renal fibrosis [112].

Phellodendrine also exhibited anti-nephritis activity in crescentic anti-glomerular basement membrane nephritis rats. In vivo, oral administration of phellodendrine (25–100 mg/kg/d) significantly inhibited urinary protein excretion (with an inhibition rate of 82.3% on day 19 in the 100 mg/kg group), lowered plasma cholesterol and creatinine levels, reduced glomerular crescent formation (approximately 70% inhibition), adhesion, fibrinoid necrosis, and associated inflammatory cell infiltration, without affecting plasma anti-rabbit *γ*-globulin antibody titers. Its mechanism of action may involve attenuating the proliferation or chemotaxis of macrophages and cytotoxic T cells [113].

A study by Xu [114] found that dihydroberberine can inhibit xanthine oxidase activity in vitro, while in vivo, after establishing a mouse model of hyperuricemia, it reduced serum uric acid and xanthine oxidase levels, inhibits hepatic xanthine oxidase and adenosine deaminase activities, and downregulates the mRNA and protein expression of renal xanthine oxidase. Furthermore, dihydroberberine dose-dependently lowered serum creatinine, blood urea nitrogen, and inflammatory cytokine levels, improving renal damage induced by hyperuricemia. Research by Rong [115] further confirmed that berberine has clear uric acid-lowering and renal protective effects in an oxonic potassium/hypoxanthine-induced hyperuricemia mouse model. Its mechanism may involve regulating the balance of urate transporter reabsorption and secretion, as well as inhibiting the JAK2/STAT3 signaling pathway.

Ai and colleagues [116] demonstrated that palmatine exerts anti-hyperuricemic and renal protective effects through multi-target regulation: In vivo, in a postoperative/hypoxia-induced hyperuricemia mouse model, palmatine reduced serum uric acid, creatinine, and urea nitrogen levels, and ameliorated renal pathological damage. The mechanism involves inhibition of hepatic xanthine oxidase and adenosine deaminase activities, downregulation of glucose transporter type 9 and urate transporter 1 expression, upregulation of organic anion transporter 1 and ATP-binding cassette superfamily G member 2 to promote uric acid excretion, along with activation of the Kelch-like ECH-associated protein 1-Nrf2 pathway for antioxidant effects and inhibition of thioredoxin-interacting protein/NLRP3 inflammasome activation. Molecular docking confirmed that palmatine stably binds to Kelch-like ECH-associated protein 1, NLRP3, urate transporter 1, and heme oxygenase-1. Tan and colleagues [117] reported for the first time that oxyberberine exhibits superior uric acid-lowering effects compared to berberine in a high-fructose-induced hyperuricemia rat model, with mechanisms involving inhibition of endogenous purine synthesis, alleviation of inflammation and oxidative stress, and modulation of gut microbiota (increasing beneficial bacteria while reducing harmful bacteria). Together, these studies reveal the mechanisms by which active compounds from PAC treat hyperuricemia through regulation of uric acid metabolism, oxidative stress, and inflammatory pathways.

Zheng and colleagues [118] found that berberine exerts a protective effect on uric acid-induced human kidney-2 (HK-2) cells, and its mechanism is related to the regulation of the NLRP3 signaling pathway. In vitro, in a uric acid-stimulated HK-2 cell model, berberine significantly reduced the levels of inflammatory factors (IL-1*β*, IL-18) and lactate dehydrogenase, downregulated the expression of pro-apoptotic proteins (Bax, cleaved-caspase-3/9), and upregulated the expression of the anti-apoptotic protein Bcl-2. Concurrently, it inhibited NLRP3 inflammasome activation, reducing the expression of pathway-related proteins including NLRP3, ASC, Caspase1, IL-18, IL-1*β*, and gasdermin D. Further validation using NLRP3-Small interfering RNA demonstrated that silencing NLRP3 enhanced the ameliorative effect of berberine on uric acid-induced cell injury.

Mei and colleagues [19] found that PAC polysaccharide, composed of rhamnose, galacturonic acid, galactose, and *D*-xylose with a molecular weight of 1.98 × 10^5^ Da, ameliorates diabetic nephropathy through multi-target mechanisms. In vivo, in a streptozotocin-induced diabetic nephropathy rat model and a high glucose-stimulated HK-2 cell model, PAC polysaccharide enhanced antioxidant capacity by upregulating the PI3K/AKT pathway, alleviated renal fibrosis and inhibited apoptosis by activating the transforming growth factor-beta/Smad pathway via Nrf2, and suppressed renal inflammation by modulating gut microbiota. This study systematically elucidates the therapeutic mechanisms of PAC polysaccharide in diabetic nephropathy through multiple pathways, including gut microbiota regulation, enhancement of antioxidant and anti-fibrotic capacities, and inhibition of apoptosis.

### 8.13. Hepatic Protective and Therapeutic Effects

A study by Wang [119] discussed the oxidative stress mechanisms of TCM-induced Drug-Induced Liver Injury and the antagonistic effects of common antioxidant Chinese herbs against Drug-Induced Liver Injury, revealing that oxidative damage to cell membranes or abnormalities in biotransformation are key factors in the occurrence of liver injury. PAC can effectively alleviate liver damage induced by *Dioscorea bulbifera*, reduce hepatocyte necrosis, and prevent the development of liver fibrosis and steatosis. Additionally, using a mouse model with a GST activator, it was found that a glutathione-*S*-transferase activator purified from the aqueous extract of PAC can promote liver detoxification function, holding significant application value in the development of detoxifying drugs or functional foods for liver protection [120].

Cheng and colleagues [121] demonstrated that berberine ameliorates fructose-induced liver injury through the adenosine kinase (ADK)/AMPK/Nrf2 signaling pathway. In vivo, in a fructose-induced rat model and hepatocellular carcinoma-G2/buffalo rat liver-3A cell models, berberine reduced weight gain, glucose intolerance, and insulin resistance, decreased serum alanine aminotransferase/aspartate aminotransferase activities, and improved histopathological damage. The mechanism involves inhibition of inflammatory factors (IL-6, TNF-*α*), promotion of anti-inflammatory factor (IL-10) release, attenuation of oxidative stress (reducing ROS/MDA, increasing SOD/GSH), along with upregulation of Nrf2, heme oxygenase-1, and p-AMPK expression, elevation of adenosine monophosphate content and adenosine monophosphate/adenosine triphosphate ratio, and promotion of ADK expression. Silencing ADK abolished the hepatoprotective effects of berberine, confirming that the ADK/AMPK/Nrf2 pathway is a key regulatory mechanism.

### 8.14. Therapeutic Effects on Skin Diseases

PAC is widely used in dermatology, exhibiting anti-allergic, antioxidant, and antibacterial effects. In terms of anti-allergic properties, Song and colleagues [122] established a mouse model of allergic contact dermatitis (ACD) induced by 2,4-dinitrofluorobenzene (DNFB) and demonstrated that the aqueous decoction of PAC significantly inhibits delayed-type hypersensitivity in mice in a dose-dependent manner. The mechanism may involve suppressing the production and secretion of cytokines such as IFN-*γ* and IL-2, thereby mitigating inflammatory damage. Antioxidant experiments have shown that both aqueous and alcoholic extracts of raw PAC can scavenge superoxide anion radicals and hydroxyl radicals, with the aqueous extract demonstrating stronger scavenging capacity. However, the aqueous extract is less effective than the alcoholic extract in inhibiting lipid peroxidation [123]. In antibacterial experiments, a compound formulation containing PAC combined with *Forsythia suspensa* and *Scolopendra subspinipes*, among other herbs, achieved a killing rate of over 95% against natural flora on the skin surface. This provides a basis for the use of PAC in treating skin conditions related to delayed-type hypersensitivity, such as eczema and contact dermatitis [124]. Additionally, through experiments using CCD-986sk human dermal fibroblasts, a study found that jatrorrhizine isolated from PAC exhibits no cytotoxicity at concentrations ≤ 10 μM. It upregulates the expression of genes such as COL1A2 and tissue inhibitor of metalloproteinases 1, promotes the synthesis of type I procollagen and hyaluronic acid, while inhibiting the expression of MMP-1 and MMP-9 genes. Consequently, it improves collagen homeostasis and inhibits UV-B-induced damage to skin fibroblasts. This finding suggests that jatrorrhizine has potential applications in anti-wrinkle cosmetics and skin repair [125]. Cho and colleagues [126], through experiments using B16F10 mouse melanoma cells, found that jateorhizine isolated from PAC significantly inhibits *α*-melanocyte-stimulating hormone-induced melanogenesis. Treatment with 20–100 μM jateorhizine suppressed the melanocortin 1 receptor, increased transforming growth factor-beta 1 levels, downregulated the expression and enzyme activity of microphthalmia-associated transcription factor, tyrosinase, and tyrosinase-related proteins (tyrosinase, tyrosinase-related protein 1, tyrosinase-related protein 2), while also inhibiting the expression of melanosome transport proteins RAB27A and MYO5A. This study demonstrates that jateorhizine exerts dual inhibitory effects on melanin synthesis and transport, suggesting its potential application in functional cosmetics and beauty food products.

### 8.15. Effects on the Prostate

PAC and its components exert multifaceted interventional effects on prostate-related diseases. Nexrutine exhibits anti-prostate cancer effects by inhibiting prostate cancer cell proliferation and tumor development in transgenic adenocarcinoma of the mouse prostate mice, partly through Akt and cAMP responsive element binding protein (CREB). COX-2, a CREB target gene, promotes PGE_2_ production and inhibits apoptosis. In vitro, Nexrutine reduced COX-2 activity and expression in lymph node carcinoma of the prostate and prostate cancer 3 cells via the CRE sequence of the COX-2 promoter. In human high-grade prostate tumors, CREB expression and activity were three times higher than in normal tissue (*p* = 0.01). These findings confirm that CREB-mediated COX-2 activation is a key pathway in prostate cancer, and that Nexrutine, as a safe, low-cost dietary supplement, can block this pathway, supporting its potential for prostate cancer prevention and treatment [17]. It also suppresses the Akt/CREB/NF-*κ*B signaling pathway, inducing apoptosis in tumor cells. In vitro, it inhibits the proliferation of lymph node carcinoma of the prostate and prostate cancer 3 cells (with an IC_50_ of approximately 5 mg/mL), while in vivo it reduces tumor volume by over 50% in transgenic adenocarcinoma of the mouse prostate mice and demonstrates high safety [127].

A clinical study enrolled 21 prostate cancer patients who underwent surgery or radiotherapy and received oral Nexrutine (500 mg, three times daily). The preoperative group took the medication for 1–2 months before surgery, while the radiotherapy group received it for 1–2 months alongside radiotherapy. The results showed good tolerability of the extract: only two transient grade 3 toxicities (hypokalemia and urinary incontinence) occurred, with no grade 4 or higher adverse reactions; the remaining side effects were mild and self-limiting. At the end of neoadjuvant therapy, 81% of patients experienced a decrease in prostate-specific antigen levels, with an average reduction of 17% (ranging from 2% to 42%) [128].

In vivo, in a chronic nonbacterial prostatitis (CNP) model, alkaloids from PAC significantly improved eye movement and activity scores in capsaicin-induced CNP rats, reduced prostate protein exudation, and alleviated histopathological damage (acinar structure became more uniform, with decreased vascular congestion and inflammatory cell infiltration), indicating their potential to intervene in the pathological process of CNP [129]. Regarding benign prostatic hyperplasia, berberine can improve the condition through synergistic actions such as inhibiting the NF-*κ*B pathway and regulating macrophage polarization. Its anti-inflammatory and antioxidant effects are superior to those of finasteride [130]. Additionally, in vitro, palmatine from PAC selectively inhibits the proliferation and invasion of prostate cancer cells (e.g., DU145), achieving a 50% inhibition rate at a concentration of 10 μg/mL. Its mechanism is related to downregulating phosphorylated ribosomal protein S6 and blocking NF-*κ*B transcriptional activity and downregulating its downstream gene FLICE-inhibitory protein [78].

### 8.16. Insecticidal Activity

Bioassay results showed that continuous feeding of larvae with a diet containing 5.95 mM berberine for 7 d resulted in a mortality rate of 70%, with a median lethal dose (LD_50_) of 2.44 mM. The insecticidal activity of berberine was comparable to that of (–)-tetrahydrocoptisine, and its toxicity was higher than that of (–)-7-epihydrocubebin. In topical application tests on adult *Drosophila melanogaster*, berberine exhibited the strongest activity, achieving a mortality rate of 70% at a dose of 20 μg per adult, with a median LD_50_ of 19.0 μg per individual. Its activity surpassed that of the other compounds tested [131].

Through sequential solvent extraction and chromatographic purification, 4 limonoid compounds (obacunone, limonin, kihadanin A, and kihadanin B) and 2 isoquinoline alkaloids (berberine hydrochloride, palmatine iodide) were isolated from the methanol extract of PAC. Bioactivity tests revealed that obacunone possessed the strongest antifeedant activity, with a 30-day survival rate of termites of only 13.0% at a dose of 100 μg/dish. Berberine hydrochloride and palmatine iodide followed, with 30 d survival rates of 16.7% and 20.0%, respectively, at a dose of 300 μg/dish. Kihadanin A exhibited weaker activity, showing a 20.0% survival rate at a dose of 1200 μg/dish, while limonin and kihadanin B displayed no significant antifeedant activity. Structure-activity relationship studies demonstrated that the furan ring moiety is critical for antifeedant effects: obacunone, which contains a furan ring, showed significant activity; its oxidized derivative kihadanin A, with a modified furan ring, exhibited reduced activity; and kihadanin B, lacking the furan ring, was completely inactive. This finding also explains why a certain Japanese millennium-old scroll containing PAC extract remained undamaged by pests, confirming that the limonoid and alkaloid components in PAC are effective natural termite antifeedants [132].

### 8.17. Protective Effects on the Lungs

The effect of a methanol extract of PAC on acute airway inflammation was induced by intranasal instillation of LPS (300 μg/kg) in female BALB/c mice. In vivo, within the dose range of 100–400 mg/kg, methanol extract of PAC dose-dependently alleviated inflammatory responses, reduced total cell count, neutrophil count, and protein concentration in bronchoalveolar lavage fluid, while also downregulating pro-inflammatory cytokines, including TNF-*α*, Macrophage Inflammatory Protein-2, and NO. Its mechanism of action may be related to inhibiting inflammatory cell infiltration and the release of inflammatory mediators, suggesting that methanol extract of PAC holds potential therapeutic value for infectious pulmonary diseases [133]. Furthermore, studies indicate that dietary intake of berberine and a PAC extract containing 33% berberine can inhibit lung cancer cell proliferation and tumor growth in vivo. The mechanism involves inducing G1 phase cell cycle arrest and inhibiting the Akt/CREB/MAPK phosphorylation signaling pathway.

Kim and colleagues [134] found that *P. amurense* exerts significant anti-asthmatic effects in an ovalbumin-induced mouse asthma model. In vivo, PAC inhibited airway eosinophil accumulation, inflammatory cell infiltration, and airway hyperresponsiveness, reduced the levels of Th2-type cytokines (IL-4, IL-5, IL-13) and TNF-*α* in bronchoalveolar lavage fluid and lung tissue, and decreased serum ovalbumin-specific IgE. Additionally, it suppressed the gene expression of IL-4, IL-5, IL-13, thymus and activation-regulated chemokine, and C-C chemokine receptor type 3, and alleviated airway inflammation-related pathological changes in lung tissue, including inflammatory cell infiltration, collagen deposition, and goblet cell hyperplasia. This study demonstrates that PAC prevents and treats allergic asthma by inhibiting cytokines, chemokines, and chemokine receptors associated with allergic inflammation.

Childhood asthma is a common chronic disease characterized by airway inflammation, remodeling, and oxidative stress. Rutaecarpine is a bioactive alkaloid with therapeutic effects on allergic diseases, but its role in asthma remains unclear. Chen and colleagues [135] established an ovalbumin (OVA)-induced mouse asthma model. In vivo, compared with the OVA group, 10 and 20 mg/kg rutaecarpine reduced lung inflammation scores by 37.5% and 50.0%, inhibited IL-4, IL-5, and IL-13 secretion, and decreased total cells, eosinophils, macrophages, neutrophils, and lymphocytes in bronchoalveolar lavage fluid. Rutaecarpine also downregulated α-smooth muscle actin and type I collagen expression, ameliorated OVA-induced mucus hypersecretion, and protected against oxidative lung injury. Mechanistically, rutaecarpine inhibited OVA-induced activation of the NF-*κ*B pathway. In conclusion, rutaecarpine alleviated OVA-induced airway inflammation, remodeling, and oxidative stress by inhibiting the NF-*κ*B pathway, suggesting its potential as an adjunctive therapy for childhood asthma and providing a basis for future clinical translation.

### 8.18. Other Pharmacological Effects

The aqueous extract of PAC exhibits broad-spectrum antiviral activity. By establishing an infection model in BALB/c mice, it was found that PAC markedly inhibits the replication of influenza viruses (including H1N1, H5N2, and other subtypes), herpesviruses, and enteroviruses in immune cells (e.g., RAW 264.7) and epithelial cells (e.g., HEK293T, HeLa) by inducing the secretion of type I interferon (interferon beta) and pro-inflammatory cytokines (such as TNF-*α* and IL-6), and activating signaling pathways including interferon regulatory factor 3, signal transducer and activator of transcription 1, and NF-*κ*B. In vivo experiments demonstrated that administering PAC at 0.8 μg/g body weight enhanced the survival rate of mice challenged with a lethal dose of influenza virus to 60–80% and significantly reduced viral titers in lung tissue [136].

Dengue virus type 2 (DENV2) causes dengue fever and is a global public health concern. The virus relies on the nonstructural protein (NS)3 protease with the NS2B cofactor for replication, making NS3 protease an important antiviral target. Stalin and colleagues [137] investigated Niloticin’s inhibition of the NS2B/NS3 protease through in silico and in vitro analyses. In silico docking predicted that Niloticin forms hydrogen bonds and hydrophobic interactions with residues such as LEU149 and ASN152. Structural analyses showed that the niloticin-NS2B/NS3 protease complex was more stable than the protease alone. In vitro, niloticin had an IC_50_) of 0.14 μM in BHK cells and exhibited concentration-dependent antiviral activity at 2.5 μM. Western blot and qRT-PCR confirmed that niloticin dose-dependently reduced DENV2 protein transcription, and NS3 protease activity assays verified its inhibition. These findings suggest niloticin is a promising antiviral agent against DENV2 and other flaviviruses.

The study found that when bovine embryos post in vitro fertilization were cultured for 7 d in Charles Rosenkrans amino acid medium supplemented with PAC (0.01 μg/mL), their developmental capacity and antioxidant status were significantly affected. The PAC treatment group exhibited a significantly higher blastocyst formation rate (28.9% ± 2.9%) compared to the control group (25.4% ± 1.6%) (*p* < 0.05). Concurrently, the intracellular reactive oxygen species levels in the treated embryos were substantially reduced (*p* < 0.01), and both cleaved caspase-3 expression and apoptotic cell number in blastocysts were significantly diminished (*p* < 0.05). This indicates that the appropriate addition of PAC extract during the pre-implantation culture of bovine embryos can effectively improve blastocyst quality by reducing oxidative stress and cell apoptosis [138].

Castor oil induces diarrhea by stimulating intestinal secretion through its active metabolite ricinoleic acid, without affecting intestinal smooth muscle motor function. By establishing a female Balb/c mouse model it was found that both PAC and PCC treatments significantly inhibit this type of diarrhea, with no notable difference in efficacy between the two. It is worth emphasizing that although the berberine content in PCC is much higher than that in PAC (240 mg/kg vs. 26 mg/kg, respectively), at the same crude drug dosage (8.0 g/kg), their inhibitory effects on diarrhea are comparable. This suggests that berberine is one of the active components responsible for inhibiting diarrhea, but other chemical constituents in PAC and PCC also contribute synergistically to the overall therapeutic effect [139].

This study investigated the preventive effects of toothpaste containing 0.05% PAC extract (referred to as T-toothpaste) on periodontal disease. Through a comprehensive analysis including in vitro experiments (red blood cell membrane stability and astringency tests), in vivo experiments (carrageenan-induced paw edema and cotton pellet granuloma models), and a clinical trial involving 152 people, it was found that PAC extract at concentrations above 0.05% significantly inhibits inflammatory responses. Specifically, it demonstrated concentration-dependent inhibition of hemolysis, edema, and granuloma tissue formation. The clinical trial showed that after using T-toothpaste for 4 weeks, patients’ phorbol-12-myristate-13-acetate index (an indicator of periodontal inflammation) significantly improved (*p* < 0.01), and the degree of gingival redness was markedly reduced (*p* < 0.05), with no adverse effects observed. These results indicate that toothpaste supplemented with PAC extract holds potential application value in the prevention of periodontal disease [140].

Kim and colleagues [141] found that PAC extract exhibits concentration- and time-dependent antibacterial effects against *Streptococcus mutans*, with complete inactivation of *Streptococcus mutans* at a concentration of 5 mg/mL. Cytotoxicity assays showed that the safety of the extract can be verified by measuring the viability of human keratinocytes (HaCaT). Human keratinocytes cell proliferation slightly decreased at 1.25 mg/mL but did not reach the IC_50_, indicating no significant cytotoxicity at the tested concentrations. This study demonstrates that PAC extract, as a naturally derived anticariogenic agent, can effectively inhibit *Streptococcus mutans* while maintaining cell viability, suggesting its potential for clinical application.

Clinical practice in the dermatology department of Jiading District Hospital of TCM in Shanghai has shown that using a decoction of PAC or a compound PAC lotion to treat eczema can achieve good therapeutic outcomes. The specific method involves diluting the PAC liquid to an appropriate concentration, soaking gauze in the medicated solution, and applying it as a wet compress to the affected area. This treatment approach has demonstrated significant effectiveness in clinical applications [142].

Solar UV radiation is a major cause of skin aging. Ryu and colleagues used UV-irradiated human keratinocytes as a model to investigate the anti-wrinkle effects of lotusine. The results showed that lotusine reduced UV-induced MMP-1 expression, modulated the transcriptional activities of activator protein-1 and NF-*κ*B, and inhibited multiple signaling pathways, including MEK1/2-ERK1/2-p90RSK, MKK3/6-p38, and AKT-p70S6K. These findings indicate that lotusine ameliorates UV-induced MMP-1 overexpression and skin aging, demonstrating its potential as an ingredient for functional foods and cosmetic products [143].

He and colleagues [144], through in vitro animal experiments, demonstrated that berberine alleviated intestinal mucosal mechanical barrier injury in diclofenac-induced enteropathy by inhibiting exosomal long non-coding RNA (lncRNA) H19 and promoting autophagy recovery. In vivo, in a diclofenac-induced enteropathy model, berberine downregulated lncRNA H19 expression in both the small intestine and exosomes. Exosome co-culture experiments revealed that exosomes derived from the diclofenac group carried high levels of lncRNA H19, which suppressed the expression of autophagy-related proteins (Atg5, LC3) and tight junction proteins (zonula occludens protein 1, claudin-1, occludin). Berberine treatment reduced exosomal lncRNA H19 levels, upregulated autophagy and tight junction protein expression, and increased the LC3-II/LC3-I ratio. This study uncovers the mechanism by which exosomal lncRNA H19 affects the intestinal mucosal barrier through autophagy regulation, providing a new perspective for the use of berberine in treating diclofenac-induced enteropathy.

Yu and colleagues [145] used a pseudo-germ-free mouse model, 16S rRNA gene sequencing, and transcriptome analysis, and found that berberine exerts its anti-ulcerative colitis effects in a gut microbiota-dependent manner. In vivo, in a dextran sulfate sodium-induced colitis model, berberine ameliorated colonic damage and related indicators only in mice with intact gut microbiota, but showed no effect in pseudo-germ-free mice. Mechanistic studies revealed that berberine, mediated by the gut microbiota, inhibits the phospholipase A_2_-cyclooxygenase-2-prostaglandin E_2_-E-prostanoid receptor 2 pathway and reduces the expression of PGE_2_, PLA_2_, and related factors, thereby alleviating colonic inflammation. This study reveals the gut microbiota-dependent mechanism of berberine in the treatment of ulcerative colitis.

Wang and colleagues [146] established a C48/80-induced mouse paw edema model and found that phellodendrine treats allergic diseases by targeting MAS-related G protein-coupled receptor, member B3/MAS-related G protein-coupled receptor, member X2 receptors. In vivo, in a C48/80-induced allergic model, phellodendrine reduced mouse paw swelling and vascular leakage, inhibited rat basophilic leukemia-2H3 cell degranulation and the release of beta-*N*-acetylhexosaminidase, histamine, IL-4, and TNF-*α*. Mechanistically, phellodendrine altered receptor conformation, reduced MAS-related G protein-coupled receptor, member B3 expression and MAS-related G protein-coupled receptor, member X2 reactivity, and inhibited Ca^2+^ influx and phosphorylation of PLC, MAPK, and NF-*κ*B pathways, thereby blocking allergic reactions. Xu and colleagues [147] established a mouse model of ulcerative colitis and a human colonic epithelial cell model, demonstrated that Nexrutine ameliorates ulcerative colitis by suppressing the key target gene v-rel reticuloendotheliosis viral oncogene homolog A. In vivo, in a dextran sulfate sodium-induced colitis model, 300 or 600 mg/kg Nexrutine reduced disease activity index, alleviated inflammation and pathological damage, and protected the intestinal mucosal barrier; overexpression of v-rel reticuloendotheliosis viral oncogene homolog A reversed these protective effects. Together, these two studies reveal the therapeutic mechanisms of active compounds from PAC in allergic diseases and inflammatory bowel disease, respectively.

Yang and colleagues [148] prepared berberine microcapsules using solid dispersion and microencapsulation technology, and confirmed their favorable intestinal sustained-release properties and stability in a *Salmonella enteritidis*-infected mouse model. In vivo, at 40 mg/kg, berberine microcapsules effectively alleviated intestinal damage, regulated inflammatory factors and immunoglobulins, and improved gut microbiota imbalance (eliminating pathogenic bacteria while increasing beneficial bacteria), demonstrating outstanding effects in anti-inflammation, antibacterial activity, and intestinal barrier repair. Li and colleagues [149] found that phellodendrine ameliorates acute kidney injury and acute lung injury induced by abdominal sepsis through inhibition of the AKT/NF-*κ*B signaling pathway. In vivo, in a rat model of sepsis induced by intraperitoneal fecal suspension injection, phellodendrine increased survival rate, reduced plasma pro-inflammatory factor levels, and attenuated pathological damage in kidney and lung tissues. These two studies reveal the protective effects of active compounds from PAC in intestinal infection and sepsis-induced organ injury, respectively.

Human metapneumovirus (hMPV) causes severe respiratory diseases worldwide. *γ*-Fagarine has anti-tumor, anti-inflammatory, and anti-viral activities, but its effects on respiratory virus infections remain unclear. Using Vero-E6 and 16HBE cells, Li and colleagues [150] found that *γ*-Fagarine showed no obvious cytotoxicity and exhibited anti-hMPV activity, reducing viral titers to 33% and 45% of infected controls, respectively. Mechanistically, *γ*-Fagarine inhibited hMPV infection by blocking viral binding to heparan sulfate proteoglycans and regulating lysosomal pH. In vivo, in hMPV-infected mice, oral *γ*-Fagarine (25 mg/kg) reduced lung viral load and alleviated lung pathology. These findings demonstrate that *γ*-Fagarine exerts anti-hMPV effects in vitro and in vivo via dual mechanisms, suggesting it as a potential candidate against hMPV and other respiratory viruses such as influenza virus and SARS-CoV-2.

### 8.19. Synthesis of Pharmacological Evidence

Collectively, the pharmacological evidence reviewed above reveals converging mechanisms that center on several common signaling pathways, including NF-*κ*B, MAPK, PI3K/AKT, and NLRP3 inflammasome. As summarized in Table 9, berberine is the most extensively studied active compound, followed by palmatine, phellodendrine, and limonoids. Recurring therapeutic themes—such as anti-inflammation, antioxidant stress, hypoglycemic activity, and organ protection—emerge across multiple disease models, indicating that PAC exerts its pleiotropic effects through a limited set of core molecular hubs rather than isolated, unrelated mechanisms. This synthesis underscores the value of targeting these convergent pathways for future drug development and mechanistic exploration.

### 8.20. Critical Evaluation of Pharmacological Evidence

The pharmacological studies reviewed above collectively demonstrate that PAC and its bioactive constituents exert a broad spectrum of beneficial effects in diverse preclinical models. However, it is important to interpret these findings within a hierarchical framework of evidence strength. Computational approaches, such as network pharmacology and molecular docking, are valuable for generating hypotheses by predicting potential targets and pathways; their results should be regarded as *exploratory* rather than confirmatory. In vitro experiments (e.g., cell-based assays) provide mechanistic insights into specific molecular events, such as enzyme inhibition or receptor modulation, but do not fully recapitulate the complex in vivo environment. In vivo animal studies offer the highest level of preclinical evidence within this review, demonstrating pharmacokinetic activity and therapeutic efficacy in whole organisms; nevertheless, they do not directly equate to clinical outcomes in humans. Throughout this section, we have used cautious language-such as “suggest,” “may,” “indicate,” and “potential”-to reflect these distinctions. Readers are encouraged to avoid overinterpreting any single study, particularly those relying solely on in silico predictions or isolated cell-based assays, and to consider the totality of evidence across multiple experimental paradigms. Future research should prioritize well-designed in vivo studies and, where possible, clinical trials to validate the most promising activities of PAC.

## 9. Safety Evaluation

A comprehensive understanding of the safety profile of PAC is essential for evaluating its translational potential for clinical and pharmaceutical applications. This section systematically summarizes the available toxicity evidence, identifies major safety concerns, critically assesses the limitations of existing toxicological data, and provides an integrated translational safety assessment.

### 9.1. Summary of Available Toxicity Evidence

Toxicological studies on PAC have focused primarily on two types of preparations: standardized extracts (e.g., Nexrutine) and purified alkaloids (primarily berberine). The available evidence is summarized below by study design.

#### 9.1.1. Acute Toxicity Studies

Acute toxicity studies evaluate adverse effects following a single high-dose administration.

Nexrutine (standardized PAC extract): In Wistar rats receiving a single oral gavage dose of 2000 mg/kg body weight, no signs of toxicity or mortality were observed during the observation period. Based on the Globally Harmonized System of Classification and Labelling of Chemicals, substances with an LD_50_ exceeding 2000 mg/kg are generally considered relatively safe. Thus, the LD_50_ of Nexrutine is >2000 mg/kg [151].

Berberine: Acute toxicity studies in rodents have reported LD_50_ values ranging from 329 mg/kg (intraperitoneal) to over 2000 mg/kg (oral), indicating that berberine is relatively safe when administered orally but shows higher toxicity via parenteral routes. Species differences exist, with mice generally showing slightly higher sensitivity than rats.

#### 9.1.2. Subacute and Subchronic Toxicity Studies

Subacute (28 d) and subchronic (90 d) studies evaluate repeated-dose toxicity. Nexrutine (28 d oral study): Rats received Nexrutine at doses of 250, 500, or 750 mg/kg/d. No overt toxic effects were observed at any dose. Relative organ weights remained unchanged except for a slight reduction in kidney weight at the highest dose (750 mg/kg). Hematological, biochemical, and histopathological analyses showed no significant abnormalities. At the highest dose, mild decreases in lipid and glucose levels, as well as mild renal tubular degeneration were noted. The no-observed-adverse-effect level for Nexrutine is therefore ≥500 mg/kg/d [151].

Berberine (90 d study): In a 90 d oral toxicity study in rats, berberine administered at doses up to 500 mg/kg/d produced no significant treatment-related adverse effects. Mild gastrointestinal disturbances (transient nausea, loose stools) were observed in some animals at higher doses but resolved without intervention.

#### 9.1.3. Vascular Irritation Studies

Because PAC extracts and berberine have been considered for potential intravenous administration, vascular irritation was evaluated using the chick embryo chorioallantoic membrane assay. At berberine extract concentrations of 0.10 mg/mL and 1.0 mg/mL, no obvious signs of irritation (vasoconstriction, congestion, hemorrhage, or coagulation) were observed, indicating favorable safety at low concentrations, whereas moderate irritation was noted only at the highest tested concentration (10 mg/mL). These results suggest that PAC extracts have acceptable vascular safety at low concentrations, supporting the potential feasibility of intravenous administration at appropriately low doses [152].

#### 9.1.4. Human Safety Data

Clinical safety data for PAC are limited but generally consistent with preclinical findings. Huangbai Capsules (PAC alone): In clinical use, only a small number of patients experienced transient gastrointestinal reactions (e.g., mild nausea); and no liver or kidney function impairment was observed.

Berberine: In clinical studies on diabetes management, berberine was well tolerated. Gastrointestinal side effects (diarrhea, constipation, flatulence) were the most common but were generally mild and transient. No serious adverse events attributable to berberine have been reported in short-term studies (up to 3 months).

### 9.2. Major Safety Concerns

Despite the generally favorable safety profile suggested by available studies, several important safety concerns merit attention.

#### 9.2.1. Herb–Drug Interactions

Berberine, the major alkaloid in PAC, is a known modulator of drug-metabolizing enzymes and transporters. It inhibits several cytochrome P450 (CYP) isoforms, including CYP2D6, CYP3A4, and CYP2C9, at clinically relevant concentrations, raising the potential for interactions with drugs metabolized by these enzymes (e.g., warfarin, statins, calcium channel blockers, and many antidepressants). Additionally, berberine inhibits P-glycoprotein (P-gp), an efflux transporter that affects the absorption and elimination of many drugs. Co-administration with P-gp substrate drugs (e.g., digoxin, fexofenadine) may alter their bioavailability. Therefore, patients taking medications that are CYP450 or P-gp substrates should use PAC or berberine-containing products with caution, and monitoring for potential drug interactions is advisable [153].

#### 9.2.2. Contraindications

Based on TCM theory and modern pharmacological understanding, several precautions should be considered. Regarding pregnancy and lactation, safety data for PAC use during these periods are lacking. Berberine can cross the placenta and has been reported to cause kernicterus in newborns when used near term [154]; therefore, PAC should be avoided during pregnancy and lactation unless specifically prescribed by a qualified practitioner. In TCM theory, PAC is cold in nature and may exacerbate conditions associated with spleen-stomach deficiency (e.g., poor appetite, loose stools, abdominal coldness). Additionally, the safety and efficacy of PAC in pediatric populations have not been systematically evaluated.

#### 9.2.3. Long-Term and High-Dose Use

Available toxicological studies are limited to subchronic durations (maximum 90 days in animals, 3 months in humans), and the safety of PAC or berberine use beyond these durations has not been established. Theoretical concerns include potential cumulative effects on renal tubules based on mild tubular degeneration observed at high doses in animal studies, unknown effects on gut microbiota composition with prolonged use, and the lack of carcinogenicity data.

### 9.3. Limitations of Existing Toxicological Data

While available evidence suggests that PAC has a relatively favorable safety profile at recommended doses for appropriate durations, significant limitations remain. No studies have evaluated PAC safety beyond 90 d in animals or 3 months in humans, and carcinogenicity studies have not been conducted, representing a major gap for long-term use. Reproductive toxicity studies are lacking for PAC extracts, and although berberine has shown embryotoxic effects at high doses in animals, the relevance to therapeutic PAC use remains unclear-a concern given the contraindication during pregnancy. Most toxicological studies rely on conventional endpoints, with no validated safety biomarkers for early or subclinical toxicity. Clinical safety data come primarily from small, short-term studies (*n* < 100, <3 months), and large-scale long-term or post-marketing data are unavailable. No studies have directly compared the safety profiles of PAC versus PCC, or those of different PAC preparations. Finally, safety data for specific populations-including elderly patients, those with hepatic or renal impairment, and patients on multiple medications-are virtually absent.

### 9.4. Comparison with PCC Safety Profile

It is worth noting that no comparative safety studies between PAC and PCC have been published. Given that PCC contains higher levels of berberine (the primary driver of both efficacy and potential toxicity), the safety profiles of the two species may differ. PCC may theoretically carry a higher risk of berberine-related adverse effects (e.g., gastrointestinal disorders, drug interactions) at equivalent crude herb doses. Until comparative data become available, safety conclusions drawn for PAC should not be automatically generalized to PCC, and vice versa.

## 10. Quality Control of PAC

Consistent with the approach taken in Section 8 and Section 9, the quality control evidence presented in this section pertains specifically to PAC. Studies that did not differentiate between PAC and PCC, or that used mixed or unspecified *Phellodendron* species, have been excluded unless they explicitly compared the two species. Quality control is essential for ensuring the safety, efficacy, and consistency of PAC as a medicinal material, particularly given that PAC and PCC were historically conflated under the single name “Huangbai” and that they have significant differences in price and resource availability. Accordingly, this section systematically summarizes the available authentication methods, marker compounds, pharmacopoeial standards, and current analytical challenges for PAC quality control.

### 10.1. Authentication Methods for Species Identification

Accurate species identification is the first and most critical step in PAC quality control, as PAC and PCC differ in their chemical profiles and pharmacological potencies, despite both being used as “Huangbai” in traditional practice.

#### 10.1.1. Morphological and Microscopic Identification

Traditional morphological identification remains the most accessible initial screening method. PAC is characterized by its thin, bright yellow inner bark, which has a bitter taste and sticky texture. The cork layer is pale gray or grayish-brown with deep irregular fissures. Microscopically, PAC exhibits characteristic stone cells, phloem fibers, and calcium oxalate crystals. While useful for rapid preliminary identification, morphological methods alone cannot reliably distinguish PAC from PCC when samples have been processed into decoction pieces [155,156].

#### 10.1.2. Thin-Layer Chromatography (TLC)

TLC is a simple, rapid, and cost-effective method recommended by the Chinese Pharmacopoeia for the preliminary identification of PAC. Using developing solvent systems such as ethyl acetate:butanone:formic acid:water (10:6:1:1) or petroleum ether:ethyl acetate (1:1), characteristic fluorescent spots of berberine, palmatine, and other alkaloids can be visualized under 365 nm UV light. By comparing the positions and fluorescence intensities with reference standards, PAC can be distinguished from PCC, with the latter typically showing higher berberine content and different spot intensity ratios [157,158].

#### 10.1.3. DNA Barcoding

DNA barcoding represents a modern, genetically based authentication method that is particularly valuable for distinguishing closely related species. By extracting genomic DNA from PAC samples, amplifying specific barcode regions (e.g., internal transcribed spacer, psbA-trnH, rbcL), and sequencing the amplicons, species-level identification can be achieved with high precision. Studies have identified species-specific single nucleotide polymorphism sites-such as at internal transcribed spacer position 173 and psbA-trnH positions 206 and 397-that reliably differentiate *P. amurense* from *P. chinense* [159,160]. This technique is especially useful for detecting adulteration in powdered or highly processed PAC samples in which morphological features have been lost.

#### 10.1.4. Near-Infrared (NIR) Fingerprinting

NIR spectroscopy combined with pattern recognition algorithms offers a rapid, non-destructive, and cost-effective authentication method. By collecting NIR diffuse reflectance spectra of PAC samples from different geographical origins and comparing them with spectra of potential adulterants, cluster analysis can accurately classify authentic PAC, PAC from different sources, and adulterated samples. This method requires no complex sample pretreatment and has demonstrated 100% classification accuracy in distinguishing PAC from PCC [161].

#### 10.1.5. Ion Mobility Spectrometry (IMS) Coupled with Mass Spectrometry

IMS separates gas-phase ions based on their charge, size, and shape, providing an additional dimension of separation when coupled with mass spectrometry (MS). This technique offers increased peak capacity and provides ion drift times and collision cross-sectional areas that aid in compound identification. Quantitative structure–retention relationship models can further predict the retention times of structurally analogous compounds, assisting in the structural elucidation of unknown components [162,163]. While more sophisticated than TLC or NIR, IMS-MS is valuable for in-depth chemical profiling of PAC.

#### 10.1.6. HPLC Fingerprinting

HPLC fingerprinting is the most widely used analytical method for comprehensive PAC quality assessment. A recent study established an HPLC-diode array detector multi-wavelength detection method for the simultaneous determination of multiple component classes in PAC, including phenolic acids (3-*O*-feruloylquinic acid, 4-*O*-feruloylquinic acid, syringin), alkaloids (magnoflorine, phellodendrine, jatrorrhizine, palmatine, berberine), and limonoids (obaculactone, obacunone) [39]. Using optimized conditions (ultrasonic extraction with 70% methanol, a material-to-liquid ratio of 1:40, three extractions of 20 min each; Waters XBridge C_18_ column with acetonitrile-ammonium bicarbonate gradient elution; detection at 215/275/280/310 nm), the method achieved excellent linearity (R^2^ > 0.9984) and recovery (98.94–100.57%). When applied to 33 batches of PAC from different regions, the results revealed a clear geographical trend: phenolic acid and alkaloid contents followed a “higher in the south, lower in the north” pattern, while limonoids showed the opposite trend. Combined with cluster analysis, this method effectively classified PAC samples by geographical origin [39].

### 10.2. Marker Compounds for Quality Assessment

The selection of appropriate marker compounds is fundamental to PAC quality control. Based on current phytochemical and pharmacological knowledge, three categories of markers are commonly used.

#### 10.2.1. Alkaloid Markers

Alkaloids are the most extensively studied and pharmacologically significant components of PAC. The five key alkaloid markers are as follows: (1) Berberine: The most abundant alkaloid in PAC (typically 1–6% by dry weight) and the primary efficacy marker specified in the Chinese Pharmacopoeia. Berberine exhibits broad-spectrum antibacterial, anti-inflammatory, hypoglycemic, and anticancer activities. (2) Palmatine: The second most abundant alkaloid (typically 0.5–3%), with anti-inflammatory, anti-gout, and neuroprotective effects. (3) Jatrorrhizine: Present in moderate amounts (0.1–1%), contributing to anti-inflammatory and antioxidant activities. (4) Phellodendrine: A characteristic alkaloid of *Phellodendron* species, with immunosuppressive and anti-inflammatory properties. (5) Magnoflorine: An aporphine-type alkaloid with anti-inflammatory, antifungal, and neuroprotective effects.

The ratios of these alkaloids-particularly the berberine-to-palmatine and berberine-to-phellodendrine ratios-can help distinguish PAC from PCC, as PCC typically contains higher berberine and lower phellodendrine levels compared to PAC [39].

#### 10.2.2. Phenolic Acid Markers

Phenolic acids, including 3-*O*-feruloylquinic acid and 4-*O*-feruloylquinic acid, contribute to the antioxidant and anti-inflammatory activities of PAC. These compounds are not present in all *Phellodendron* species and may serve as complementary markers for species differentiation [39,63].

#### 10.2.3. Limonoid Markers

Limonoids such as obacunone and obaculactone exhibit anti-inflammatory, anticancer, and insecticidal activities. While present at lower concentrations than alkaloids, limonoids provide additional chemical information for comprehensive quality assessment and have shown distinct geographical distribution patterns [39].

### 10.3. Pharmacopoeial Standards for PAC

The Chinese Pharmacopoeia (2025 edition) specifies the following quality standards for PAC [3]: (1) Identification: TLC identification using obacunone as a reference standard and PAC reference herb, with petroleum ether (60–90 °C)-ethyl acetate (1:1) as the mobile phase, visualized by spraying with 10% sulfuric acid ethanol solution and heating at 105 °C. (2) Content determination: HPLC quantification of berberine hydrochloride (not less than 0.60%) and palmatine hydrochloride (not less than 0.30%), calculated on a dried basis. (3) Loss on drying (moisture content): Not more than 11.0%. (4) Total ash: Not more than 9.0%. (5) Acid-insoluble ash: Not specified in the current monograph. (6) Extractive content: Ethanol-soluble extractives (60% ethanol, hot extraction method) not less than 17.0%.

It is noteworthy that the current pharmacopoeial standard relies almost exclusively on berberine content as the sole quantitative marker. While berberine is indeed a major active component, this single-marker approach does not fully capture the overall chemical complexity of PAC, nor does it effectively distinguish PAC from PCC (both contain berberine, albeit at different levels).

### 10.4. Current Analytical Challenges and Future Directions

Despite the availability of various analytical methods, several challenges remain in ensuring consistent PAC quality. Due to considerable batch-to-batch variation—where berberine content can range from <3% to >6% depending on geographical origin, harvest time, and storage conditions [39]—and the persistent difficulty of differentiating PAC from PCC (which requires quantitative multi-component analysis, as their chemical profiles differ only in compound ratios), the current pharmacopoeial standard relying on berberine as the sole quantitative marker has several limitations: it does not account for other bioactive alkaloids, cannot detect adulteration with PCC or other *Phellodendron* species, and ignores potential synergistic effects. Therefore, holistic fingerprint-based approaches (e.g., HPLC, NIR, or HPLC-MS based metabolomics) are more appropriate; however, their adoption is limited by the lack of standardized reference fingerprints and computational complexity. Furthermore, the incomplete understanding of the relationship between chemical markers and therapeutic efficacy remains a fundamental challenge. Future efforts should prioritize pharmacological activity-based quality control that integrates bioactivity assays with chemical analysis to ensure that standards reflect therapeutic potential rather than merely chemical abundance.

## 11. Discussion

### 11.1. Protected Status Disparity: PAC vs. PCC

Both PAC and PCC are officially recorded medicinal materials in the Chinese Pharmacopoeia. However, there is a notable difference in their wild plant protection statuses. This discrepancy does not stem from the stipulations of the pharmacopoeia but is rather the result of the independent application of two distinct evaluation systems—the drug quality standard system and the species endangerment assessment system.

PAC is listed as a National Second-Class Key Protected Wild Plant, primarily because its wild populations have long suffered from over-harvesting, leading to a severe survival crisis. The ecological reality of declining population size, weak natural regeneration capacity, and continuously decreasing numbers directly justifies its legally protected status. In contrast, although the wild resources of PAC also face certain pressures, their current endangerment level has not yet met the criteria for inclusion in the List of National Key Protected Wild Plants. Thus, the function of the Chinese Pharmacopoeia is to standardize medicinal quality and efficacy, while the designation of the National Key Protected Wild Plants List is based on a scientific assessment of the current status of wild species resources. The protected status accorded to PAC essentially reflects the imminent risk of extinction faced by its wild populations and represents a necessary legal measure taken by the state to implement emergency conservation for this valuable medicinal resource.

### 11.2. Challenges and Strategic Responses in the Transition from Cork Tree Bark to Sprouts as Raw Material

#### 11.2.1. Challenges

The traditional medicinal part of the cork tree (*P. amurense*) is its bark, which generally requires 10–15 years of cultivation before it can be harvested. In contrast, using sprouts (tender branches) as raw material allows for harvesting within the same year and offers higher yield. Sprouts are characterized by sustainable harvesting, which reduces the enterprise’s inventory turnover period from approximately 10 years to 1–2 years, significantly lowering capital occupation costs.

The main challenges currently faced are as follows. First, the content of active ingredients in sprouts is insufficient, with alkaloids such as berberine being lower than in the bark; without improvements in extraction efficiency or concentration processes, the efficacy of the final product may be affected. Second, the quality of sprouts varies considerably due to fluctuations in active ingredient content, which are influenced by growing season, soil conditions, and harvesting time. These factors contribute to significant batch-to-batch differences, necessitating the establishment of corresponding fingerprint profiles and content control standards. Third, regulatory approval presents difficulties, as the current Chinese Pharmacopoeia only includes bark as the medicinal source for PAC. Including sprouts in the pharmacopoeia or applying for their approval as a new medicinal material would require completing clinical equivalence studies and safety evaluations. Fourth, harvesting sprouts may impact the tree itself, as frequent or improper harvesting can inhibit tree growth, potentially leading to branch withering and affecting long-term sustainable harvesting. Fifth, market acceptance is limited, as pharmaceutical enterprises and end-users have insufficient awareness of medicinal materials from “non-traditional parts,” requiring market confidence to be gradually built through public education and clinical validation. Finally, the supply chain is not yet mature, as the current market trading, storage, and transportation systems are primarily established around bark. Sprouts lack established circulation channels, packaging standards, and logistics specifications, leading to increased procurement costs and transportation losses.

#### 11.2.2. Solutions

The pathways to address these challenges include the following. First, establish a quality standard system by setting content standards for active ingredients in sprouts and develop characteristic fingerprint profiles to ensure consistent quality between batches of raw materials. Second, optimize extraction processes by developing efficient purification technologies such as ultrasonic extraction and microwave-assisted extraction to compensate for the initially lower content in sprouts and to ensure efficacy equivalent to that of bark. Third, conduct clinical equivalence studies to verify, through in vitro and in vivo efficacy comparisons and small-scale clinical trials, that sprout extracts are equivalent to traditional bark in functions such as clearing heat, drying dampness, purging fire, and detoxifying. Fourth, formulate sustainable harvesting protocols by establishing annual harvesting quotas for sprouts and scientific pruning procedures to minimize long-term impacts on tree growth. Fifth, promote the development of supporting industrial chain infrastructure by gradually establishing specialized trading platforms, packaging standards, storage and transportation specifications, and quality traceability systems for sprouts to reduce costs and losses in circulation.

### 11.3. Other Substitutes for PAC

#### 11.3.1. Interspecific Substitution

There are approximately 4 species of plants in the genus *Phellodendron worldwide*, with 2 species *(P. amurense* and *P. chinense*) and one variety (*P. chinense* var. glabriusculum) distributed in China. In medicinal practice, besides PAC, PCC (sourced from *P. chinense* and its variety) has been widely used as an alternative resource. The primary pharmacological activity of Huangbai originates from its rich isoquinoline alkaloids, particularly key components such as berberine, palmatine, and jatrorrhizine. Both PAC and PCC contain these alkaloids, which forms the chemical basis for their similar efficacy and provides scientific support for their joint inclusion as official medicinal materials in the Chinese Pharmacopoeia. Consequently, substituting PAC with PCC has become a practical and viable strategy to address the resource crisis of PAC.

It should be noted that although the two types of Huangbai exhibit a high degree of overlap in chemical composition, differences still exist in the content of their main active ingredients. While their broad-spectrum efficacy in “clearing heat and drying dampness” is similar, these differences may lead to subtle variations in the intensity or direction of certain specific pharmacological effects. For instance, PCC may have an advantage in applications where berberine serves as the primary efficacy indicator. Such distinctions should be appropriately considered in clinical medication and formulation development. Overall, substituting PAC with PCC is currently the most mature and lowest-risk coping strategy. This approach is supported by regulatory foundations, chemical structural similarities, and a long-standing consensus in clinical practice. Even though the two differ in alkaloid proportions, this does not affect their status as equivalent medicinal materials within the pharmacopoeial framework.

#### 11.3.2. Organ Substitution

Utilizing non-traditional medicinal parts of *P. amurense*, such as sprouts, has emerged as a potential ideal alternative resource due to their strong regenerative capacity and minimal damage to the parent plant during harvesting. Theoretically, different plant organs may synthesize and accumulate similar secondary metabolites. Taking bayberry as an example: its stem bark is widely used in traditional medicine, but over-harvesting has threatened the survival of this species. Research indicates that although the total phenolic and flavonoid contents in its twigs are slightly lower than those in the stem bark, their high-performance TLC fingerprint profiles are highly similar, suggesting that they share fundamentally consistent chemical constituent groups. This demonstrates that twigs are chemically highly comparable to stem bark and can serve as a sustainable alternative resource, helping to alleviate resource pressure on the plant. However, chemical equivalence is the foundation for achieving the substitution of medicinal parts. In the absence of reliable data confirming whether the active ingredient content in *P. amurense* sprouts approximates or reaches the level found in the bark, their substitution potential remains largely theoretical and requires further clarification through systematic compositional analysis and efficacy validation.

#### 11.3.3. Bioengineering

Accumulation of medicinal constituents in mature *P. amurense* occurs predominantly within the phloem, where the root phloem exhibits the highest content, followed by the basal trunk phloem. In young trees, medicinal components are also mainly enriched in the phloem, though the basal trunk phloem exhibits the highest concentration, followed by the root phloem. Generally, larger root diameters correlate with higher medicinal component content. Studies indicate that the berberine content in roots can be 7–10 times higher than that in stems.

Utilizing hairy root culture as an alternative to wild resources represents a significant direction for the sustainable use of TCM resources. *P. amurense* hairy roots are characterized by rapid growth, high branching, hormone autotrophy, genetic stability, and strong secondary metabolite synthesis capabilities. These features make them suitable for large-scale cultivation, offering a promising approach to alleviating pressure on wild resources and advancing the modernization of TCM.

The primary pharmacological activities of PAC stem from its various isoquinoline alkaloids, such as berberine, palmatine, jatrorrhizine, and phellodendrine. Currently, progress in bioengineering technologies for these components varies: Berberine has been produced via microbial fermentation and plant tissue culture based on yeast or Escherichia coli, with relatively mature technology. Palmatine has established mature total chemical synthesis routes, but its biosynthesis remains exploratory, requiring further development of specific methyltransferases or modification of existing berberine synthesis platforms. Jatrorrhizine primarily relies on plant cell or hairy root culture for production, as microbial synthesis pathways have yet to be realized, necessitating deeper enzymatic studies. In summary, current bioengineering research remains focused on the core metabolic pathway of berberine, while the large-scale production of other alkaloids still largely depends on traditional extraction or chemical synthesis. Biosynthetic technologies for alternative pathways require further breakthroughs.

### 11.4. Improvements

#### 11.4.1. Comparative Analysis of Chemical Components

Conduct strictly controlled experiments to systematically compare the chemical compositions of PAC and sprouts at different growth stages. The focus should be on the quantitative analysis of key alkaloids such as berberine, palmatine, phellodendrine, and magnoflorine to clarify the similarities and differences in their material basis.

#### 11.4.2. Optimization of Harvesting Standards

Investigate the dynamic changes in the content of active components in sprouts of different growth years to determine the optimal harvesting period, thereby ensuring the stability of medicinal quality and the enrichment level of effective components. Simultaneously, systematically study the effects of cultivation conditions such as nitrogen forms and light intensity on the accumulation of active components in sprouts to provide a scientific basis for large-scale and standardized cultivation.

#### 11.4.3. Parallel Validation of Pharmacological Activity

##### Comparative Study of In Vitro Efficacy

Prepared separately, standardized sprout extracts and bark extracts will be systematically compared for their activity differences under identical experimental conditions across three primary pharmacodynamic indicators: antibacterial activity, assessed via the microdilution method to determine the MIC against common pathogens such as *Staphylococcus aureus* and *Escherichia coli*; anti-inflammatory activity, evaluated based on the lipopolysaccharide-induced RAW 264.7 macrophage model by detecting the inhibition of inflammatory factors such as NO, TNF-*α*, and IL-6; and hypoglycemic activity, examined through in vitro *α*-glucosidase inhibition assays and pancreatic islet cell protection models.

##### Validation of In Vivo Efficacy Equivalence

If in vitro activities prove similar, subsequent animal in vivo pharmacodynamic equivalence studies should be conducted. Specifically, anti-inflammatory equivalence can be assessed using the xylene-induced mouse ear swelling model or the carrageenan-induced rat paw swelling model, while hypoglycemic equivalence can be evaluated in streptozotocin-induced diabetic rats or db/db spontaneous diabetic mouse models. By comparing the pharmacodynamic responses of sprout extracts and bark extracts at equivalent doses, this phase aims to preliminarily determine whether the two materials exhibit bioequivalence in vivo.

### 11.5. A Note on Distinguishing PAC from PCC in Literature Interpretation

As noted in the Introduction, PAC and PCC were historically not distinguished and were collectively referred to as “Huangbai” in ancient texts and older literature. It was not until the 2005 edition of the Chinese Pharmacopoeia that they began to be listed as two independent medicinal materials. Consequently, when reviewing phytochemical, pharmacological, and quality control evidence from earlier decades, one must exercise caution in attributing findings specifically to PAC.

In this review, we have adopted a rigorous approach to address this issue. For each cited study, we verified the botanical origin of the plant material. Only studies that explicitly identified the plant as *P. amurense* (or its bark) were included as evidence for PAC. Studies using *P. chinense*, unspecified *Phellodendron* species, or simply the generic term “Huangbai” without clear botanical authentication were excluded, unless they specifically compared the two species. In the latter case, the distinction is clearly indicated in the text.

This methodological transparency is essential for ensuring that the updated and comprehensive review of PAC presented here is both accurate and academically rigorous. Future studies on PAC should continue to adhere to clear botanical identification to avoid the historical ambiguity that has long surrounded this valuable medicinal material.

### 11.6. Connecting Conservation Status to Utilization and Quality Control

As noted in the section on Geographical Distribution, *P. amurense* has been listed as a National Second-Class Key Protected Plant in China since 1999 and assessed as Vulnerable in the China Species Red List due to over-harvesting, deforestation, and natural disasters [13,14]. While this conservation status is mentioned earlier in the manuscript, its implications for future medicinal use, raw material availability, and quality consistency have not been explicitly connected. This section addresses that gap.

#### 11.6.1. From Conservation Status to Raw Material Availability

The protected status of *P. amurense* has direct consequences for the PAC supply chain. As a protected species, wild *P. amurense* cannot be legally harvested without special permits, shifting the source from wild-harvested to cultivated material over the past two decades. However, cultivated trees are typically harvested earlier (10–15 years) than wild trees (20–30+ years), potentially altering alkaloid content–particularly berberine—and affecting efficacy and quality control. Additionally, the long cultivation cycle prevents rapid supply responses to increased demand, creating upward price pressure and potentially incentivizing adulteration or the use of non-traditional plant parts. Thus, the conservation status of *P. amurense* is a fundamental determinant of PAC availability, pricing, and raw material characteristics.

#### 11.6.2. From Raw Material Availability to Quality Consistency

Changes in raw material sources directly affect PAC quality consistency. Regarding wild versus cultivated PAC, a critical question is whether quality standards should be adjusted for cultivated material or whether cultivation practices should be optimized to match wild material. The current Chinese pharmacopoeial standard (berberine hydrochloride ≥ 0.60%) [3] was likely based on wild material, yet some cultivated batches may struggle to meet this threshold. Sprouts have been proposed as a sustainable bark substitute as they can be harvested without killing the tree, but their lower alkaloid content-particularly berberine-raises concerns about meeting current standards. If sprouts are to be used legitimately, sprout-specific quality standards or processing methods to raise alkaloid levels are needed, along with robust traceability methods to prevent undisclosed substitution. Root bark offers 7–10 times higher berberine content than stem bark but kills the tree, making it less sustainable than sprouts. Thus, a trade-off exists: root bark provides higher quality but lower sustainability, while sprouts offer higher sustainability but potentially lower quality. Quality and sustainability are not always aligned, and decision-making depends on whether the priority is maximizing potency or ensuring long-term resource availability.

#### 11.6.3. From Quality Consistency to Long-Term Sustainability

Quality control practices can also influence sustainability. High thresholds for marker compounds such as berberine may incentivize the harvesting of older or wild trees and the use of root bark-all of which undermine sustainability. Conversely, low thresholds may permit ineffective material. Thus, quality standards should be evidence-based, reflecting the minimum content needed for efficacy, rather than the content of the best wild material, so that cultivated and sustainable sources can comply. Bioengineering offers an alternative: hairy root culture and fermentation can produce berberine without harvesting plants. While technically feasible, this approach is not yet commercially competitive. Additionally, bioengineered alkaloids lack other PAC constituents (e.g., polysaccharides, phenolic acids), raising questions about whether they qualify as “PAC.” Substituting bioengineered berberine for PAC extract in medicines would represent a fundamental change requiring regulatory review.

#### 11.6.4. Future Research Priorities at the Sustainability–Quality Interface

To address the connections among conservation, utilization, and quality control, several research priorities are identified. These include comparative efficacy studies of wild versus cultivated PAC to determine therapeutic equivalence; establishment of minimum efficacy thresholds for key alkaloids (berberine, palmatine, phellodendrine) to guide rational quality standards; systematic sprout-to-bark equivalence studies to assess sprouts as a true substitute; cultivation optimization to maximize alkaloid content in cultivated PAC; development of cost-effective traceability technologies (e.g., NIR fingerprinting, stable isotope analysis) to determine PAC source; and a life cycle assessment comparing the environmental impacts of wild harvesting, cultivated bark, sprout production, and bioengineered alkaloid production to inform sustainability policy.

#### 11.6.5. Summary: The Conservation–Utilization–Quality Triangle

The relationship among conservation status, medicinal utilization, and quality control is triangular: each vertex influences the other two. Conservation restricts wild harvesting, shifting supply to cultivated and alternative sources. Utilization changes-such as switching from wild to cultivated material or from bark to sprouts-alter chemical profiles, affecting compliance with existing quality standards. Conversely, quality standards that are set too high incentivize unsustainable harvesting practices (e.g., older trees, root bark, wild populations). Therefore, a holistic approach must consider all three vertices simultaneously. Future revisions of the Chinese Pharmacopoeia should consider resource sustainability alongside analytical chemistry and pharmacology, while conservation policies should consider the quality implications of restricting access to wild material without providing viable alternatives.

## 12. Conclusions

PAC is a valuable traditional medicine with diverse pharmacological activities. While approximately 170 compounds have been identified, future research should prioritize several specific directions. First, authenticated source tracing using DNA barcoding and comparative chemical profiling is urgently needed to distinguish PAC from PCC and ensure raw material integrity. Second, quality standards must move beyond single-marker (berberine) quantification to incorporate multi-component fingerprinting that reflects therapeutic potential. Third, pharmacokinetic studies are required to elucidate the absorption and metabolism of key alkaloids. Fourth, well-designed clinical trials are necessary to validate its efficacy in conditions such as gout and chronic prostatitis. Addressing these gaps will transform PAC from ethnobotanical use to evidence-based application, while integrating sustainable cultivation practices to protect vulnerable wild resources.

## Figures and Tables

**Table 1:** Alkaloids isolated or identified from *Phellodendron amurense* Rupr.

No.	Name	Formula	Exact Theoretical Molecular Weight	Collection Site/ Material Sources	Characterization Method	Ref.
1.	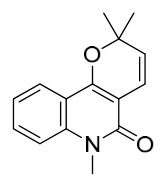 *N*-Methylflindersine	C_15_H_15_NO_2_	241.1103	Tongrentang Pharmacy Co., Ltd. Shenyang, Liaoning Province, China	HPLC-MS, MS-DIAL, Cytoscape analysis (version 3.9.1.)	[28]
2.	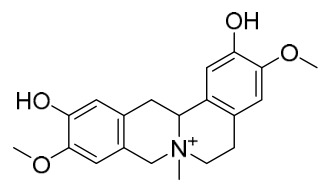 Phellodendrine	C_20_H_24_NO_4_^+^	342.1700	Tongrentang Pharmacy Co., Ltd. Shenyang, Liaoning Province, China	HPLC-MS, MS-DIAL, Cytoscape analysis	[28]
3.	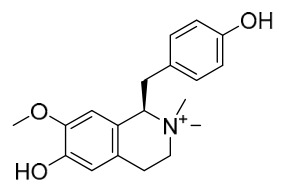 Lotusine	C_19_H_24_NO_3_^+^	314.1751	Tongrentang Pharmacy Co., Ltd. Shenyang, Liaoning Province, China	HPLC-MS, MS-DIAL, Cytoscape analysis	[28]
4.	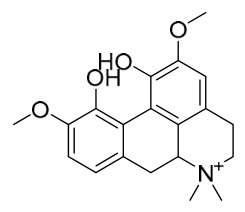 Magnoflorine	C_20_H_24_NO_4_^+^	342.1700	Tongrentang Pharmacy Co., Ltd. Shenyang, Liaoning Province, China	HPLC-MS, MS-DIAL, Cytoscape analysis	[28]
5.	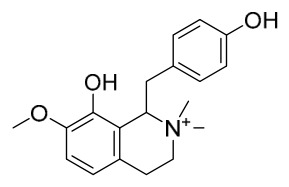 Oblongine	C_19_H_24_NO_3_^+^	314.1751	Tongrentang Pharmacy Co., Ltd. Shenyang, Liaoning Province, China	HPLC-MS, MS-DIAL, Cytoscape Analysis	[28]
6.	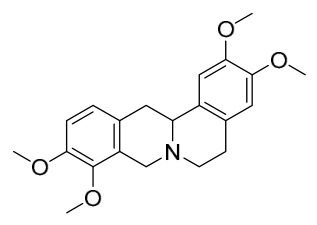 Tetrahydropalmatine	C_21_H_25_NO_4_	355.1784	Tongrentang Pharmacy Co., Ltd. Shenyang, Liaoning Province, China	HPLC-MS, MS-DIAL, Cytoscape analysis	[28]
7.	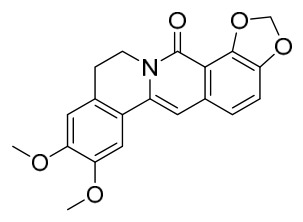 Oxyepiberberine	C_20_H_17_NO_5_	351.1107	Tongrentang Pharmacy Co., Ltd. Shenyang, Liaoning Province, China	HPLC-MS, MS-DIAL, Cytoscape analysis	[28]
8.	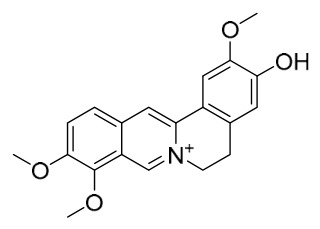 Jatrorrhizine	C_20_H_20_NO_4_^+^	338.1387	Tongrentang Pharmacy Co., Ltd. Shenyang, Liaoning Province, China	HPLC-MS, MS-DIAL, Cytoscape analysis	[28]
9.	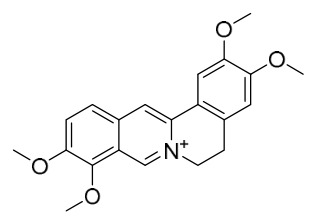 Palmatine	C_21_H_22_NO_4_^+^	352.1543	Tongrentang Pharmacy Co., Ltd. Shenyang, Liaoning Province, China	HPLC-MS, MS-DIAL, Cytoscape analysis	[28]
10.	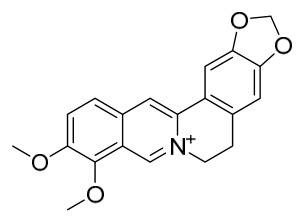 Berberine	C_20_H_18_NO_4_^+^	336.1230	Tongrentang Pharmacy Co., Ltd. Shenyang, Liaoning Province, China	HPLC-MS, MS-DIAL, Cytoscape analysis	[28]
11.	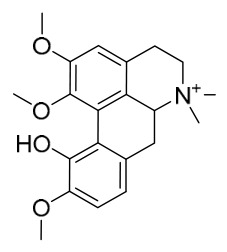 Menisperine	C_21_H_26_NO_4_^+^	356.1856	Tongrentang Pharmacy Co., Ltd. Shenyang, Liaoning Province, China	HPLC-MS, MS-DIAL, Cytoscape analysis	[28]
12.	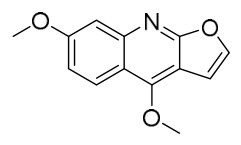 *γ*-Fagarine	C_13_H_11_NO_3_	229.0739	Kyungdong herbal market of Korea Seoul, South Korea	UV, IR, MS, ^1^H NMR, ^13^C NMR	[29]
13.	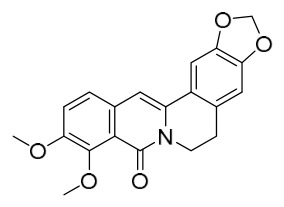 Oxyberberine	C_20_H_17_NO_5_	351.1107	Kyungdong herbal market of Korea Seoul, South Korea	UV, IR, MS, ^1^H NMR, ^13^C NMR	[29]
14.	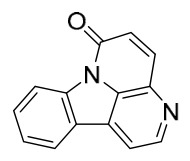 Canthin-6-one	C_14_H_8_N_2_O	220.0637	Kyungdong herbal market of Korea Seoul, South Korea	UV, IR, MS, ^1^H NMR, ^13^C NMR	[29]
15.	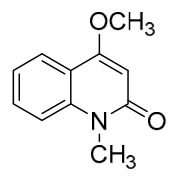 4-Methoxy-*N*-methyl-2-quinolone	C_11_H_11_NO_2_	189.0790	Kyungdong herbal market of Korea Seoul, South Korea	UV, IR, MS, ^1^H NMR, ^13^C NMR	[29]
16.	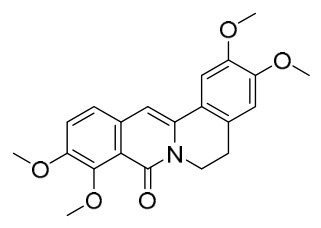 Oxypalmatine	C_21_H_21_NO_5_	367.1420	Kyungdong herbal market of Korea Seoul, South Korea	UV, IR, MS, ^1^H NMR, ^13^C NMR	[29]
17.	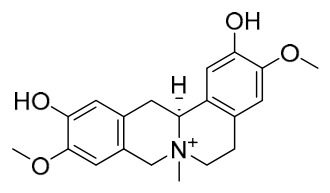 Columbamine	C_20_H_20_NO_4_^+^	342.1700	Liaoning, Hebei, Heilongjiang and Jilin Provinces of China	LC-ESI-MS/MS	[30]
18.	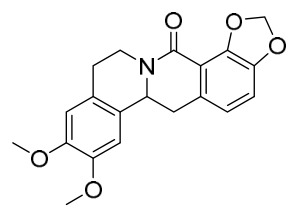 8-Oxoepiberberine	C_20_H_19_NO_5_	353.1263	Liaoning, Hebei, Heilongjiang and Jilin Provinces of China	LC-ESI-MS/MS	[30]
19.	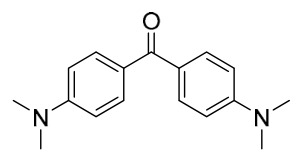 *Bis*-[4-(dimethylamino)phenyl]methanone	C_17_H_20_N_2_O	268.1576	Liaoning, Hebei, Heilongjiang and Jilin Provinces of China	LC-ESI-MS/MS	[30]
20.	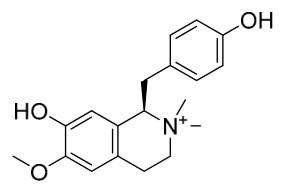 Magnocurarine	C_19_H_24_NO_3_^+^	314.1751	Liaoning, Hebei, Heilongjiang and Jilin Provinces of China	LC-ESI-MS/MS	[30]
21.	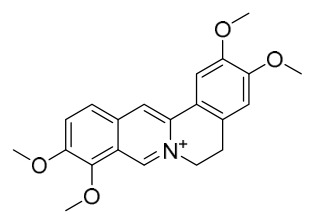 (+)-Tembetarine	C_21_H_22_NO_4_^+^	352.1543	Liaoning, Hebei, Heilongjiang and Jilin Provinces of China	LC-ESI-MS/MS	[30]
22.	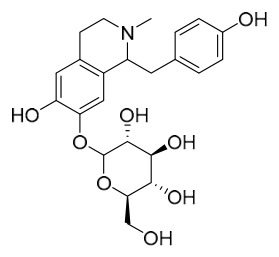 *N*-Methylhigenamine-7-*O*-glucopyranoside	C_23_H_29_NO_8_	447.1893	Liaoning, Hebei, Heilongjiang and Jilin Provinces of China	LC-ESI-MS/MS	[30]
23.	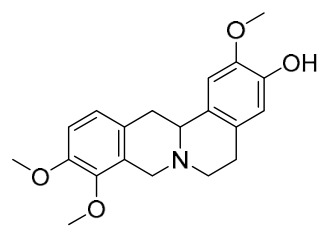 Tetrahydrojatrorrhizine	C_20_H_23_NO_4_	341.1627	Liaoning, Hebei, Heilongjiang and Jilin Provinces of China	LC-ESI-MS/MS	[30]
24.	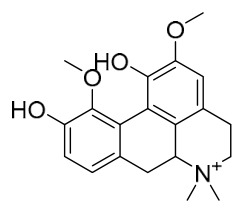 Isomer of magnoflorine	C_20_H_24_NO_4_^+^	342.1700	Liaoning, Hebei, Heilongjiang and Jilin Provinces of China	LC-ESI-MS/MS	[30]
25.	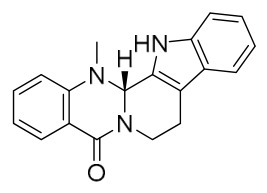 Evodiamine	C_19_H_17_N_3_O	303.1372	Liaoning, Hebei, Heilongjiang and Jilin Provinces of China	LC-ESI-MS/MS	[30]
26.	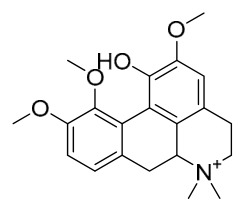 (+)-*N*-Methycorydaline	C_21_H_26_NO_4_^+^	356.1856	Liaoning, Hebei, Heilongjiang and Jilin Provinces of China	LC-ESI-MS/MS	[30]
27.	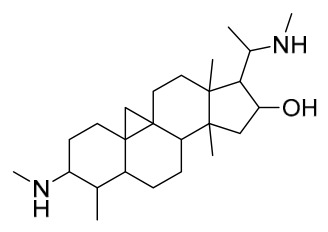 Dihydrocyclobuxine	C_25_H_44_N_2_O	388.3454	Liaoning, Hebei, Heilongjiang and Jilin Provinces of China	LC-ESI-MS/MS	[30]
28.	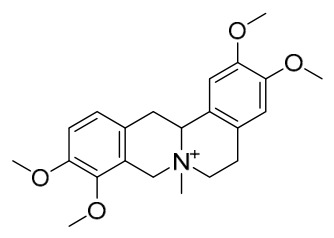 *N*-Methytetrahydropalmatine	C_22_H_28_NO_4_^+^	370.2013	Liaoning, Hebei, Heilongjiang and Jilin Provinces of China	LC-ESI-MS/MS	[30]
29.	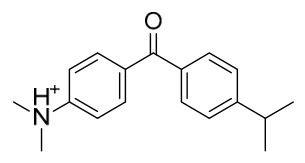 4-Dimethylamino-4′-isopropylbenzophenone	C_18_H_22_NO^+^	268.1696	Liaoning, Hebei, Heilongjiang and Jilin Provinces of China	LC-ESI-MS/MS	[30]
30.	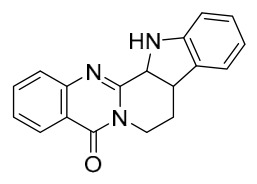 Rutaecarpine	C_18_H_15_N_3_O	289.1215	Liaoning, Hebei, Heilongjiang and Jilin Provinces of China	LC-ESI-MS/MS	[30]
31.	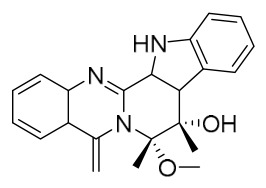 7-Hydroxy-8-methoxydihydrorutaecarpine	C_22_H_25_N_3_O_2_	363.1947	Liaoning, Hebei, Heilongjiang and Jilin Provinces of China	LC-ESI-MS/MS	[30]
32.	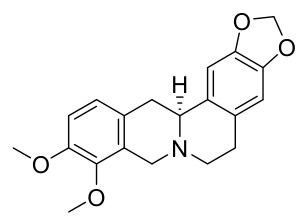 Tetrahydroberberine	C_20_H_21_NO_4_	339.1471	Liaoning, Hebei, Heilongjiang and Jilin Provinces of China	LC-ESI-MS/MS	[30]
33.	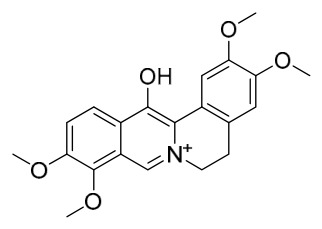 13-Hydroxypalmatine	C_21_H_22_NO_5_^+^	368.1492	Liaoning, Hebei, Heilongjiang and Jilin Provinces of China	LC-ESI-MS/MS	[30]
34.	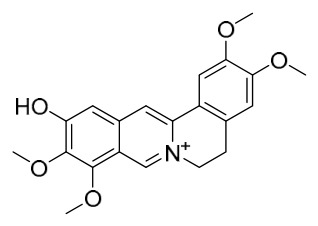 11-Hydroxypalmatine	C_21_H_22_NO_5_^+^	368.1492	Liaoning, Hebei, Heilongjiang and Jilin Provinces of China	LC-ESI-MS/MS	[30]
35.	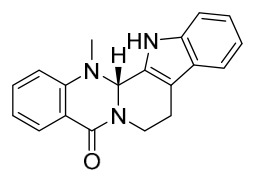 8-Oxopalmatine	C_21_H_22_NO_5_	368.1489	Liaoning, Hebei, Heilongjiang and Jilin Provinces of China	LC-ESI-MS/MS	[30]
36.	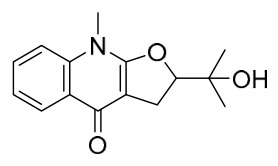 7,8-Dihydroxyrutaecarpine	C_15_H_17_NO_3_	259.1208	Liaoning, Hebei, Heilongjiang and Jilin Provinces of China	LC-ESI-MS/MS	[30]
37.	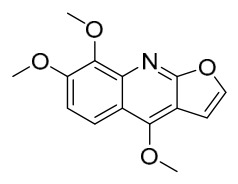 Skimmianine	C_14_H_13_NO_4_	259.0845	Liaoning, Hebei, Heilongjiang and Jilin Provinces of China	LC-ESI-MS/MS	[30]
38.	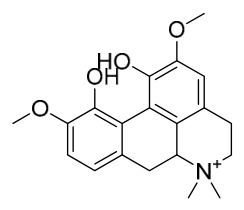 3,4-Dihydro-1-[(4-hydroxyphenyl)methyl]-7-methoxy-2-methyl-8-isoquinolinol	C_20_H_24_NO_4_^+^	342.1700	Liaoning, Hebei, Heilongjiang and Jilin Provinces of China	LC-ESI-MS/MS	[30]
39.	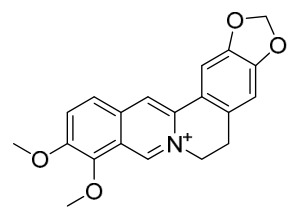 5,8,13,13a-Tetrahydro-2,9,10,11-tetrahydroxy-3-methoxy-7-methyl-6*H*-dibenzo[a,g]quinolizinium	C_20_H_18_NO_4_^+^	336.1230	Liaoning, Hebei, Heilongjiang and Jilin Provinces of China	LC-ESI-MS/MS	[30]
40.	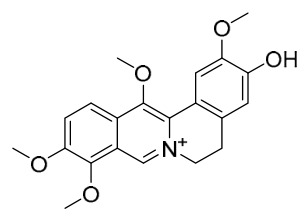 13-Methyljatrorrhizine	C_21_H_22_NO_5_^+^	368.1492	Liaoning, Hebei, Heilongjiang and Jilin Provinces of China	LC-ESI-MS/MS	[30]
41.	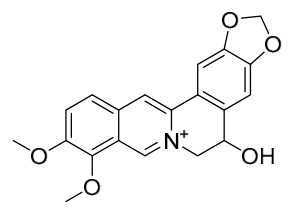 Berberastine	C_20_H_18_NO_5_^+^	352.1179	Shunwangcheng Herbal Medicine Market, Heze, Shandong Province, China	UHPLC-IM-Q-TOF-MS, compared with reference standards	[31]
42.	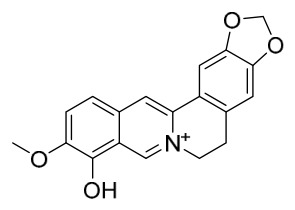 Berberrubine	C_19_H_16_NO_4_^+^	322.1074	Shunwangcheng Herbal Medicine Market, Heze, Shandong Province, China	UHPLC-IM-Q-TOF-MS, compared with reference standards	[31]
43.	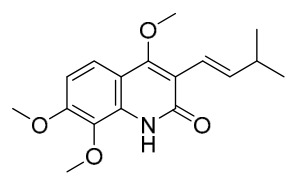 Dasycarpamin	C_17_H_21_NO_4_	303.1471	Shunwangcheng Herbal Medicine Market, Heze, Shandong Province, China	UHPLC-IM-Q-TOF-MS, compared with reference standards	[31]
44.	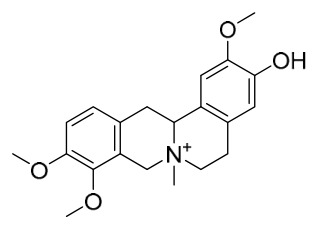 *N*-Methyltetrahydrojatrorrhizine	C_21_H_26_NO_4_^+^	356.1856	Shunwangcheng Herbal Medicine Market, Heze, Shandong Province, China	UHPLC-IM-Q-TOF-MS, compared with reference standards	[31]
45.	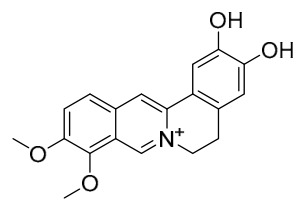 Demethyleneberberine	C_19_H_18_NO_4_^+^	324.1230	Shunwangcheng Herbal Medicine Market, Heze, Shandong Province, China	UHPLC-IM-Q-TOF-MS, compared with reference standards	[31]
46.	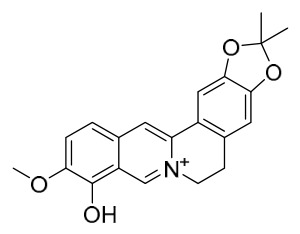 Thalifendine	C_21_H_20_NO_4_^+^	350.1387	Shunwangcheng Herbal Medicine Market, Heze, Shandong Province, China	UHPLC-IM-Q-TOF-MS, compared with reference standards	[31]
47.	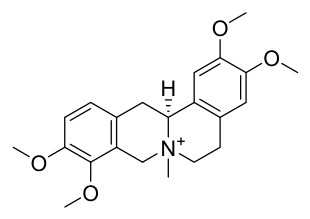 8-Methyltetrahydropalmatine	C_22_H_28_NO_4_^+^	370.2013	Shunwangcheng Herbal Medicine Market, Heze, Shandong Province, China	UHPLC-IM-Q-TOF-MS, compared with reference standards	[31]
48.	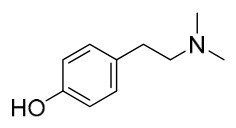 Candicine	C_10_H_15_NO	165.1154	Shunwangcheng Herbal Medicine Market, Heze, Shandong Province, China	UHPLC-IM-Q-TOF-MS, compared with reference standards	[31]
49.	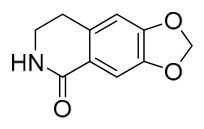 Noroxyhydrastinine	C_10_H_9_NO_3_	191.0582	Toho University herbal garden Funabashi-shi, Chiba, Japan	HR-ESI-MS, ^1^H NMR,^13^C NMR, ${\left[\alpha \right]}_{D}^{25}$	[32]
50.	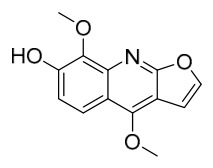 Haplopine	C_13_H_11_NO_4_	245.0688	Toho University herbal garden Funabashi-shi, Chiba, Japan	HR-ESI-MS, ^1^H NMR,^13^C NMR, ${\left[\alpha \right]}_{D}^{25}$	[32]
51.	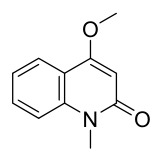 4-Methoxy-1-methylquinolin-2(1*H*)-one	C_11_H_11_NO_2_	189.0790	Toho University herbal garden Funabashi-shi, Chiba, Japan	HR-ESI-MS, ^1^H NMR,^13^C NMR, ${\left[\alpha \right]}_{D}^{25}$	[32]
52.	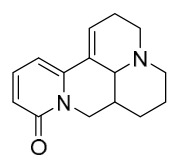 Δ^7^-Dehydrosophoramine	C_15_H_18_N_2_O	242.1419	Harbin Tongrentang Drug Store Harbin, Heilongjiang Province, China	UPLC-ESI-Q-TOF-MS	[33]
53.	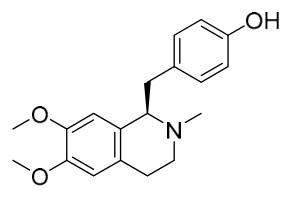 Armepavine	C_19_H_23_NO_3_	313.1678	Harbin Tongrentang Drug Store Harbin, Heilongjiang Province, China	UPLC-ESI-Q-TOF-MS	[34]
54.	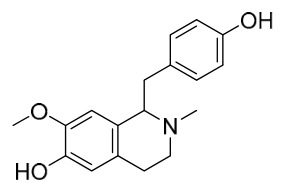 3,4-Dihydro-1-[(4-hydroxyphenyl)methyl]-7-methoxy-2-methyl-6-isoquinolinol	C_18_H_21_NO_3_	299.1521	Shunwangcheng Herbal Medicine Market, Heze, Shandong Province, China	UHPLC-IM-Q-TOF-MS, compared with reference standards	[31]
55.	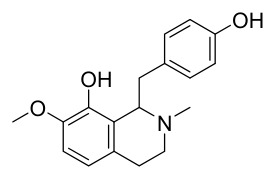 *N*-Demethyloblongine	C_18_H_21_NO_3_	299.1521	Shunwangcheng Herbal Medicine Market, Heze, Shandong Province, China	UHPLC-IM-Q-TOF-MS, compared with reference standards	[31]
56.	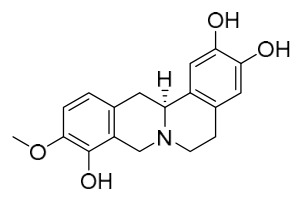 (*S*)-10-Methoxy-5,8,13,13atetrahydro-6*H*-isoquinolino[3,2-a]isoquinoline-2,3,9-triol	C_18_H_19_NO_4_	313.1314	Shunwangcheng Herbal Medicine Market, Heze, Shandong Province, China	UHPLC-IM-Q-TOF-MS, compared with reference standards	[31]
57.	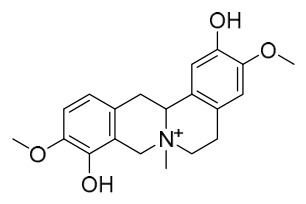 2,10-Dimethoxy-7-methyl-6,8,13,13a-tetrahydro-5*H*-isoquinolino[2,1-b]isoquinolin-7-ium-3,9-diol	C_20_H_24_NO_4_^+^	342.1700	Shunwangcheng Herbal Medicine Market, Heze, Shandong Province, China	UHPLC-IM-Q-TOF-MS, compared with reference standards	[31]
58.	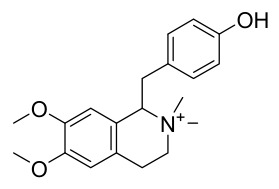 4-[(6,7-Dimethoxy-2,2-dimethyl-3,4-dihydro-1*H*-isoquinolin-2-ium-1-yl)methyl]phenol	C_20_H_26_NO_3_^+^	328.1907	Shunwangcheng Herbal Medicine Market, Heze, Shandong Province, China	UHPLC-IM-Q-TOF-MS, compared with reference standards	[31]
59.	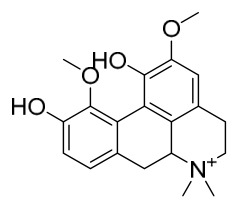 2,11-Dimethoxy-6,6-dimethyl-5,6,6a,7-tetrahydro-4*H*-dibenzo[de,g]quinolin-6-ium-1,10-diol	C_20_H_24_NO_4_^+^	342.1700	Shunwangcheng Herbal Medicine Market, Heze, Shandong Province, China	UHPLC-IM-Q-TOF-MS, compared with reference standards	[31]
60.	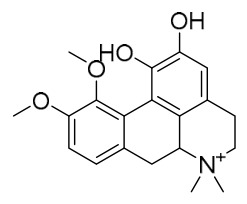 10,11-Dimethoxy-6,6-dimethyl-5,6,6a,7-tetrahydro-4*H*-dibenzo[de,g]quinolin-6-ium-1,2-diol	C_20_H_24_NO_4_^+^	342.1700	Shunwangcheng Herbal Medicine Market, Heze, Shandong Province, China	UHPLC-IM-Q-TOF-MS, compared with reference standards	[31]
61.	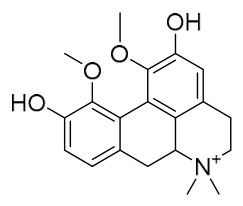 2,10-Dihydroxy-1,11-dimethoxy-6,6-dimethyl-5,6,6a,7-tetrahydro-4*H*-dibenzo[de,g]quinolin-6-ium	C_20_H_24_NO_4_^+^	342.1700	Shunwangcheng Herbal Medicine Market, Heze, Shandong Province, China	UHPLC-IM-Q-TOF-MS, compared with reference standards	[31]
62.	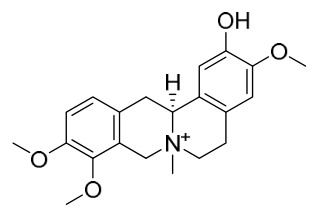 *N*-Methyltetrahydrocolumbamine	C_21_H_26_NO_4_^+^	356.1856	Shunwangcheng Herbal Medicine Market, Heze, Shandong Province, China	UHPLC-IM-Q-TOF-MS, compared with reference standards	[31]
63.	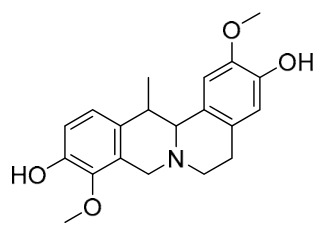 5,8,13,13a-Tetrahydro-2,9-dimethoxy-13-methyl-6*H*-dibenzo[a,g]quinolizine-3,10-diol	C_20_H_23_NO_4_	341.1627	Shunwangcheng Herbal Medicine Market, Heze, Shandong Province, China	UHPLC-IM-Q-TOF-MS, compared with reference standards	[31]
64.	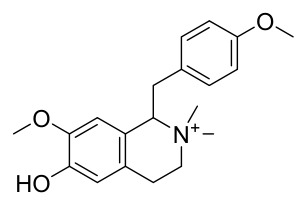 6-Hydroxy-7-methoxy-1-(4-methoxybenzyl)-2,2-dimethyl-1,2,3,4-tetrahydroisoquinolin-2-ium	C_20_H_26_NO_3_^+^	328.1907	Shunwangcheng Herbal Medicine Market, Heze, Shandong Province, China	UHPLC-IM-Q-TOF-MS, compared with reference standards	[31]
65.	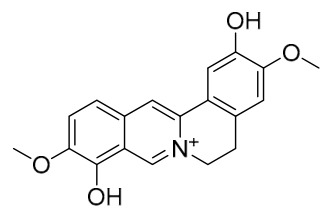 2,9-Dihydroxy-3,10-dimethoxy-5,6-dihydroisoquinolino[3,2-a]isoquinolin-7-ium	C_19_H_18_NO_4_^+^	324.1230	Shunwangcheng Herbal Medicine Market, Heze, Shandong Province, China	UHPLC-IM-Q-TOF-MS, compared with reference standards	[31]
66.	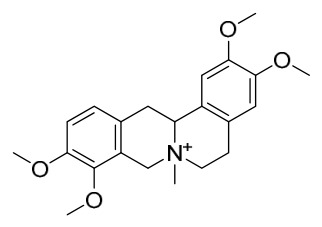 *N*-Methyltetrahydropalmatine	C_22_H_28_NO_4_^+^	370.2013	Shunwangcheng Herbal Medicine Market, Heze, Shandong Province, China	UHPLC-IM-Q-TOF-MS, compared with reference standards	[31]
67.	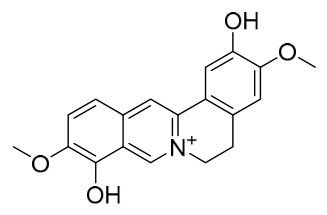 Dehydrocorydalmine	C_19_H_18_NO_4_^+^	324.1230	Shunwangcheng Herbal Medicine Market, Heze, Shandong Province, China	UHPLC-IM-Q-TOF-MS, compared with reference standards	[31]
68.	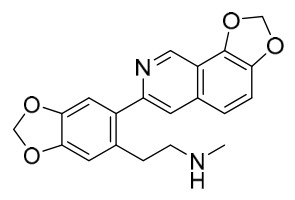 Corydamine	C_20_H_18_N_2_O_4_	350.1267	Shunwangcheng Herbal Medicine Market, Heze, Shandong Province, China	UHPLC-IM-Q-TOF-MS, compared with reference standards	[31]
69.	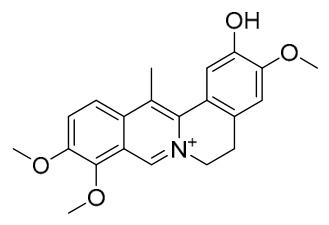 13-Methylcolumbamine	C_21_H_22_NO_4_^+^	352.1543	Shunwangcheng Herbal Medicine Market, Heze, Shandong Province, China	UHPLC-IM-Q-TOF-MS, compared with reference standards	[31]
70.	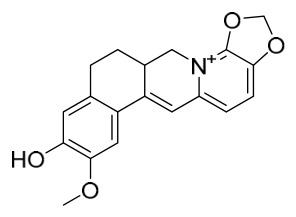 Groenlandicine	C_19_H_18_NO_4_^+^	324.1230	Shunwangcheng Herbal Medicine Market, Heze, Shandong Province, China	UHPLC-IM-Q-TOF-MS, compared with reference standards	[31]
71.	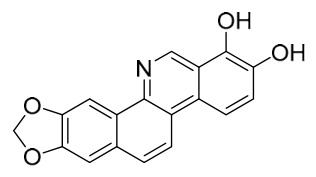 *O*-Demethyldecarine	C_18_H_11_NO_4_	305.0688	Shunwangcheng Herbal Medicine Market, Heze, Shandong Province, China	UHPLC-IM-Q-TOF-MS, compared with reference standards	[31]
72.	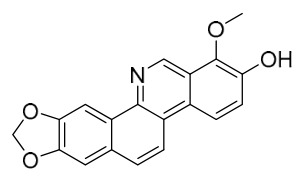 Decarine	C_19_H_13_NO_4_	319.0845	Shunwangcheng Herbal Medicine Market, Heze, Shandong Province, China	UHPLC-IM-Q-TOF-MS, compared with reference standards	[31]
73.	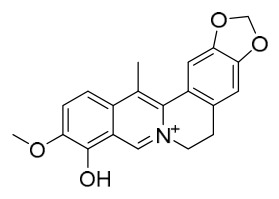 9-Hydroxy-10-methoxy-13-methyl-5,6-dihydro-[1,3]dioxolo[4,5-g]isoquinolino[3,2-a]isoquinolin-7-ium	C_20_H_18_NO_4_^+^	336.1230	Shunwangcheng Herbal Medicine Market, Heze, Shandong Province, China	UHPLC-IM-Q-TOF-MS, compared with reference standards	[31]
74.	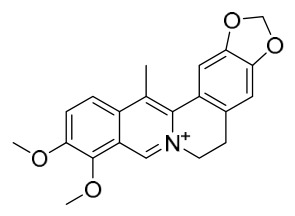 13-Methylberberine	C_21_H_20_NO_4_^+^	350.1387	Shunwangcheng Herbal Medicine Market, Heze, Shandong Province, China	UHPLC-IM-Q-TOF-MS, compared with reference standards	[31]
75.	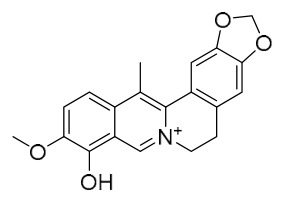 13-Methylberberrubine	C_20_H_18_NO_4_^+^	336.1230	Shunwangcheng Herbal Medicine Market, Heze, Shandong Province, China	UHPLC-IM-Q-TOF-MS, compared with reference standards	[31]
76.	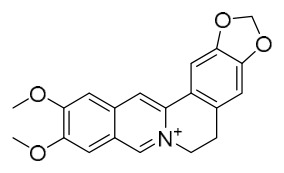 10,11-Dimethoxy-5,6-dihydro-[1,3]dioxolo[4,5-g]isoquinolino[3,2-a]isoquinolin-7-ium	C_20_H_18_NO_4_^+^	336.1230	Shunwangcheng Herbal Medicine Market, Heze, Shandong Province, China	UHPLC-IM-Q-TOF-MS, compared with reference standards	[31]
77.	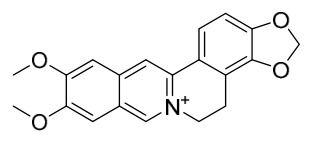 4,5-Dihydro-9,10-dimethoxybenzo[g]-1,3-benzodioxolo[5,4-a]quinolizinium	C_20_H_18_NO_4_^+^	336.1230	Shunwangcheng Herbal Medicine Market, Heze, Shandong Province, China	UHPLC-IM-Q-TOF-MS, compared with reference standards	[31]

UV: Ultraviolet spectrophotometry; IR: Infrared spectroscopy; HR-ESI-MS: High-resolution electrospray ionization mass spectrometry; MS: Mass spectrometry; ^13^C NMR: Carbon-13 nuclear magnetic resonance spectrometry; ^1^H NMR: Hydrogen-1 nuclear magnetic resonance spectrometry; HPLC-MS: High-performance liquid chromatography–mass spectrometry; MS-DIAL: Mass spectrometry-data independent analysis; LC-ESI-MS/MS: Liquid chromatography–electrospray ionization–tandem mass spectrometry/mass spectrometry; UPLC-ESI-Q-TOF-MS: Ultra-high-performance liquid chromatography–electrospray ionization–quadrupole time-of-flight–mass spectrometry; UHPLC-IM-Q-TOF-MS: Ultra-high-performance liquid chromatography–ion mobility–quadrupole time-of-flight–mass Spectrometry.

**Table 2:** Phenolics isolated or identified from *Phellodendron amurense* Rupr.

No.	Name	Formula	Exact Theoretical Molecular Weight	Collection Site/ Material Sources	Characterization Method	Ref.
78.	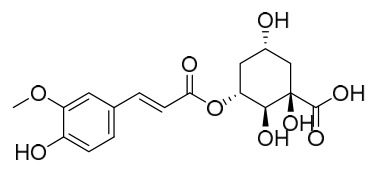 5-*O*-Feruloylquinic acid	C_17_H_20_O_9_	368.1107	Liaoning, Hebei, Heilongjiang and Jilin Provinces of China	LC-ESI-MS/MS	[30]
79.	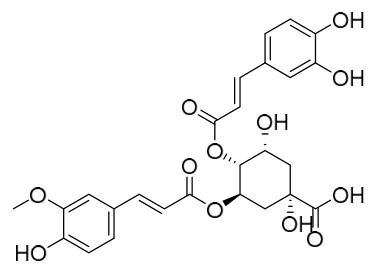 3-Feruoyl-4-caffeoylquinic acid	C_26_H_26_O_12_	530.1424	Liaoning, Hebei, Heilongjiang and Jilin Provinces of China	LC-ESI-MS/MS	[30]
80.	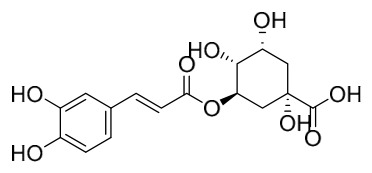 Chlorogenic acid	C_16_H_18_O_9_	354.0951	Liaoning, Hebei, Heilongjiang and Jilin Provinces of China	LC-ESI-MS/MS	[30]
81.	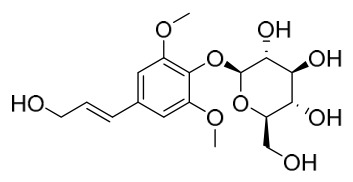 Syringin	C_17_H_24_O_9_	372.1420	Nagano Prefecture, Japan	^1^H NMR, ^13^C NMR	[35]
82.	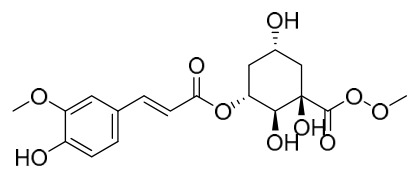 Methyl-5-*O*-feruloylquinate	C_18_H_22_O_10_	398.1213	Nagano Prefecture, Japan	^1^H NMR, ^13^C NMR	[35]
83.	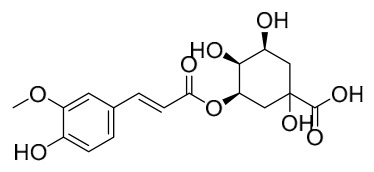 3-*O*-Fernloylquinic acid	C_17_H_20_O_9_	368.1107	Nagano Prefecture, Japan	^1^H NMR, ^13^C NMR	[35]
84.	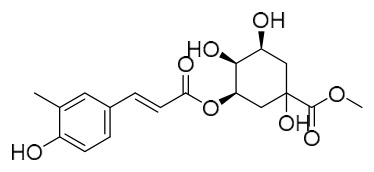 Methyl 3-*O*-fernloylquiniate	C_18_H_22_O_8_	366.1315	Nagano Prefecture, Japan	^1^H NMR, ^13^C NMR	[35]
85.	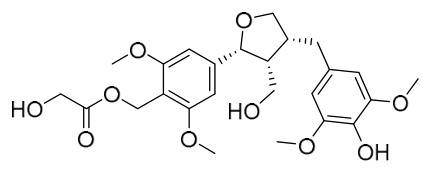 (±)-5,5′-Dimethoxylariciresinol 4-*O*-glucoside	C_25_H_32_O_10_	492.1995	Nagano Prefecture, Japan	^1^H NMR, ^13^C NMR	[35]
86.	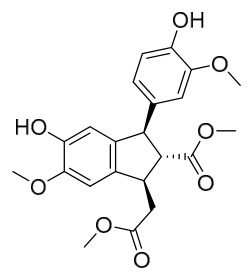 *γ*-Di(methylferulate)	C_22_H_24_O_8_	416.1471	Toho University herbal garden Funabashi-shi, Chiba, Japan	HR-ESI-MS, ^1^H NMR,^13^C NMR, ${\left[\alpha \right]}_{D}^{25}$	[32]
87.	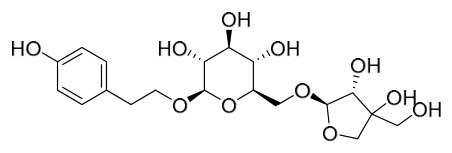 2-(*p*-Hydroxyphenyl)-ethanol 1-*O*-*β*-*D*-apiofuranosyl (1→6)-*β*-*D*-glucopyranoside	C_19_H_28_O_11_	432.1632	Nagano Prefecture, Japan	^1^H NMR, ^13^C-NMR	[35]
88.	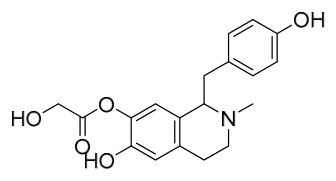 *N*-Methylhigenamine 7-*O*-*β*-*D*-glucopyranoside	C_19_H_21_NO_5_	343.1420	Nagano Prefecture, Japan	^1^H NMR, ^13^C-NMR	[35]
89.	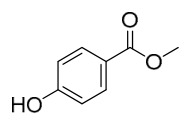 Methyl 4-hydroxybenzoate	C_8_H_8_O_3_	152.0473	Gyeongsangnam-do Southern Forest Research Center, Jinju, Korea	UV, IR, ^1^H NMR, ^13^C NMR	[36]
90.	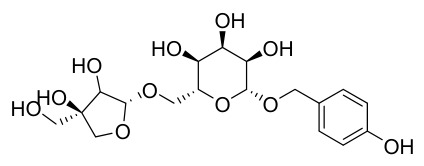 Osmanthuside H	C_18_H_26_O_11_	418.1475	Naemome Dah, Ulsan, Korea	UV, ESI-MS, ^1^H NMR, ^13^C NMR	[37]
91.	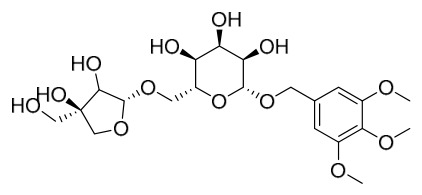 Kelampayoside A	C_21_H_32_O_13_	492.1843	Naemome Dah, Ulsan, Korea	UV, ESI-MS, ^1^H NMR, ^13^C NMR	[37]
92.	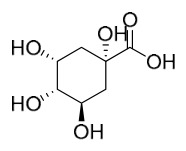 Quinic acid	C_7_H_12_O_6_	192.0634	Harbin Tongrentang Drug Store Harbin, Heilongjiang Province, China	UPLC-ESI-Q-TOF-MS	[33]
93.	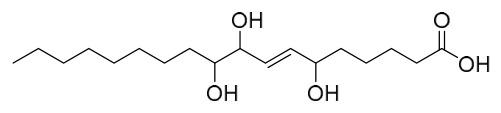 Sanleng acid	C_18_H_34_O_5_	330.2406	Tieling, Liaoning Province, China	IR, ^1^H NMR, ^13^C-NMR, ESI-MS, HR-ESI-MS	[38]
94.	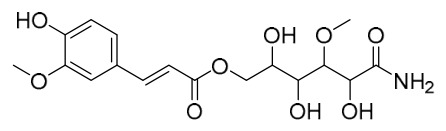 Amurenamide A	C_17_H_23_NO_9_	385.1373	Tieling, Liaoning province, China	IR, ^1^H NMR, ^13^C-NMR, ESI-MS, HR-ESI-MS	[38]
95.	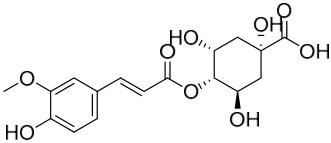 4-*O*-Feruloylquinic acid	C_17_H_20_O_9_	368.1107	Yichun, Wuchang, Mudanjiang, Heilongjiang Province; Yanbian, Tonghua, Jiaohe, Huadian, Jilin Province; Fengcheng, Anshan, Zhuanghe, Liaoning Province, China	HPLC-DAD-ESI-MS/MS	[39]
96.	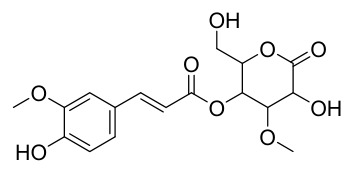 Amurenlactone A	C_17_H_20_O_9_	368.1107	Toho University herbal garden Funabashi-shi, Chiba, Japan	HR-ESI-MS, ^1^H NMR,^13^C NMR, ${\left[\alpha \right]}_{D}^{25}$	[32]
97.	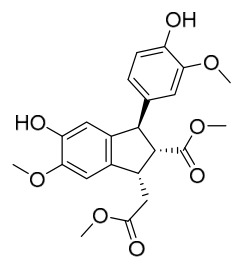 *α*-Di(methyl ferulate)	C_22_H_24_O_8_	416.1471	Toho University herbal garden Funabashi-shi, Chiba, Japan	HR-ESI-MS, ^1^H NMR,^13^C NMR, ${\left[\alpha \right]}_{D}^{25}$	[32]
98.	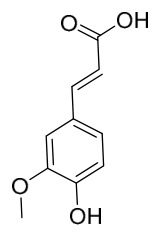 Ferulic acid	C_10_H_10_O_4_	194.0579	Harbin Tongrentang Drug Store Harbin, Heilongjiang Province, China	UPLC-ESI-Q-TOF-MS	[33]

LC-ESI-MS/MS: Liquid chromatography–electrospray ionization–tandem mass spectrometry/mass spectrometry; ^13^C NMR: Carbon-13 nuclear magnetic resonance spectrometry; ^1^H NMR: Hydrogen-1 nuclear magnetic resonance spectrometry; HR-ESI-MS: High-resolution electrospray ionization mass spectrometry; UV: Ultraviolet spectrophotometry; IR: Infrared spectroscopy; ESI-MS: Electrospray ionization mass spectrometry; UPLC-ESI-Q-TOF-MS: Ultra-high-performance liquid chromatography–electrospray ionization–quadrupole time-of-flight–mass spectrometry.

**Table 3:** Terpenoids isolated or identified from *Phellodendron amurense* Rupr.

No.	Name	Formula	Exact Theoretical Molecular Weight	Collection Site/ Material Sources	Characterization Method	Ref.
99.	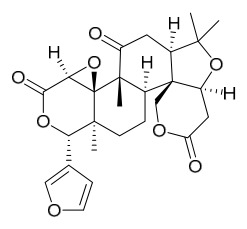 Limonin	C_26_H_30_O_8_	470.1941	Kyungdong herbal market of Korea Seoul, South Korea	UV, IR, MS, ^1^H NMR, ^13^C NMR	[29]
100.	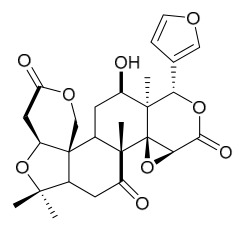 12*α*-Hydroxylimonin	C_26_H_30_O_9_	486.1890	Kyungdong herbal market of Korea Seoul, South Korea	UV, IR, MS, ^1^H NMR, ^13^C NMR	[29]
101.	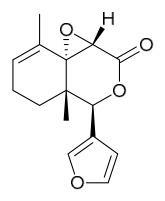 Calodendrolide	C_15_H_16_O_4_	260.1049	Gyeongsangnam-do Southern Forest Research Center, Jinju, Korea	UV, IR, ^1^H NMR, ^13^C NMR	[36]
102.	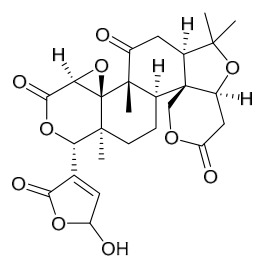 Limonexic acid	C_26_H_30_O_10_	502.1839	Toho University herbal garden Funabashi-shi, Chiba, Japan	HR-ESI-MS, ^1^H NMR,^13^C NMR, ${\left[\alpha \right]}_{D}^{25}$	[32]
103.	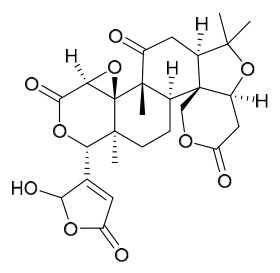 Isolimonexic acid	C_26_H_30_O_10_	502.1839	Toho University herbal garden Funabashi-shi, Chiba, Japan	HR-ESI-MS, ^1^H NMR,^13^C NMR, ${\left[\alpha \right]}_{D}^{25}$	[32]
104.	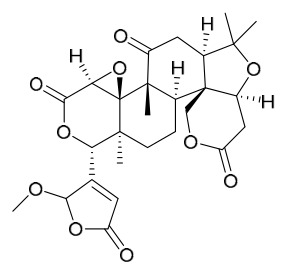 Isolimonexic acid methyl ether	C_27_H_32_O_10_	516.1995	Toho University herbal garden Funabashi-shi, Chiba, Japan	HR-ESI-MS, ^1^H NMR,^13^C NMR, ${\left[\alpha \right]}_{D}^{25}$	[32]
105.	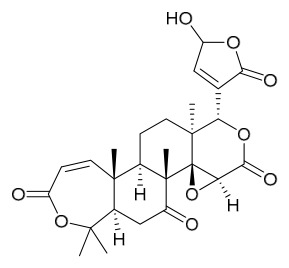 Kihadanin B	C_26_H_30_O_9_	486.1890	Tieling, Liaoning Province, China	IR, ^1^H NMR, ^13^C-NMR, ESI-MS, HR-ESI-MS	[38]
106.	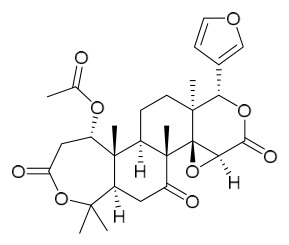 Nomilin	C_28_H_34_O_9_	514.2203	Wakaama, Japan	HPLC	[40]
107.	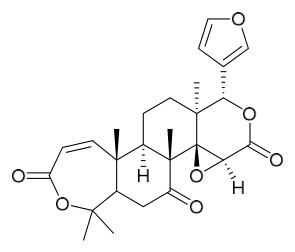 Obacunone	C_26_H_30_O_7_	454.1992	Liaoning, Hebei, Heilongjiang and Jilin Provinces of China	LC-ESI-MS/MS	[30]
108.	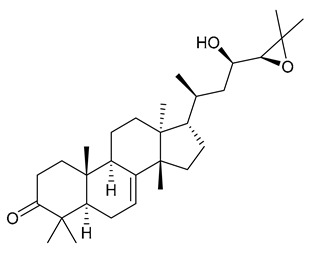 Niloticin	C_30_H_48_O_3_	456.3603	Harbin Tongrentang Drug Store Harbin, Heilongjiang Province, China	HPLC, ^1^H NMR	[41]
109.	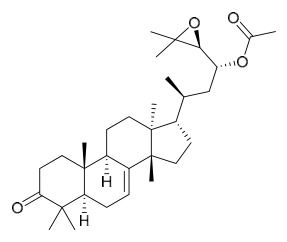 Niloticin acetate	C_32_H_50_O_4_	498.3709	Harbin Tongrentang Drug Store Harbin, Heilongjiang Province, China	HPLC, ^1^H NMR	[41]

UV: Ultraviolet spectrophotometry; IR: Infrared spectroscopy; MS: Mass spectrometry; ^13^C NMR: Carbon-13 nuclear magnetic resonance spectrometry; ^1^H NMR: Hydrogen-1 nuclear magnetic resonance spectrometry; HR-ESI-MS: High-resolution electrospray ionization mass spectrometry; ESI-MS: Electrospray ionization mass spectrometry; LC-ESI-MS/MS: Liquid chromatography–electrospray ionization–tandem mass spectrometry/mass spectrometry; HPLC: High-performance liquid chromatography.

**Table 4:** Steroids isolated or identified from *Phellodendron amurense* Rupr.

No.	Name	Formula	Exact Theoretical Molecular Weight	Collection Site/ Material Sources	Characterization Method	Ref.
110.	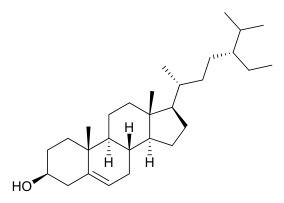 Daucosterol	C_29_H_50_O	414.3862	Tieling, Liaoning Province, China	IR, ^1^H NMR, ^13^C-NMR, ESI-MS, HR-ESI-MS	[38]
111.	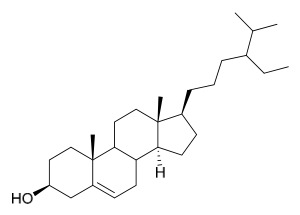 *β*-Sitosterol	C_28_H_48_O	400.3705	Tieling, Liaoning Province, China	IR, ^1^H NMR, ^13^C-NMR, ESI-MS, HR-ESI-MS	[38]
112.	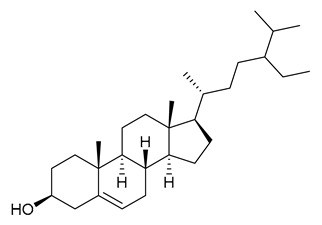 *γ*-Sitosterol	C_29_H_50_O	414.3862	Japan	UV, ${\left[\alpha \right]}_{D}^{25}$	[42]
113.	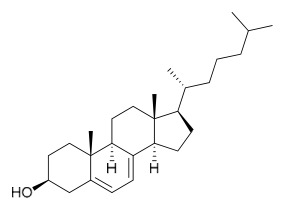 7-Dehydrocholesterol	C_27_H_44_O	384.3392	Japan	UV, ${\left[\alpha \right]}_{D}^{25}$	[42]
114.	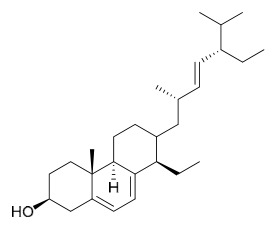 7-Dehydrostigmasterol	C_28_H_46_O	398.3549	Japan	UV	[43]

UV: Ultraviolet spectrophotometry; IR: Infrared spectroscopy; ^13^C NMR: Carbon-13 nuclear magnetic resonance spectrometry; ^1^H NMR: Hydrogen-1 nuclear magnetic resonance spectrometry; ESI-MS: Electrospray ionization mass spectrometry; HR-ESI-MS: High-resolution electrospray ionization mass spectrometry.

**Table 5:** Lignans isolated or identified from *Phellodendron amurense* Rupr.

No.	Name	Formula	Exact Theoretical Molecular Weight	Collection Site/ Material Sources	Characterization Method	Ref.
115.	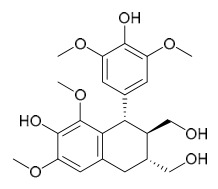 (±)-Lyoniresinol	C_22_H_28_O_8_	420.1784	Nagano Prefecture, Japan	^1^H NMR, ^13^C NMR	[35]
116.	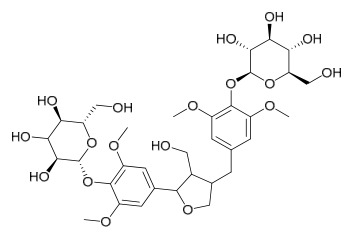 Salvadoraside	C_34_H_48_O_18_	744.2841	Naemome Dah, Ulsan, Korea	UV, ESI-MS, ^1^H NMR, ^13^C NMR	[37]
117.	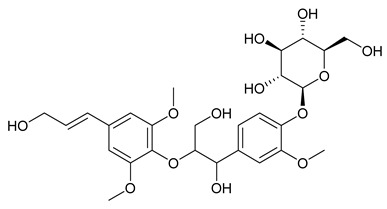 Citrusin B	C_27_H_36_O_13_	568.2156	Naemome Dah, Ulsan, Korea	UV, ESI-MS, ^1^H NMR, ^13^C NMR	[37]
118.	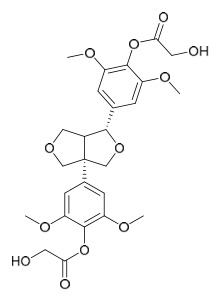 Syringaresinol di-*O*-*β*-*D*-glucopyranoside	C_26_H_30_O_12_	534.1737	Nagano Prefecture, Japan	^1^H NMR, ^13^C NMR	[35]

UV: Ultraviolet spectrophotometry; ESI-MS: Electrospray ionization mass spectrometry; ^13^C NMR: Carbon-13 nuclear magnetic resonance spectrometry; ^1^H NMR: Hydrogen-1 nuclear magnetic resonance spectrometry.

**Table 6:** Flavonoids isolated or identified from *Phellodendron amurense* Rupr.

No.	Name	Formula	Exact Theoretical Molecular Weight	Collection Site/ Material Sources	Characterization Method	Ref.
119.	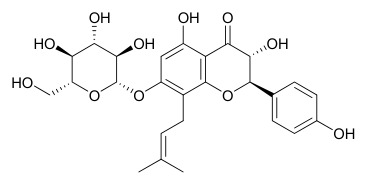 Phellamurin	C_26_H_30_O_11_	518.1788	Maritime Territory, Vladivostok, Russia	TLC, mp.	[44]
120.	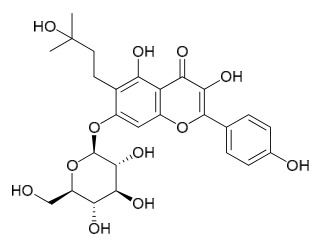 Phellavin	C_26_H_30_O_12_	534.1737	Maritime Territory, Vladivostok, Russia	TLC, mp.	[44]
121.	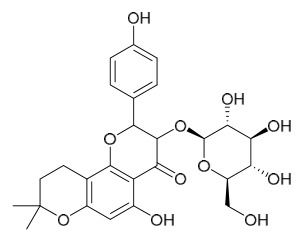 Phellodendroside	C_26_H_30_O_11_	518.1788	Maritime Territory, Vladivostok, Russia	TLC, mp.	[44]
122.	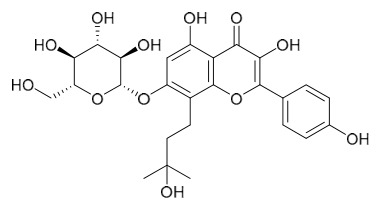 Amurensin	C_26_H_30_O_12_	534.1737	Harbin Tongrentang Drug Store Harbin, Heilongjiang Province, China	HPLC, ^1^H NMR	[41]
123.	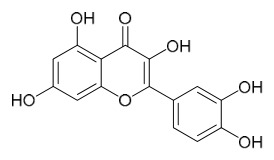 Quercetin	C_15_H_10_O_7_	302.0427	Tokushima Prefecture, Japan	HPLC, ^1^H NMR, EI-MS, HR-EI-MS	[45]
124.	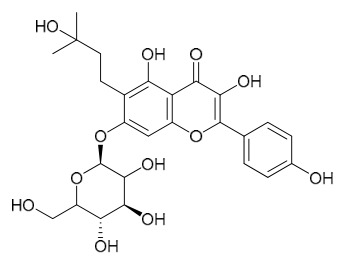 Phellatin	C_26_H_30_O_12_	534.1737	Harbin Tongrentang Drug Store Harbin, Heilongjiang Province, China	HPLC, ^1^H NMR	[41]
125.	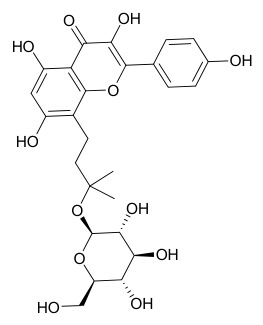 Noricariside	C_26_H_30_O_12_	534.1737	Harbin Tongrentang Drug Store Harbin, Heilongjiang Province, China	HPLC, ^1^H NMR	[41]

TLC: Thin layer chromatography; mp: Melting point; HPLC: High-performance liquid chromatography; ^1^H NMR: Hydrogen-1 nuclear magnetic resonance spectrometry; EI-MS: Electron impact mass spectrometry; HR-EI-MS: High-resolution electron impact mass spectrometry.

**Table 7:** Volatile compounds isolated or identified from *Phellodendron amurense* Rupr.

No.	Name	Formula	Exact Theoretical Molecular Weight	Collection Site/ Material Sources	Characterization Method	Ref.
126.	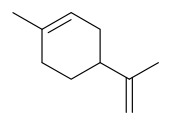 Limonene	C_10_H_16_	136.1252	Minsheng Pharmacy, Jiamusi, Heilongjiang Province, China	GC-MS, comparative analysis	[46]
127.	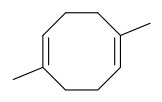 1,5-Di-methyl-1,5-cyclooctadiene	C_10_H_16_	136.1252	Minsheng Pharmacy, Jiamusi, Heilongjiang Province, China	GC-MS, comparative analysis	[46]
128.	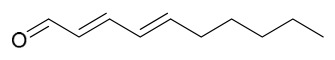 *Trans*, *trans*-2,4-decadienal	C_10_H_16_O	152.1201	Minsheng Pharmacy, Jiamusi, Heilongjiang Province, China	GC-MS, comparative analysis	[46]
129.	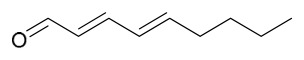 *Trans*, *trans*-2,4-nonadienal	C_9_H_14_O	138.1045	Minsheng Pharmacy, Jiamusi, Heilongjiang Province, China	GC-MS, comparative analysis	[46]
130.	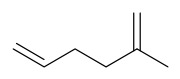 2-Methyl-1,5 hexadiene	C_7_H_12_	96.0939	Minsheng Pharmacy, Jiamusi, Heilongjiang Province, China	GC-MS, comparative analysis	[46]
131.	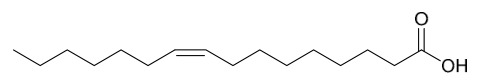 *Cis*-9-hexadecenoic acid	C_16_H_30_O_2_	254.2246	Minsheng Pharmacy, Jiamusi, Heilongjiang Province, China	GC-MS, comparative analysis	[46]
132.	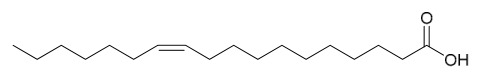 *Cis*-11-vaccenic acid	C_18_H_34_O_2_	282.2559	Minsheng Pharmacy, Jiamusi, Heilongjiang Province, China	GC-MS, comparative analysis	[46]
133.	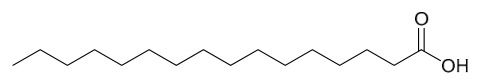 Hexadecanoic acid	C_16_H_32_O_2_	256.2402	Minsheng Pharmacy, Jiamusi, Heilongjiang Province, China	GC-MS, comparative analysis	[46]
134.	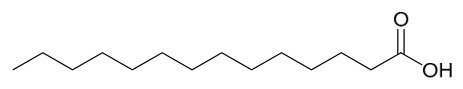 Tetradecanoic acid	C_14_H_28_O_2_	228.2089	Minsheng Pharmacy, Jiamusi, Heilongjiang Province, China	GC-MS, comparative analysis	[46]
135.	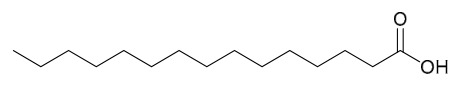 Pentadecanoic acid	C_15_H_30_O_2_	242.2246	Minsheng Pharmacy, Jiamusi, Heilongjiang Province, China	GC-MS, comparative analysis	[46]
136.	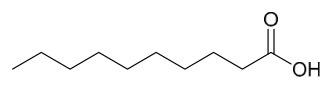 Decanoic acid	C_10_H_20_O_2_	172.1463	Minsheng Pharmacy, Jiamusi, Heilongjiang Province, China	GC-MS, comparative analysis	[46]
137.	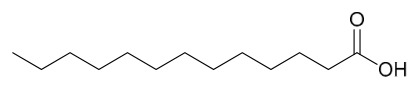 Tridecanoic acid	C_13_H_26_O_2_	214.1933	Minsheng Pharmacy, Jiamusi, Heilongjiang Province, China	GC-MS, comparative analysis	[46]
138.	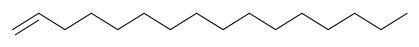 1-Hexadecene	C_16_H_32_	224.2504	Minsheng Pharmacy, Jiamusi, Heilongjiang Province, China	GC-MS, comparative analysis	[46]
139.	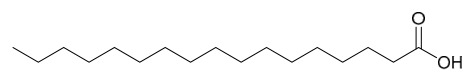 Heptadecanoic acid	C_17_H_34_O_2_	270.2559	Minsheng Pharmacy, Jiamusi, Heilongjiang Province, China	GC-MS, comparative analysis	[46]
140.	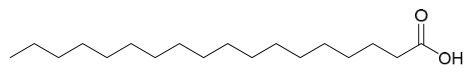 Octadecanoic acid	C_18_H_36_O_2_	284.2715	Minsheng Pharmacy, Jiamusi, Heilongjiang Province, China	GC-MS, comparative analysis	[46]
141.	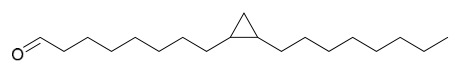 2-Octylcyclopropaneoctanal	C_19_H_36_O	280.2766	Minsheng Pharmacy, Jiamusi, Heilongjiang Province, China	GC-MS, comparative analysis	[46]
142.	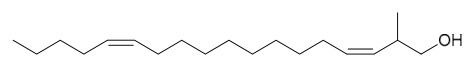 (3*Z*,13*Z*)-2-Methyl-3,13-octadecadien-1-ol	C_19_H_36_O	280.2766	Minsheng Pharmacy, Jiamusi, Heilongjiang Province, China	GC-MS, comparative analysis	[46]
143.	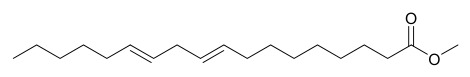 9,12-Octadecadienoic acid (*Z*,*Z*)	C_19_H_34_O_2_	294.2559	Minsheng Pharmacy, Jiamusi, Heilongjiang Province, China	GC-MS, comparative analysis	[46]
144.	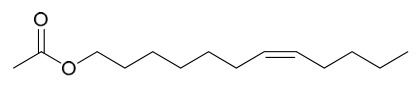 Cis-7-dodecen-1-ylacetate	C_14_H_26_O_2_	226.1933	Minsheng Pharmacy, Jiamusi, Heilongjiang Province, China	GC-MS, comparative analysis	[46]
145.	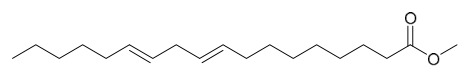 9,12-Octadecadienoic acid, methyl ester	C_19_H_34_O_2_	294.2559	Minsheng Pharmacy, Jiamusi, Heilongjiang Province, China	GC-MS, comparative analysis	[46]
146.	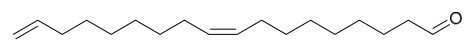 9,17-Octadecadienal,(*Z*)	C_18_H_32_O	264.2453	Minsheng Pharmacy, Jiamusi, Heilongjiang Province, China	GC-MS, comparative analysis	[46]
147.	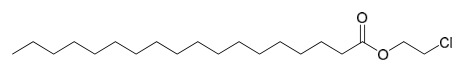 2-Chloroethyl linoleate	C_20_H_39_CIO_2_	346.2639	Minsheng Pharmacy, Jiamusi, Heilongjiang Province, China	GC-MS, comparative analysis	[46]
148.	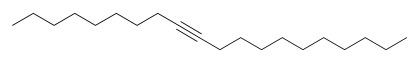 9-Eicosyne	C_20_H_38_	278.2974	Minsheng Pharmacy, Jiamusi, Heilongjiang Province, China	GC-MS, comparative analysis	[46]
149.	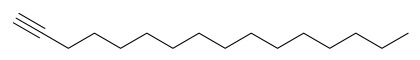 1-Hexadecyne	C_16_H_30_	222.2348	Minsheng Pharmacy, Jiamusi, Heilongjiang Province, China	GC-MS, comparative analysis	[46]
150.	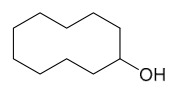 1-Hydroxycyclodecane	C_10_H_20_O	156.1514	Minsheng Pharmacy, Jiamusi, Heilongjiang Province, China	GC-MS, comparative analysis	[46]
151.	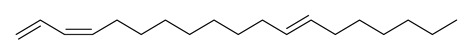 *E*,*Z*-1,3,12-Nonadecatriene	C_19_H_34_	262.2661	Minsheng Pharmacy, Jiamusi, Heilongjiang Province, China	GC-MS, comparative analysis	[46]
152.	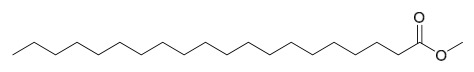 11,14-Eicosadienoic acid, methyl ester	C_21_H_42_O_2_	326.3185	Minsheng Pharmacy, Jiamusi, Heilongjiang Province, China	GC-MS, comparative analysis	[46]
153.	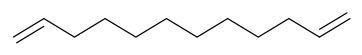 1,11-Dodecadiene	C_12_H_22_	166.1722	Minsheng Pharmacy, Jiamusi, Heilongjiang Province, China	GC-MS, comparative analysis	[46]
154.	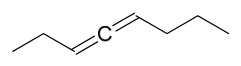 7-Methyl-3,4-octadiene	C_8_H_14_	110.1096	Minsheng Pharmacy, Jiamusi, Heilongjiang Province, China	GC-MS, comparative analysis	[46]
155.	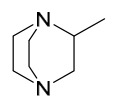 2-Methyl-1,4-diazabicyclo[2.2.2]octane	C_7_H_14_N_2_	126.1157	Minsheng Pharmacy, Jiamusi, Heilongjiang Province, China	GC-MS, comparative analysis	[46]
156.	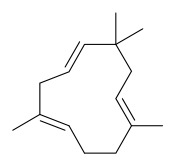 *α*-Humulene	C_15_H_24_	204.1878	Tongrentang Pharmacy Co., Ltd. Shenyang, Liaoning Province, China	HPLC-MS, MS-DIAL, and Cytoscape Analysis	[28]

GC-MS: Gas chromatography–mass spectrometry; HPLC-MS: High-performance liquid chromatography–mass spectrometry; MS-DIAL: Mass spectrometry-data independent analysis.

**Table 8:** Other compounds isolated or identified from *Phellodendron amurense* Rupr.

No.	Name	Formula	Exact Theoretical Molecular Weight	Collection Site/ Material Sources	Characterization Method	Ref.
157.	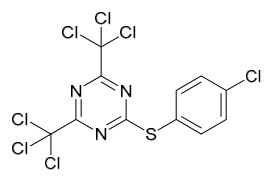 2-[(4-Chlorophenyl)sulfanyl]-4,6-bis(trichloromethyl)-1,3,5-triazine	C_11_H_4_Cl_7_N_3_S	454.7946	China	GC-MS	[47]
158.	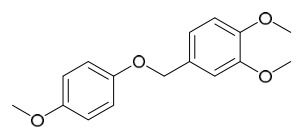 1,2-Dimethoxy-4-[(4-methoxyphenoxy)methyl]benzene	C_16_H_18_O_4_	274.1205	China	GC-MS	[47]
159.	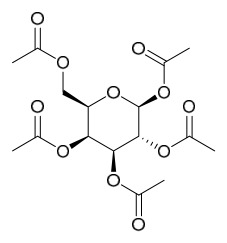 *β*-*D*-Galactopyranose pentaacetate	C_16_H_22_O_11_	390.1162	China	GC-MS	[47]
160.	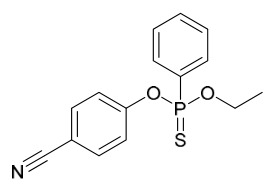 Cyanophenphos	C_15_H_14_NO_2_PS	303.0483	China	GC-MS	[47]
161.	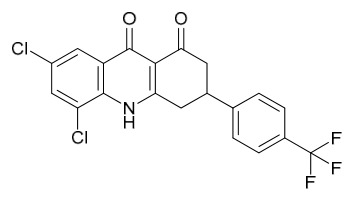 5,7-Dichloro-3,4-dihydro-3-[4-[trifluoromethyl]phenyl]-1,9(2H,10H)-acridinedione	C_20_H_12_Cl_2_F_3_NO_2_	425.0197	China	GC-MS	[47]
162.	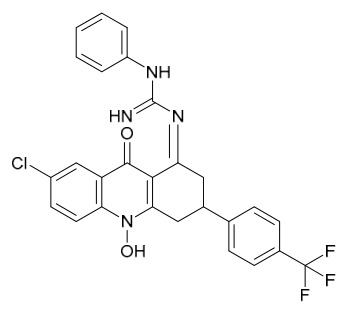 2-[7-Chloro-3,4,9,10-tetrahydro-10-hydroxy-9-oxo-3-[4-trifluoromethyl]-1(2H)-acridinelideneamino]-1-phenylguanidine	C_27_H_20_ClF_3_N_4_O_2_	524.1227	China	GC-MS	[47]
163.	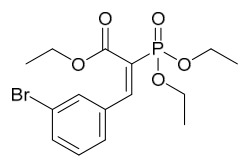 3-(3-Bromophenyl)propenoic acid, 2-(diethoxyphosphinyl)-, ethyl ester	C_15_H_20_BrO_5_P	390.0232	China	GC-MS	[47]
164.	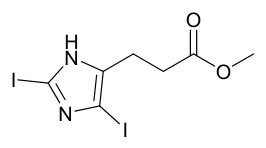 2,4-Diiodoimidazole-5-propionic acid, methyl ester	C_7_H_8_I_2_N_2_O_2_	405.8675	China	GC-MS	[47]
165.	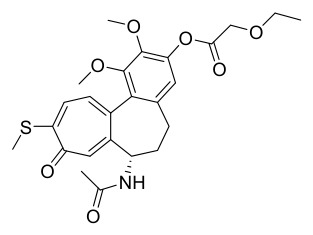 3-Ethoxyacetyl-3-demethylthiocolchicine	C_25_H_29_NO_7_S	487.1665	China	GC-MS	[47]
166.	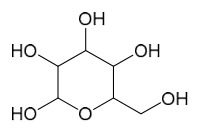 *D*-Galactopyranose	C_6_H_12_O_6_	180.0634	Tongrentang Pharmacy Co., Ltd. Shenyang, Liaoning Province, China	HPLC-MS, MS-DIAL, and Cytoscape analysis	[28]
167.	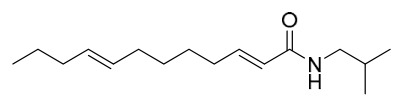 Herculin	C_16_H_29_NO	251.2249	Harbin Tongrentang Drug Store Harbin, Heilongjiang Province, China	UPLC-ESI-Q-TOF-MS	[33]
168.	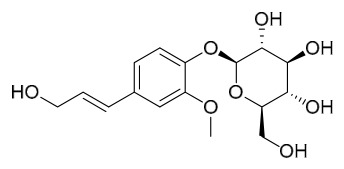 Coniferin	C_16_H_22_O_8_	342.1315	Nagano Prefecture, Japan	^1^H NMR, ^13^C NMR	[35]
169.	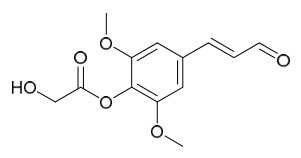 Sinapic aldehyde 4-*O*-*β*-*D*-glucopyranoside	C_13_H_14_O_6_	266.0790	Nagano Prefecture, Japan	^1^H NMR, ^13^C NMR	[35]
170.	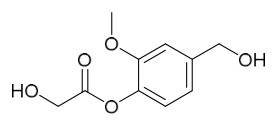 Vanilloloside	C_10_H_12_O_5_	212.0685	Nagano Prefecture, Japan	^1^H NMR, ^13^C NMR	[35]

GC-MS: Gas chromatography–mass spectrometry; HPLC-MS: High-performance liquid chromatography–mass spectrometry; MS-DIAL: Mass spectrometry-data independent analysis; UPLC-ESI-Q-TOF-MS: Ultra-high-performance liquid chromatography–electrospray ionization–quadrupole time-of-flight–mass spectrometry; ^13^C NMR: Carbon-13 nuclear magnetic resonance spectrometry; ^1^H NMR: Hydrogen-1 nuclear magnetic resonance spectrometry.

**Table 9:** Synthesis of major active compounds, common signaling pathways, and recurring therapeutic themes in the pharmacology of *Phellodendri amurensis* Cortex.

Recurring Therapeutic Theme	Major Active Compounds Involved	Common Signaling Pathways/Mechanisms	Representative Disease Models/Effects (Section)
Anti-inflammation	Berberine, palmatine, phellodendrine, magnoflorine, 4-*O*-feruloylquinic acid, obakulactone	NF-*κ*B, MAPK (p38, ERK, JNK), NLRP3 inflammasome/caspase-1, PLA2, PAR-2, JAK/STAT, PI3K/AKT	Endotoxemia (8.4), gouty arthritis (8.4, 8.11), rheumatoid arthritis (8.4), skin inflammation (8.4, 8.14), asthma (8.17)
Antioxidant stress	Total flavonoids, polysaccharides, berberine, limonin, nomilin	Nrf2/ARE, SOD/CAT/GPx upregulation, ROS scavenging, lipid peroxidation inhibition	UV-induced skin damage (8.1), diabetic nephropathy (8.2, 8.12), liver injury (8.13), diabetic hamster (8.2)
Hypoglycemic & lipid-lowering	Berberine, oleic acid, limonin, nomilin	PTP1B inhibition, AKT/insulin signaling, AMPK, glucokinase upregulation, GLUT4 expression	Type 2 diabetes (8.2), diabetic nephropathy (8.2, 8.12), high-fat diet-induced dyslipidemia (8.2)
Anticancer	Berberine, palmatine, magnoflorine, phellamurin, oxypalmatine, canthin-6-one, lotusine, oxyepiberberine, Nexrutine	PI3K/AKT/mTOR, NF-*κ*B, COX-2, STAT3, EGFR-AKT-ERK, tubulin polymerization, PARP1	Lung cancer (8.6), prostate cancer (8.6, 8.15), gastric cancer (8.6), osteosarcoma (8.6), breast cancer (8.6), colon cancer (8.6), melanoma (8.6)
Neuroprotection & memory improvement	Berberine, magnoflorine, phellodendrine, syringin	Bax/Bcl-2/caspase-3, BDNF/cAMP/CREB, IGFR/JAK/AKT, NLRP3/SWELL1, acetylcholinesterase inhibition	Alzheimer’s disease (8.5), Parkinson’s-related models (8.5), depression (8.5), scopolamine-induced memory impairment (8.5)
Organ protection (kidney, liver, gut, heart)	Berberine, palmatine, phellodendrine, dihydroberberine, PAC polysaccharide, limonin	NLRP3, NF-*κ*B, TGF-*β*/Smad, PI3K/AKT, Nrf2/ARE, ADK/AMPK, XO/ADA inhibition, urate transporter regulation	Diabetic nephropathy (8.12), hyperuricemic nephropathy (8.12), adriamycin-induced nephritis (8.12), gastric ulcer (8.9), intestinal injury (8.9), liver injury (8.13), myocardial ischemia (8.8)
Anti-gout & uric acid regulation	Berberine, palmatine, dihydroberberine, berberrubine	XO/ADA inhibition, NLRP3/caspase-1, TLR4/NF-*κ*B, Nrf2, urate transporter (URAT1, GLUT9, OAT1, ABCG2)	Acute gouty arthritis (8.11), hyperuricemia (8.11, 8.12), MSU-induced inflammation (8.4)
Antimicrobial & antifungal	Berberine hydrochloride, palmatine hydrochloride, obacunone, limonin	Cell membrane disruption, biofilm inhibition, gene regulation (PGAL4, FSH1, PQ-LRP)	*S. aureus*, *S. mutans* (8.3), *Microsporum canis* (8.3), termite antifeedant (8.16)
Immunosuppression	Phellodendrine, magnoflorine, berberine, total polysaccharides	GVH reaction inhibition, DTH afferent phase blockade, IFN-γ/IL-1 suppression, lymphocyte proliferation inhibition	Delayed-type hypersensitivity (8.6), graft-versus-host reaction (8.6)
Antiviral	Aqueous extract, niloticin, *γ*-fagarine	Type I IFN (IFN-*β*), IRF3, STAT1, NF-*κ*B, NS2B/NS3 protease inhibition, heparan sulfate binding	Influenza (H1N1, H5N2) (8.18), dengue virus (8.18), human metapneumovirus (8.18)

## Data Availability

Data are contained within the article.
